# Orthogonal Light-Activated DNA for Patterned Biocomputing within Synthetic Cells

**DOI:** 10.1021/jacs.3c02350

**Published:** 2023-04-26

**Authors:** Denis Hartmann, Razia Chowdhry, Jefferson M. Smith, Michael J. Booth

**Affiliations:** † Department of Chemistry, University of Oxford, Mansfield Road, Oxford OX1 3TA, U.K.; ‡ Department of Chemistry, University College London, 20 Gordon Street, London WC1H 0AJ, U.K.

## Abstract

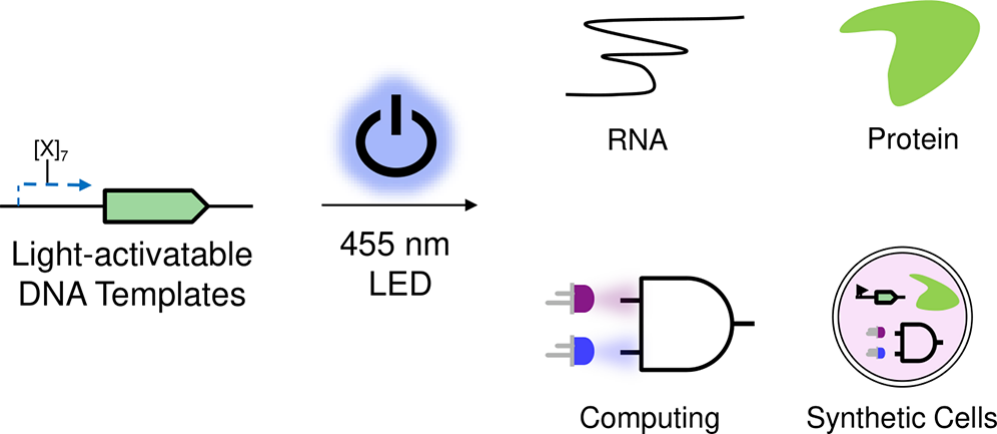

Cell-free gene expression is a vital research tool to study biological systems in defined minimal environments and has promising applications in biotechnology. Developing methods to control DNA templates for cell-free expression will be important for precise regulation of complex biological pathways and use with synthetic cells, particularly using remote, nondamaging stimuli such as visible light. Here, we have synthesized blue light-activatable DNA parts that tightly regulate cell-free RNA and protein synthesis. We found that this blue light-activated DNA could initiate expression orthogonally to our previously generated ultraviolet (UV) light-activated DNA, which we used to generate a dual-wavelength light-controlled cell-free AND-gate. By encapsulating these orthogonal light-activated DNAs into synthetic cells, we used two overlapping patterns of blue and UV light to provide precise spatiotemporal control over the logic gate. Our blue and UV orthogonal light-activated DNAs will open the door for precise control of cell-free systems in biology and medicine.

## Introduction

Precise control of gene expression has a wide range of applications, including in biological research, biotechnology, and medicine.^1^ One area of gene expression that lacks tools for control is cell-free expression (CFE), which produces functional RNA/protein from a DNA template. CFE is widely used in biology, biotechnology, and synthetic biology^2,3^ as a research tool to study fundamental biological processes in a minimal, cell-like environment.^4,5^ Several important biological mechanisms, such as DNA replication,^6,7^ genetic code,^8^ and role of mRNA poly-A tails,^9^ have been elucidated using CFE systems. A large number of different CFE systems have been developed^10−12^ with modern systems offering high expression yields, versatility, scalability, and accessibility. Biosensors based on CFE logic gates have been employed to generate portable detection systems for pathogens^13−15^ and small molecules.^16−18^ CFE has also allowed for the rapid and high-yielding production of mRNA vaccines required for large-scale vaccination efforts against SARS-CoV-2.^19,20^ Encapsulation of a CFE system within a lipid bilayer has also been used to form synthetic cells,^21−24^ allowing for a bottom-up approach toward studying biological processes such as cellular communication^25−27^ and the cell cycle^28,29^ in vitro and has future applications in drug delivery through interactions with living cells.^30^

The ability to control gene expression in CFE systems will allow the reconstitution of more complex biological pathways for fundamental biological research,^31,32^ as well as targeted interactions between synthetic cells and living cells.^33^ To achieve control over these processes, the DNA template in question can be modified to respond to a range of stimuli, including changes in pH,^34^ redox potential,^35−37^ temperature,^38^ and light.^39^ Light as a stimulus is particularly attractive, as it is applied remotely and is largely bioorthogonal, and has already found widespread use in the control of DNA or RNA function.^39−41^ This has often been achieved by the attachment of photoactive chemical moieties (“photocages”) to the DNA to inhibit its function before illumination. Our group has previously developed a light-activatable caging system for DNA parts used in CFE systems.^42^ Transcription from a T7 promoter of a DNA template was blocked by seven monovalent streptavidin (mSA), attached to the DNA via UV-photocleavable (2-nitrobenzyl) biotin linkers. Illumination with ultraviolet (UV) light released the streptavidins, allowing T7 RNA polymerase to bind to the promoter, thereby activating transcription. Beyond photocages, photoreversible systems based on azobenzene-modified DNA photoswitches have been generated, but suffer from leaky off- and poor on-states,^43^ while systems based on light-sensitive proteins also suffer from leaky off states and require co-expression of additional genes. Our photocage approach is simple to synthesize, general to any gene of interest, and requires no auxiliary protein to be expressed.^44^

Most light-activatable DNAs rely on UV photocages. Longer-wavelength photocages would be preferred, however, as visible light shows reduced damage to biomolecules^45,46^ and tissue penetration is increased with longer wavelengths.^47,48^ Longer-wavelength photocages for use in biological systems have been reported.^49^ An additional advantage of light is the possibility to use multiple wavelengths of light to control different DNAs. To this end, several reports have shown sequential activation of two photocages.^50−52^ However, orthogonal activation of two or more photocages has few reported examples,^53−57^ mostly due to chromophores having overlapping absorbance bands and orthogonal release thus being difficult. There is a clear need to develop longer-wavelength/orthogonal light-activated DNA parts for use in CFE.

**Figure 1:**
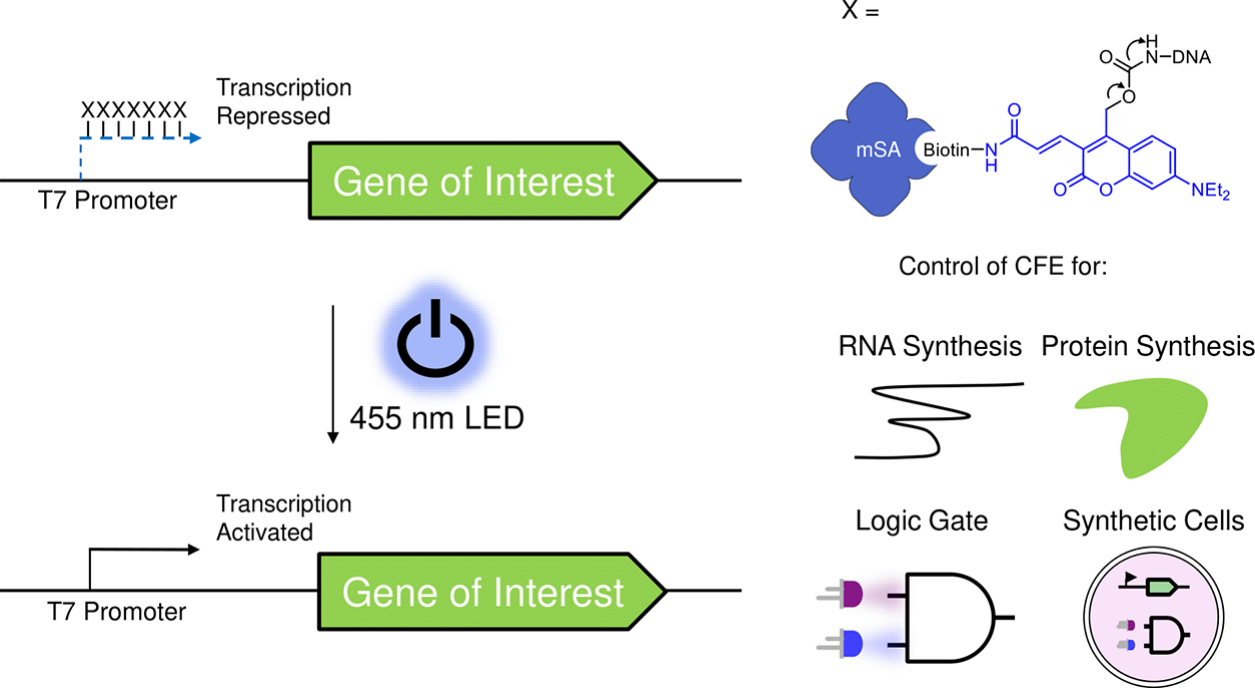
Caged, blue light-activatable DNA for control of cell-free expression (CFE). An amine-modified T7 promoter upstream of a gene of interest was modified with a biotinylated coumarin derivative. The binding of monovalent streptavidin (mSA) provided the necessary steric bulk to repress transcription from the DNA template. Upon illumination with a 455 nm light-emitting diode (LED), this steric bulk was cleaved off and transcription activated. This blue light-activatable DNA allowed for the control of RNA and protein synthesis in a CFE system and enabled the construction of an AND gate with 2 wavelengths as inputs, which was spatiotemporally controlled in synthetic cells.

Here, we have built on our previous light-activated DNA design to create blue light-activatable DNA parts, by incorporating an extended coumarin photocage (Figure 1).^53,58^ This blue light-activated DNA (bLA–DNA) shows a very tight off-state, rapid photocleavage upon 455 nm illumination, and allows for the light-controlled cell-free synthesis of RNA and protein. Most importantly, this bLA–DNA is orthogonal to the previously developed ultraviolet LA–DNA (uvLA–DNA). We deployed these two LA–DNAs together to create the first cell-free expression logic gate controlled with two orthogonal wavelengths of light as inputs. Following pre-printing of this manuscript, two-wavelength photocleavage of DNA strands was used to create logic gates based on DNA hybridization;^59^ however, the photocages had to be installed during solid-phase synthesis and the gates had a high off-state. Our blue and UV LA–DNAs were encapsulated into synthetic cells, and we used two overlapping patterns of light to spatiotemporally control the cell-free logic gate. Remote control of cell-free expression and biocomputing using these orthogonal light-activated DNAs will open new possibilities in cell-free biology.

## Results and Discussion

### Synthesis of a Blue Light-Photocleavable Group Carrying a Biotin

We set out to synthesize a blue light-activatable group able to react with amino-functionalized DNA to expand and improve on our existing light-activatable DNA (LA–DNA) technology. This blue light-activatable group carries a biotin motif linked via a short glycol-derived linker to bind to streptavidin, providing the steric bulk necessary for caging the DNA.

For this, we chose the widely studied diethylaminocoumarin (DEACM) scaffold, which shows excellent photocleavage upon UV irradiation^60,61^ and has been used to control several biological processes.^51,62,63^ An extended DEACM scaffold has been reported,^58^ which shifted its absorption maximum to ∼450 nm. This extended scaffold has been used in biological systems,^64^ shows orthogonality to UV light irradiation,^53^ and contains a useful attachment point for the biotin functionalization at the acrylate moiety.

We initially synthesized scaffold **4** using published procedures.^58^ Following deprotection of the *t*-butyl-group on the acrylate using TFA/DCM, peptide coupling using EDC·HCl with a mono-Boc-protected, triethylene-glycol-derived diamine (S1, Supporting Information) yielded the PEG-ylated coumarin **5** in excellent yield, which provides a versatile intermediate for further derivatization. Following this, Boc-deprotection with TFA/DCM and the addition of biotin-*N*-hydroxysuccinimide ester (Biotin-NHS, S2, Supporting Information) in DMF under basic conditions yielded the biotinylated coumarin in good yields. In both deprotection steps, we observed partial removal of the TBDMS group through TFA, initially installed to prevent side reactions with the alcohol during the peptide coupling step, as well as reaction with the Biotin-NHS, but the TBDMS-deprotected compounds worked well in the same reactions and caused no apparent issues. After deprotection of the TBDMS group with TBAF, the resulting alcohol **7** was converted into active carbonates for reaction with the amino-functionalized DNA, and their photochemical properties were studied (Supporting Figures S1–S5).

**Figure 2:**
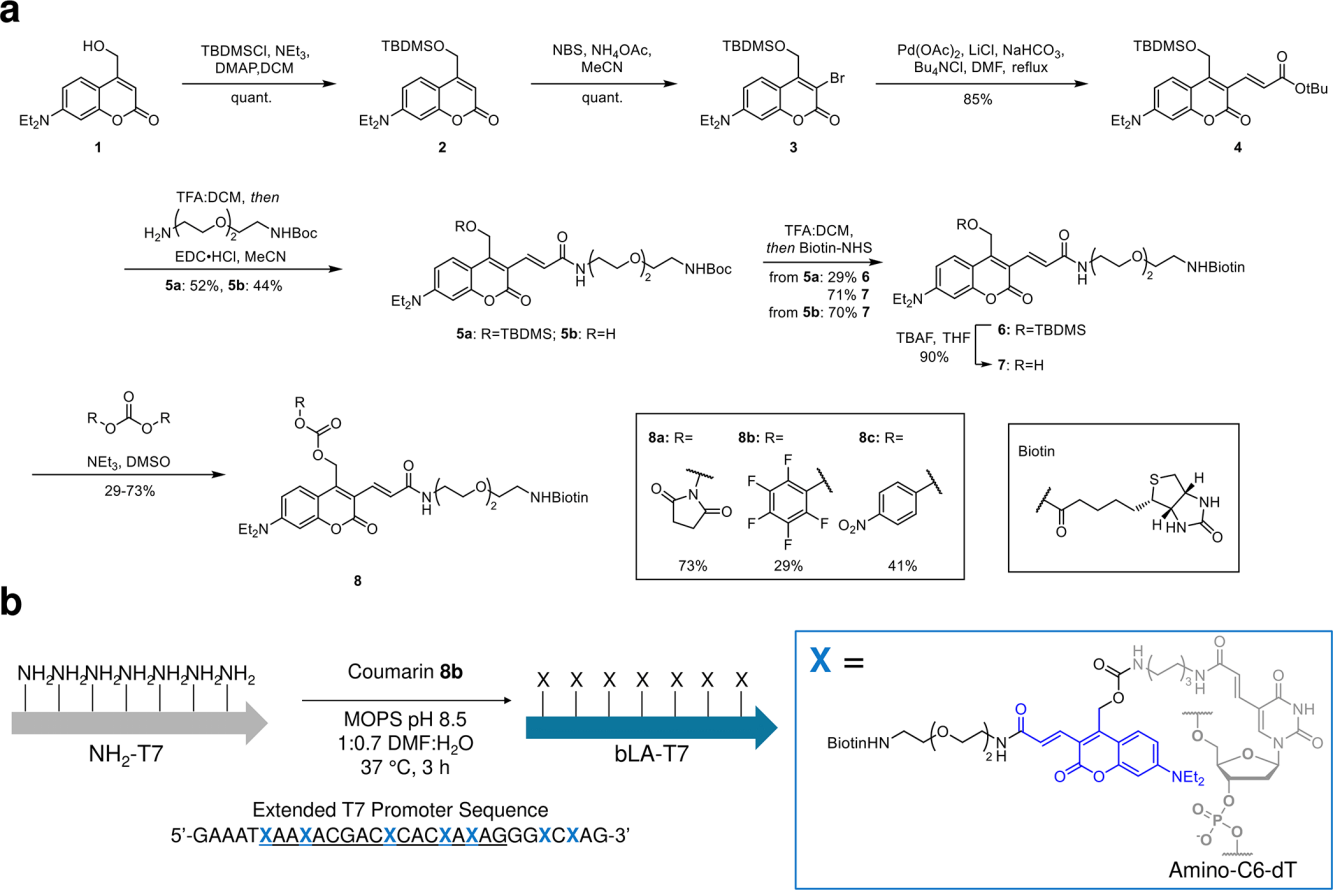
Synthesis of the biotinylated coumarin-based photocage and its reaction with DNA. (a) Synthetic scheme describing the synthesis of the biotinylated coumarin active carbonates. (b) Optimal conditions for the reaction of the 7-amino-C6-dT-modified ssDNA with the coumarin photocage. TBDMS = *tert*-Butyldimethylsilyl-, DMAP = 4-dimethylaminopyridine, DCM = dichloromethane, NBS = *N*-bromosuccinimide, DMF = dimethylformamide, TFA = trifluoroacetic acid, Boc = *tert*-butyloxycarbonyl, EDC = *N*-(3-dimethylaminopropyl)-*N*′-ethylcarbodiimide, NHS = *N*-hydroxysuccinimide, TBAF = tetrabutylammonium fluoride, THF = tetrahydrofuran, DMSO = dimethylsulfoxide.

The synthesized *N*-hydroxysuccinimide (NHS) carbonate **8a** sadly was completely unreactive toward DNA-containing one, four, or seven amines (Supporting Figure S6) in NaHCO_3_ buffer, which we have previously employed to attach the UV-activatable 2-nitrobenzene photocage to DNA.^42^ The pentafluorophenol (PFP) and *p*-nitrophenol (*p*NP) carbonates **8b**/**c**, due to their higher degree of stability in aqueous media, did provide reactivity in initial conditions using NaHCO_3_ and the 7-amino-modified ssDNA, as seen by denaturing poly(acrylamide) gel electrophoresis (PAGE) (Supporting Figure S7). However, we found that the PFP-Carbonate **8b** in MOPS pH 8.5 at 37 °C for 3 h gave the best conversion toward the fully modified DNA (Figure 2b and Supporting Figures S8 and S9). The modified, blue light-activatable T7 promoter DNA (bLA-T7) was purified via ion-paring high-performance liquid chromatography (IP-HPLC, Supporting Figure S13) and its presence was confirmed by liquid chromatography–mass spectrometry (LC–MS) (oligonucleotide mass spectra, Supporting Table S5) and PAGE (Supporting Figure S14). It showed greater gel retention, as well as fluorescence of the coumarin before gel-staining, indicating attachment of the desired photocage. We also synthesized a surrogate for the carbamate formed during the reaction of DNA with our molecule by reacting **8c** with propargylamine (S3, Supporting Information).

### Control of Cell-Free Expression

To test the caging ability of this new photocage on the DNA, we initially chose in vitro transcription of an RNA aptamer, broccoli, which fluoresces when bound to the small molecule DFHBI.^65^ For this, we annealed a template strand, containing a T7 promoter sequence (complementary to the bLA-T7) upstream of the broccoli aptamer sequence, with the unmodified amine containing T7 promoter sequence (as a control for 100% photocleavage) or with our modified T7 promoter, and a third strand complementary to the broccoli template (Supporting Table S3). Following this, monovalent streptavidin (mSA) was bound to the hybridized DNA (Supporting Figure S15a). We used this DNA template to express the broccoli aptamer using T7 RNA polymerase and measured the RNA output via agarose gel electrophoresis (AGE) (Supporting Figure S15b) and fluorescence spectroscopy (Supporting Figure S15c) with and without 455 nm illumination. In the fluorescence measurements, we found that the bLA–DNA itself had a high fluorescence background, due to the excitation/emission spectra of the coumarin moiety overlapping with the broccoli/DHFBI complex, which was confirmed by a no polymerase control. After taking into account the background fluorescence, we observed a tight off-state of transcription in the absence of blue light by both gel and fluorescence. Upon illumination with blue light, we saw a high recovery of expression, with only 1 min of irradiation necessary for full activation and no significant difference between 1 and 5 min, indicating negligible damage to the system using blue light.

**Figure 3:**
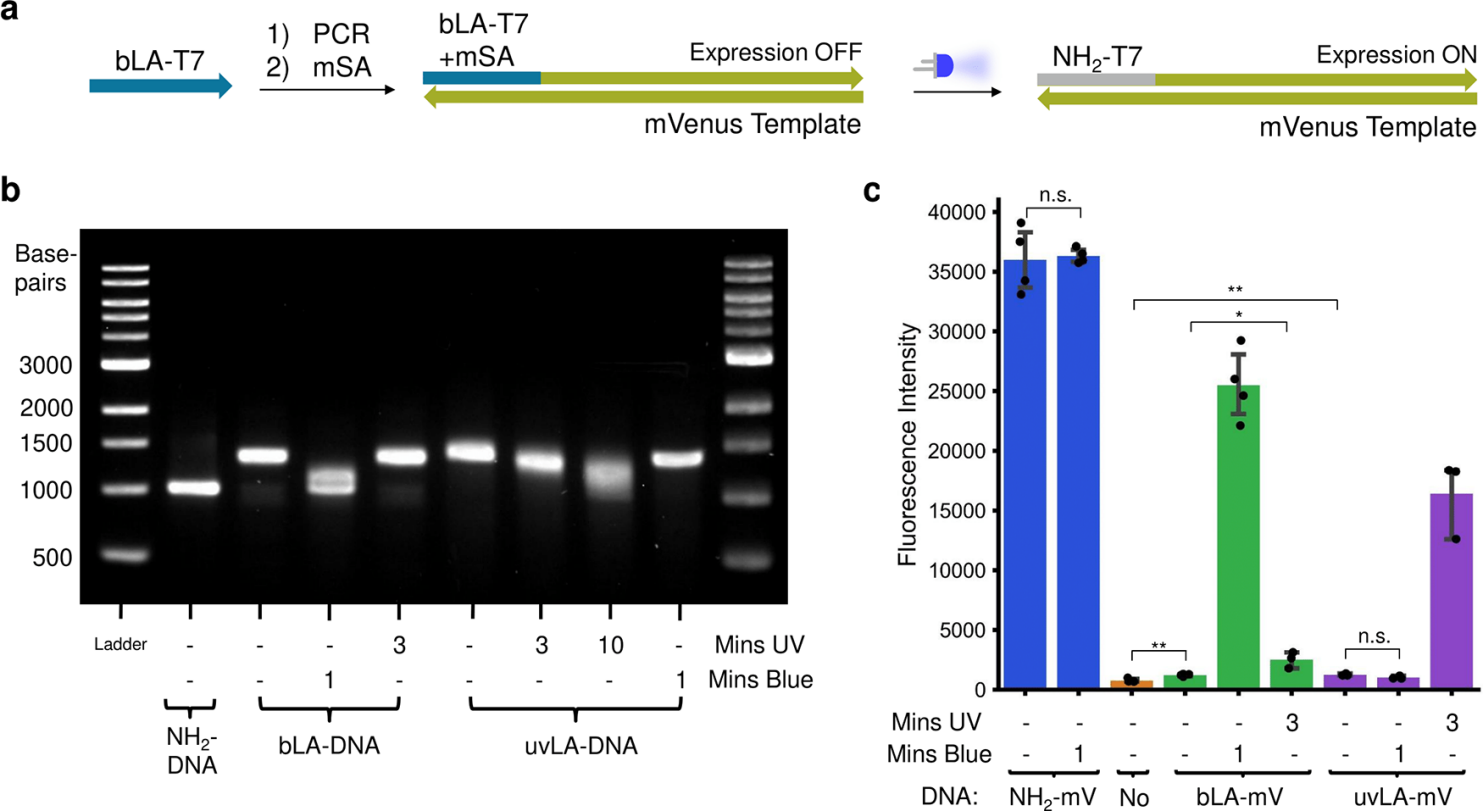
bLA- and uvLA-mVenus DNA under different illumination conditions. (a) Preparation of caged, blue light-activatable DNA and uncaging with blue light. bLA-T7 is used as a primer in PCR to generate a modified DNA template. The addition of mSA cages the DNA, preventing transcription. Illumination with blue light then cleaves off the molecule + mSA, allowing for expression from the DNA template. (b) Agarose gel electrophoresis of NH_2_-, bLA-, and uvLA-mV DNA under different illumination conditions. Modification and binding of mSA gave larger gel retention for both bLA- and uvLA-mV DNA. Illumination of bLA-mV DNA with blue light showed uncaging toward the NH_2_-mV DNA template but was not affected by UV irradiation. Illumination of uvLA–DNA with UV light showed uncaging, but illumination with blue light did not. (c) Cell-free expression of NH_2_-, bLA-, and uvLA-mV DNA with blue and UV irradiation. Both bLA– and uvLA–DNA had a tight off-state in the absence of irradiation. Illumination of bLA–DNA with a blue LED gave 70% recovery of expression, whereas illumination with UV yielded only a small increase in expression. For uvLA–DNA, we saw the reverse, where illumination with UV light gave a 44% recovery of expression, whereas illumination with blue light yielded no increase in the expression of mVenus. *n* = 4 for bLA–DNA (with bLA–DNA + UV being *n* = 3) and *n* = 3 for uvLA–DNA samples. n.s. - nonsignificant (*p*-value > 0.05). *p-value < 0.05. **p-value < 0.005.

Next, we tested the ability of this caging group for blue light-controlled cell-free protein synthesis. For this, we generated a linear DNA template encoding the fluorescent protein mVenus (mV) by PCR, using the ssDNA bLA-T7 as a forward primer, followed by the addition of mSA to cage the resulting bLA-mV DNA template (Figure 3a). We also synthesized a UV light-activatable mV DNA template (uvLA-mV DNA) using a commercial, nitrobenzyl-based photocleavable biotin (from Click-Chemistry-Tools) as previously described (Supporting Figures S10 and S11).^42^ We first analyzed the bLA-mV DNA, uvLA-mV DNA, and amino control NH_2_-mV DNA by agarose gel electrophoresis with and without illumination (Figure 3b). As expected, bLA-mV and uvLA-mV had decreased mobility through the gel, demonstrating the mSA was bound. Irradiation of the bLA-mV DNA for 1 min with a 455 nm blue LED showed almost full recovery to the control NH_2_-mV DNA band. Longer illumination did not show additional photocleavage (Supporting Figure S16), meaning further uncaging is not possible, which has previously been shown for other coumarin-based photocages.^66−68^ We also observed that upon irradiation with blue light, under identical conditions used for the bLA-mV DNA, the uvLA-mV DNA did not show any shift in the gel. Irradiation of the uvLA-mV DNA with UV light for 3 min, however, caused a small change in gel retention, indicating partial uncaging. This effect was more pronounced upon UV irradiation for 10 min. Excitingly, irradiating bLA-mV DNA for 3 min with a 365 nm UV LED showed little/no shift, indicating minimal uncaging, particularly compared to the uvLA-mV DNA counterpart. This data is in line with the UV–visible absorbance data of the LA-T7 promoters and the spectra of the LEDs employed (Supporting Figure S17). The spectrum of the blue LED showed little/no overlap with the UV–vis spectrum of the uvLA-T7 ssDNA and excellent overlap with the bLA-T7 ssDNA. Similarly, excellent overlap of the UV LED with the uvLA-T7 ssDNA tail, corresponding to the nitrobenzyl group, was observed. The bLA-T7 ssDNA spectrum has a nonzero minimum at the wavelengths of UV LED and, due to low absorption at those wavelengths, very little excitation of the coumarin chromophore to productive excited states occurred, giving rise to little photocleavage under UV irradiation, allowing for orthogonal release of DNA.

Following analysis of the gel mobility, we tested the CFE of mV from the DNA templates in response to blue and UV light. mV was synthesized from these templates using a commercial CFE system (PURExpress) and the protein output was measured by fluorescence intensity after incubation for 4 h (Figure 3c) and compared to the DNA-containing amino-C6-dT in the T7 promoter, representing a theoretical 100% photorelease. The presence of the seven amino-C6-dTs in the T7 promoter did not perturb mV expression (Supporting Figure S18). As expected, illumination of the control NH_2_-DNA for 1 min with 455 nm blue light showed no damaging effect of the blue irradiation on CFE (101% vs no LED control, nonsignificant, *p*-value = 0.821). Without any irradiation of bLA-mV DNA, we saw only a minor increase in fluorescence of 1.3% compared to the no DNA background (significant, *p*-value = 0.002). This is supported by looking at the RNA transcription of the bLA-mV DNA template using T7 RNA polymerase, where we saw negligible amounts of mV mRNA being formed in the absence of light (Supporting Figure S19). Upon illumination of the CFE containing our bLA-mV DNA for 1 min with blue light, we observed 70% recovery of expression compared to the NH_2_-DNA with an ON/OFF ratio for the bLA–DNA of 98%, with the same trend being observed in the mRNA. Illumination of the uvLA-mV DNA with 365 nm UV light showed a 44% recovery of expression. As expected, negligible activation of expression of 1.36% vs NH_2_-DNA was observed from the uvLA-mV DNA without illumination (*p*-value = 0.005) and no significant change was observed following blue light illumination for 1 min (0.71% vs NH_2_-DNA, *p*-value = 0.063). Strikingly, irradiation of the bLA-mV with UV light for 3 min only showed a minor increase in fluorescence of 4.98% compared to the no-light control (*p*-value = 0.011) showing only minimal expression under UV irradiation, demonstrating orthogonal control of CFE from the bLA- and uvLA-mV DNAs. We then tested to see if a graded output could be generated from the bLA-mV DNA by irradiation for less time and lower power (Supporting Figure S20). We saw that just 10 s of illumination with blue light produced 63% activity vs the NH_2_-DNA, with longer times at this power only giving marginal (nonsignificant) increase up to 70% with 1 min. By decreasing the power and illuminating for 10 s, a gradual response was seen, giving rise to 3 or 21% activation vs NH_2_-DNA, respectively.

### Development of an Orthogonal, Dual-Wavelength Light-Activated Cell-Free AND Gate

As we saw only minimal mV expression activation from the bLA–DNA under UV irradiation, we wanted to investigate whether our bLA–DNA, together with the uvLA–DNA, was suitable for orthogonally activating different DNA parts of a cell-free logic gate with two different wavelengths to enable remote-controlled biocomputing.

**Figure 4:**
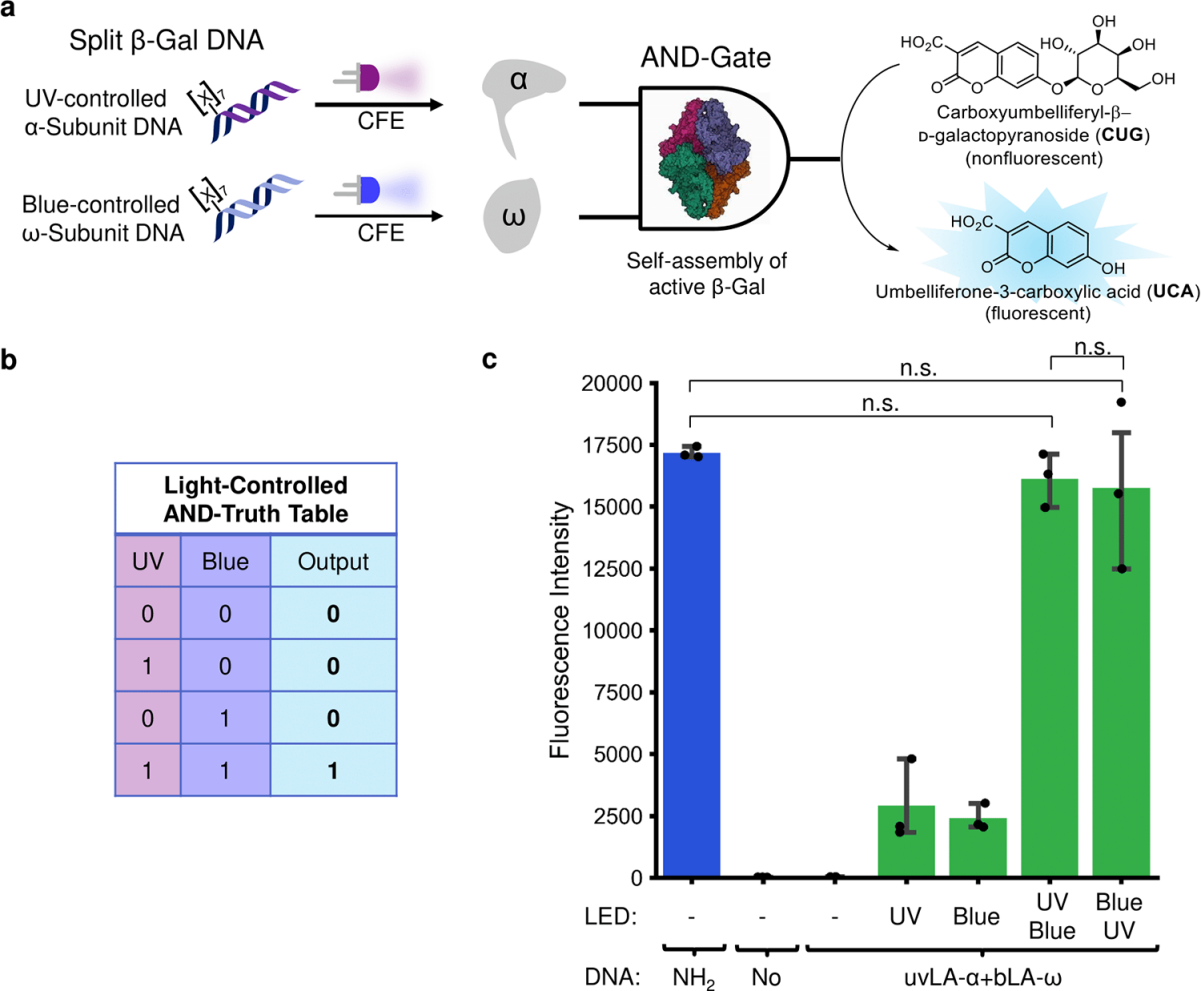
DNA-based AND-gate controlled by two wavelengths of light. (a) Schematic representation of the LA–DNA-based cell-free AND-gate. The two inactive β-galactosidase segments, α and ω, were encoded in a UV or blue LA–DNA part. By placing both light-activatable DNAs inside a CFE system, we generated an AND-gate controlled by light, following the AND-truth table in (b). The output was fluorescence from the enzymatic hydrolysis product umbelliferone-3-carboxylic acid (UCA). (b) Light-controlled AND Truth Table. In the absence of light, no output should be detected. With either UV or blue light only, no output should be detected either, but with both wavelengths of light applied, there is output. (c) Activity of the light-activated AND gate. Split β-galactosidase activity was only reconstituted when both UV and blue light were applied (in either order). Protein Image was reproduced from the RCSB PDB (RCSB.org),^72^ PDB ID: 1DP0,^73,74^ under a CC0 1.0 Universal (CC0 1.0) Public domain dedication licence. *n* = 3. n.s.—nonsignificant (*p*-value > 0.05).

To generate this cell-free logic gate, we chose a split enzyme assay based on the reporter enzyme β-galactosidase (β-Gal). β-Gal was one of the first split enzymes reported.^69^ Through splitting at a loop region, two inactive protein parts (labeled α and ω) were formed, which were able to spontaneously self-assemble in solution and reconstitute enzymatic activity.^70^ We cloned the α and ω subunits from the *lacZ* gene, encoding full-length β-Gal, into the PURExpress control template using homologous recombination (Supporting Figure S21). From this, we generated the linear, blue-activatable ω-subunit DNA by PCR using the bLA-T7 primer, as well as the linear, UV-activatable α-subunit DNA by PCR using the uvLA-T7 primer. Similarly, the amine-only DNAs were prepared as a positive control for 100% photocleavage. Using these parts, a remote-controlled cell-free AND-gate was constructed, using UV and blue light as orthogonal inputs (Figure 4a,b and Supporting Figures S22–S24). Reconstitution of activity was measured using the enzymatic hydrolysis of nonfluorescent carboxyumbelliferyl-β-d-galactopyranoside (CUG) to the fluorescent umbelliferone-3-carboxylic acid (UCA).

We expressed the two DNA parts in a CFE system, with no irradiation, UV, blue, or both UV and blue irradiation, incubated for 4 h at 37 °C and then measured the relative amount of enzyme produced, through the addition of CUG and measuring the development of fluorescence of UCA over time. Initially, we used the UV-activatable primer employed for mV above (Figure 4) (prepared from the UV-photocleavable biotin obtained from Click-Chemistry-Tools), to cage the α-subunit DNA (Supporting Figure S25). Over 1 h of incubation, where the NH_2_-DNA control sample has started to reach a plateau, the system showed a tight OFF-state in the absence of light and presence of UV light only (0% activity after 1 h of incubation with CUG), and a slightly elevated OFF-state upon irradiation with blue light (21% of the maximum output compared to the NH_2_-control). The sample illuminated with both wavelengths only reached 67% of the maximum output after 1 h of incubation; however, the sample illuminated with blue light was at 21% of the maximum output. To better quantify the behavior of the AND-gate under different illumination conditions, the fluorescence of each sample at the timepoint of half the maximum enzymatic output from the control NH_2_-DNA parts was compared (Supporting Figure S25, 10 min, orange line. Supporting Figure S26), where the product formation was at steady state and was thus linear to enzyme concentration (as indicated by the linear slope).^71^ We observed very tight caging in the absence of any irradiation (0.16% activity vs NH_2_-control) as well as after illumination with UV light only (0% activity). Upon illumination with blue light, 9% activity was observed. Illumination with both wavelengths recovered expression to 29% compared to the NH_2_-DNA control at this timepoint. Thus, the data showed an AND-gated response of the designed system but sadly did not reach high activity after illumination with both wavelengths.

To improve on this, we tested a different UV-activatable molecule we have previously characterized (UV-photocleavable biotin from AmberGen, Supporting Figure S13).^42^ We expressed the DNA parts in CFE under different inputs of light and measured the enzymatic production of UCA (Figure 4c and Supporting Figure S27), and again looked at the fluorescence of each sample at the timepoint of half the maximum enzymatic output from the control NH_2_-DNA parts (Supporting Figure S27, 10 min, orange line). In the absence of light, no fluorescence was observed even after 1 h of incubation (99.9% OFF state). In the presence of either UV or blue light alone, UCA was only produced to 14% (UV) and 17% (blue) of that produced by the control NH_2_-DNA parts. Following illumination with both wavelengths in either order, almost full reconstitution of enzymatic activity was observed compared to the amine control, with 94% for blue then UV (*p*-value = 0.180) and 92% for UV then blue (*p*-value = 0.503). Using a calibration curve of full-length β-Gal with CUG (Supporting Figure S28), we quantified the amount of β-Gal produced (in units) from these experiments (Supporting Table S4). These results demonstrate, to our knowledge, the first description of a remote-controlled cell-free expression logic gate activated by two orthogonal wavelengths of light.

### Control and Spatiotemporal Patterning of Cell-Free Expression Inside Synthetic Cells

Synthetic cells, compartments that demonstrate a minimal cellular functionality, have shown great promise in biology, biotechnology, and medicine.^75^ However, these require control mechanisms for future applications, especially using remote stimuli that can simply cross the compartment surface.^33^ We wanted to test if our new bLA–DNA was suitable for the control of different types of synthetic cells and might be applied, with uvLA–DNA, for orthogonal spatiotemporal control of their function. We encapsulated our bLA–DNA and a CFE system within giant unilamellar vesicles (GUVs) and water-in-oil droplets, two of the most widely used types of chassis for synthetic cells.^76^

Initially, we prepared giant unilamellar vesicles (GUVs) containing PURExpress as previously described, using egg phosphatidyl choline (egg-PC) and the inverted emulsion method.^77,78^ We encapsulated bLA-mNeonGreen (mNG) DNA (Supporting Figure S29), an unmodified NH_2_-mNG control DNA, or no DNA with the CFE system inside the vesicles. Texas-Red-Dextran was also included for visualization of the resulting synthetic cells (Supporting Figure S30a). mNeonGreen was chosen for its high brightness, rapid maturation, and better overlap with the filter cubes of our epifluorescence microscope.^79^ After preparation of the GUVs, samples were illuminated, if required, incubated at 37 °C for 5 h, and then imaged by fluorescence microscopy (Supporting Figure S30b–e). As expected, high fluorescence was observed in the synthetic cells that contained the control NH_2_-mNG DNA and no fluorescence was observed in the synthetic cells that contained no DNA. Excitingly, within the synthetic cells containing bLA-mNG DNA in the absence of blue light, we saw no expression of mNG. However, upon illumination for 1 min with 455 nm blue light before incubation, we saw high fluorescence in the synthetic cells, comparable to synthetic cells containing the control NH_2_-mNG DNA.

**Figure 5:**
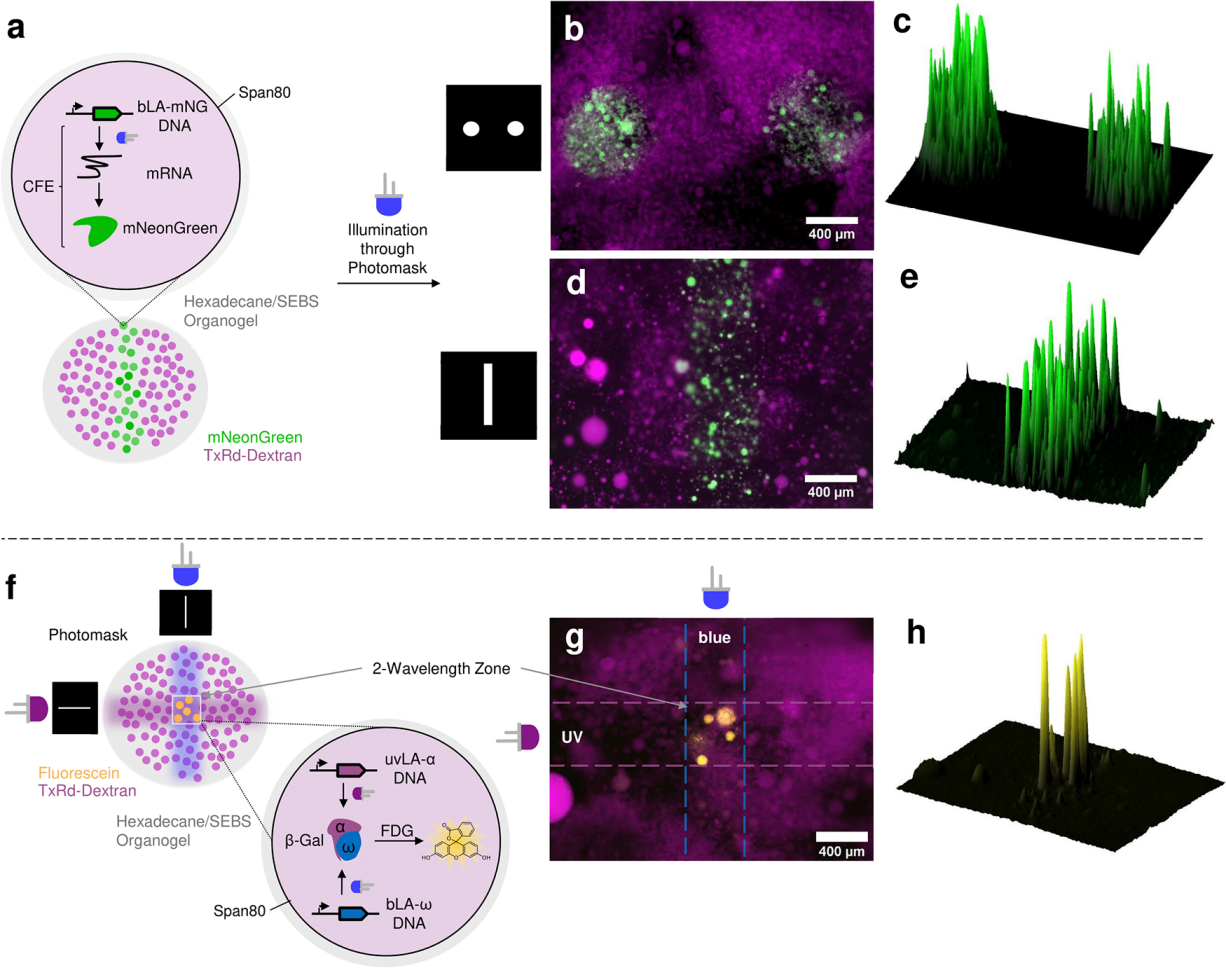
1- and 2-wavelength patterning of immobilized emulsion droplet synthetic cells. (a) Through application of photomasks over organogel-immobilized emulsion droplet synthetic cells, spatial and temporal control over gene expression was achieved. By applying blue light to bLA-mNeonGreen DNA-containing droplets, photopatterns of dots (b, c) and a line (d, e) was achieved. Applying UV and blue light through orthogonal line photomasks onto droplets containing the uvLA-α and bLA-ω DNA parts of split β-gal (f), two-wavelength patterning could be achieved, with fluorescence only observed in the zone where both wavelengths overlap (g, h).

To then allow us to spatially pattern the control of cell-free protein synthesis inside synthetic cells, we prepared emulsion droplets immobilized in an organogel. We emulsified the CFE containing NH_2_- or bLA-mNG DNA and Texas-Red-Dextran using a solution of SEBS polymer^80^ and 2% Span80^81^ in hexadecane by agitation along a PCR rack. A photomask was applied to the droplet-containing organogel and illuminated with blue light prior to incubation for 4 h (Figure 5a). Using this approach, we were able to pattern simple shapes such as dots and lines (Figure 5b–e), whereas the unmodified DNA was expressed in all droplets (Supporting Figure S31), demonstrating the ability to control synthetic cells spatially and temporally using blue light and our bLA–DNA.

Encouraged by these results, we then aimed to spatiotemporally control our orthogonal blue and UV light-activated logic gate within these synthetic cells, to demonstrate remote-controlled, patterned biocomputing (Figure 5f). For this, we encapsulated the CFE system, uvLA-α (synthesized using the Click-Chemistry-Tools UV-photocleavable biotin) and bLA-ω DNA with fluorescein-di-β-d-galactopyranoside (FDG), another common substrate for β-galactosidase, chosen for its improved sensitivity, higher brightness of the hydrolysis product fluorescein, and better overlap with the filter cubes of our microscope. We then applied the line photomask horizontally and illuminated with UV, before turning the line photomask 90° and illuminating with blue light, followed by incubation for 3 h at 37 °C. At the intersection of both illumination lines, a two-wavelength zone of activation was formed, only within which high fluorescence was observed (Figure 5g,h and Supporting Figure S32). This indicated that only in this zone the orthogonal DNA parts were activated, producing the enzyme and then fluorescein. When using NH_2_-DNA, no pattern was observed (Supporting Figure S33). This is, to our knowledge, the first report of two-wavelength spatial control of gene expression and biocomputing inside synthetic cells.

## Conclusions

We have developed a new, blue light-activatable photocage for controlling DNA templates. This blue light-activatable DNA (bLA–DNA) was used to control cell-free protein synthesis in bulk and within synthetic cells, and showed a very tight off-state in the absence of light and rapid photouncaging upon illumination with a blue LED. Due to the orthogonality of this bLA–DNA from our previous UV light-activated DNA, we also generated the first dual-wavelength remote-controlled, cell-free expression AND-gate based on split β-galactosidase, which we applied to spatiotemporally pattern synthetic cells. This technology will be an important addition to the toolkit of cell-free biology as it is independent of the encoded gene and does not require extra biochemical components to function. In the future, this approach might be applied to other commonly employed promoters, such as sigma70 or CMV. Overall, construction of orthogonal and visible light-activated DNA parts will open up applications in cell-free biocomputing from unpicking biological pathways in minimal systems to use for communication of synthetic cells with living systems in biology and medicine.

## Data Availability

All of the data generated in this study are available within the article, the Supporting Information, and figures. Source data are available at https://doi.org/10.5281/zenodo.7808800.

## Supporting Information Available

Synthetic procedures for all compounds, NMR spectra, oligonucleotide mass spectra, HPLC traces, and additional supporting data and procedures (PDF)
