# Supplementary material for: Orthogonal Light-Activated DNA for Patterned Biocomputing within Synthetic Cells
Source: J Am Chem Soc. 2023 Apr 26;145(17):9471–80. doi: 10.1021/jacs.3c02350 (PMC10161232; doi:10.1021/jacs.3c02350)
Supplement: Supplementary file 1 — ja3c02350_si_001.pdf [file ja3c02350_si_001.pdf]

Supplementary Information

Title: Orthogonal light-activated DNA for patterned biocomputing within synthetic cells

Authors: Denis Hartmann[1], Razia Chowdhry[1], Jefferson M. Smith[1], Michael J. Booth[1,2]*

Affiliations:

[1]Department of Chemistry, University of Oxford, Mansfield Road, OX1 3TA, Oxford, UK.

[2]Department of Chemistry, University College London, 20 Gordon Street, WC1H 0AJ, London UK.

*Correspondence: m.j.booth@ucl.ac.uk

Contents

Synthesis 6

General Synthesis 6

7-(Diethylamino)-4-(hydroxymethyl)-2H-chromen-2-one 1 7

4-(((*tert*-Butyldimethylsilyl)oxy)methyl)-7-(diethylamino)-2H-chromen-2-one 2 8

3-Bromo-4-(((*tert*-butyldimethylsilyl)oxy)methyl)-7-(diethylamino)-2H-chromen-2-one 3 9

tert-Butyl-(*E*)-3-(4-(((tert-butyldimethylsilyl)oxy)methyl)-7-(diethylamino)-2-oxo-2H-chromen-3-yl)acrylate 4 10

tert-Butyl-(*E*)-(2-(2-(2-(3-(4-(((*tert*-butyldimethylsilyl)oxy)methyl)-7-(diethylamino)-2-oxo-2H-chromen-3-yl)acrylamido)ethoxy)ethoxy)ethyl)carbamate 5a 11

tert-Butyl-(*E*)-(2-(2-(2-(3-(7-(diethylamino)-4-(hydroxymethyl)-2-oxo-2H-chromen-3-yl)acrylamido)ethoxy)ethoxy)ethyl)carbamate 5b 11

N-(2-(2-(2-((*E*)-3-(4-(((*tert*-Butyldimethylsilyl)oxy)methyl)-7-(diethylamino)-2-oxo-2H-chromen-3-yl)acrylamido)ethoxy)ethoxy)ethyl)-5-((3aS,4S,6aR)-2-oxohexahydro-1H-thieno[3,4-d]imidazol-4-yl)pentanamide 6 13

N-(2-(2-(2-((*E*)-3-(7-(Diethylamino)-4-(hydroxymethyl)-2-oxo-2H-chromen-3-yl)acrylamido)ethoxy)ethoxy)ethyl)-5-((3aS,4S,6aR)-2-oxohexahydro-1H-thieno[3,4-d]imidazol-4-yl)pentanamide 7 14

(7-(Diethylamino)-3-((*E*)-3,14-dioxo-18-((3aS,4S,6aR)-2-oxohexahydro-1H-thieno[3,4-d]imidazol-4-yl)-7,10-dioxa-4,13-diazaoctadec-1-en-1-yl)-2-oxo-2H-chromen-4-yl)methyl (2,5-dioxopyrrolidin-1-yl) carbonate 8a 16

(7-(Diethylamino)-3-((*E*)-3,14-dioxo-18-((3aS,4S,6aR)-2-oxohexahydro-1H-thieno[3,4-d]imidazol-4-yl)-7,10-dioxa-4,13-diazaoctadec-1-en-1-yl)-2-oxo-2H-chromen-4-yl)methyl (perfluorophenyl) carbonate 8b 17

(7-(Diethylamino)-3-((*E*)-3,14-dioxo-18-((3aS,4S,6aR)-2-oxohexahydro-1H-thieno[3,4-d]imidazol-4-yl)-7,10-dioxa-4,13-diazaoctadec-1-en-1-yl)-2-oxo-2H-chromen-4-yl)methyl (4-nitrophenyl) carbonate 8c 18

tert-Butyl (2-(2-(2-aminoethoxy)ethoxy)ethyl)carbamate S1 19

Biotin-NHS S2 19

(7-(Diethylamino)-3-((*E*)-3,14-dioxo-18-((3aS,4S,6aR)-2-oxohexahydro-1H-thieno[3,4-d]imidazol-4-yl)-7,10-dioxa-4,13-diazaoctadec-1-en-1-yl)-2-oxo-2H-chromen-4-yl)methyl prop-2-yn-1-ylcarbamate S3 20

Photochemistry 21

Photocleavage of 8b and 8c 21

Photocleavage of Propargyl-Coumarin S3 22

Fluorescence of PFP-coumarin 8b and propargyl-coumarin S3 24

Oligonucleotide sequences 26

General 26

Amine-modified T7 sequences: 26

PCR Primers 26
DNA Sequences of Genes: 27
Chemical Biology Methods 34
General Chemical Biology Methods 34
Data Analysis and Plotting 34
Polyacrylamide Gel Electrophoresis (PAGE) 34
Agarose Gel Electrophoresis (AGE) 34
Preparation of UV light-activatable T7 Primer (uvLA-T7) 35
Preparation of blue light-activatable-T7 Primer (bLA-T7) 35
Annealing of Broccoli-Construct 36
Plasmid Digestion 36
Preparation of LA-DNA Templates using PCR 36
Streptavidin binding onto T7-modified genes 37
Illumination of Samples 37
Transcription of Broccoli Aptamer 37
Transcription of mVenus, α- and ω-Subunit mRNA 37
Cell-Free Protein Synthesis 38
Cloning of β-Galactosidase within the PURExpress control template 38
Generation of Split β-Galactosidase 39
Preparation of β-Galactosidase Calibration Curve 41
Preparation of Synthetic Cells from Giant Unilamellar Vesicles 42
Preparation of Synthetic Cells from Emulsion Droplets 42
Reactions of active esters with amino-modified DNA 43
Reaction of Amino-modified DNA with 8a 43
Reaction of 7-amino-DNA with PFP-Coumarin 8b in 100 mM $NaHCO_3$: 44
HPLC analysis of reaction conditions of 7-amine-T7 with PFP-coumarin: 45
Comparison of PFP- and pNP-Coumarin reactions: 46
HPLC Traces of light-activatable Primers 47
UV-activatable T7 Primer (Click-Chemistry-Tools) 47
UV-activatable T7 Primer (AmberGen) 48
Blue light-activatable Primer bLA-T7 49
Supplementary Data 50
PAGE of amino-T7 and bLA-T7 50
Expression of Broccoli-aptamer from annealed DNA Templates 51
AGE of bLA-mV DNA at different Illumination Times 52
UV-Visible Absorbance of LA-T7 primers and spectra of the LEDs employed 53

In Vitro Transcription of mVenus 55
Light Titration for the expression of bLA-mV DNA in CFE 56
AGE of split β-Galactosidase Vectors and Inserts 57
AGE of α- and ω-subunit DNA 58
In vitro Transcription of Alpha- and Omega-Subunits of β-Galactosidase 59
In vitro transcription/translation of split β-Galactosidase 61
β-Galactosidase Calibration Curve 64
Quantification of produced β-Galactosidase in Supplementary Figure 27 65
AGE of NH_2- and bLA-mNG DNA 66
Control of Gene Expression inside Giant Unilamellar Vesicle-based Synthetic Cells 67
NH_2-mNG Emulsion Droplets 68
Additional Images of two-wavelength-controlled AND-gate inside Emulsion droplets 69
NH_2-α and -ω-containing Emulsion Droplets 70
Oligonucleotide Mass Spectra 71
Oligonucleotide Mass Spectrometry 71
uvLA-T7 (Click-Chemistry-Tools) 72
uvLA-T7 (AmberGen) 74
bLA-T7 76
NMR Spectra 78
7-(diethylamino)-4-(hydroxymethyl)-2H-chromen-2-one 1 78
4-(((*tert*-butyldimethylsilyl)oxy)methyl)-7-(diethylamino)-2H-chromen-2-one 2 79
3-bromo-4-(((*tert*-butyldimethylsilyl)oxy)methyl)-7-(diethylamino)-2H-chromen-2-one 3 80
tert-butyl (*E*)-3-(4-(((*tert*-butyldimethylsilyl)oxy)methyl)-7-(diethylamino)-2-oxo-2H-chromen-3-yl)acrylate 4 81
tert-butyl-(*E*)-(2-(2-(2-(3-(4-(((tert-butyldimethylsilyl)oxy)methyl)-7-(diethylamino)-2-oxo-2H-chromen-3-yl)acrylamido)ethoxy)ethoxy)ethyl)carbamate 5a 82
tert-butyl (E)-(2-(2-(2-(3-(7-(diethylamino)-4-(hydroxymethyl)-2-oxo-2H-chromen-3-yl)acrylamido)ethoxy)ethoxy)ethyl)carbamate 5b 84
N-(2-(2-(2-((E)-3-(4-(((tert-butyldimethylsilyl)oxy)methyl)-7-(diethylamino)-2-oxo-2H-chromen-3-yl)acrylamido)ethoxy)ethoxy)ethyl)-5-((3aS,4S,6aR)-2-oxohexahydro-1H-thieno[3,4-d]imidazol-4-yl)pentanamide 6 86
N-(2-(2-(2-((E)-3-(7-(diethylamino)-4-(hydroxymethyl)-2-oxo-2H-chromen-3-yl)acrylamido)ethoxy)ethoxy)ethyl)-5-((3aS,4S,6aR)-2-oxohexahydro-1H-thieno[3,4-d]imidazol-4-yl)pentanamide 7 88
(7-(diethylamino)-3-((E)-3,14-dioxo-18-((3aS,4S,6aR)-2-oxohexahydro-1H-thieno[3,4-d]imidazol-4-yl)-7,10-dioxa-4,13-diazaoctadec-1-en-1-yl)-2-oxo-2H-chromen-4-yl)methyl (2,5-dioxopyrrolidin-1-yl) carbonate 8a 90

(7-(diethylamino)-3-((E)-3,14-dioxo-18-((3aS,4S,6aR)-2-oxohexahydro-1H-thieno[3,4-d]imidazol-4-yl)-7,10-dioxa-4,13-diazaoctadec-1-en-1-yl)-2-oxo-2H-chromen-4-yl)methyl (perfluorophenyl) carbonate 8b 92

(7-(diethylamino)-3-((E)-3,14-dioxo-18-((3aS,4S,6aR)-2-oxohexahydro-1H-thieno[3,4-d]imidazol-4-yl)-7,10-dioxa-4,13-diazaoctadec-1-en-1-yl)-2-oxo-2H-chromen-4-yl)methyl (4-nitrophenyl) carbonate 8c 95

tert-butyl (2-(2-(2-aminoethoxy)ethoxy)ethyl)carbamate S1 97

Biotin-NHS S2 98

(7-(diethylamino)-3-((E)-3,14-dioxo-18-((3aS,4S,6aR)-2-oxohexahydro-1H-thieno[3,4-d]imidazol-4-yl)-7,10-dioxa-4,13-diazaoctadec-1-en-1-yl)-2-oxo-2H-chromen-4-yl)methyl prop-2-yn-1-ylcarbamate S3 99

References 101

Synthesis

General Synthesis

Reagents were purchased from commercial sources (Merck, Acros Organics, Fluorochem and Alfa Aesar) and used without further purification. Solvents were used as supplied (analytical/HPLC-grade from Fisher or Sigma-Aldrich) or if dry solvents were required, taken from a solvent drying system (MBraun MB-SPS-5-Bench Top) under nitrogen atmosphere (H_2O content < 20 ppm as determined by Karl Fischer titration). Petroleum ether (PE) over a boiling point range of 40–60 °C was used. Eluent mixtures are reported in volume:volume or %vol. Column chromatography was carried out using Merck Geduran Silica Gel 60 or VWR Silica Gel 40-63 μm under N_2 pressure. Small molecule size exclusion chromatography (SEC) was carried out using Bio-Beads S-X3, 200–400 mesh (BioRad). TLC was carried out on Merck silica gel 60 F254 Al plates. NMR spectroscopy measurements were recorded using a Bruker AVII400, AVII500 or AVIII600 instrument and peaks were referenced to the residual solvent peak. Mass Spectrometry (MS) measurements were carried out on Agilent single quadrupole or Waters LTC (TOF) Mass Spectrometers**.** Liquid Chromatography Mass Spectrometry (LC-MS) was performed on a Waters LTC (TOF) MS equipped with a Waters Acquity UPLC. High Performance Liquid Chromatography (HPLC) was carried out on an Agilent Technologies 1260 Infinity Machine equipped with an Agilent Polaris Amide-C18 column or a Supelco Discovery® BIO Wide Pore C18 column. Infrared Spectra (IR) were recorded on a Bruker Tensor 27 FT-IR and classified as strong (s), medium (m), weak (w) and broad (b). UV/Visible light absorption measurements were performed on a Carys UVBio 50 Spectrometer in 1.4 mL quartz cuvettes (ThorLabs).

7-(Diethylamino)-4-(hydroxymethyl)-2H-chromen-2-one 1

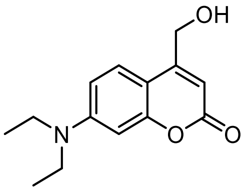

Following a literature procedure:[1] To a solution of 7-(diethylamino)-4-methyl-2H-chromen-2-one (5 g, 21.62 mmol) in DMF (25 mL), DMF–DMA (5.74 mL, 43.24 mmol, 2 eq.) was added and the reaction mixture was heated to reflux for 14 hours. Subsequently, conc. $NaHCO_3$ (25 mL) and CH_2Cl_2 (50 mL) are added. The organic layer was separated and the aqueous layer extracted with CH_2Cl_2 (2x 50 mL). The combined organic layers were dried ($MgSO_4$), filtered and the solvent removed in vacuo. The resulting residue was dissolved in 1:1 THF/H_2O (65 mL), and $NaIO_4$ (13.86 g, 64.85 mmol, 3 eq.) was added. After stirring for 2 hours at ambient temperature the precipitate was filtered off and washed with EtOAc. The solvent volume was reduced in vacuo and conc. $NaHCO_3$ solution (50 mL) was added. The organic layer was separated and the aqueous layer was extracted with CH_2Cl_2 (2x 50 mL). The combined organic layers were dried ($MgSO_4$), filtered and the solvent was removed in vacuo. Subsequently, the residue was dissolved in THF (30 mL), cooled to 0 °C and $NaBH_4$ (1.64 g, 43 mmol, 2 equiv. based on starting coumarin) was added. After stirring for 2 hours at ambient temperature, MeOH was added (5 mL), stirred for 30 minutes and then conc. $NaHCO_3$ solution was added slowly. The organic layer was separated and the aqueous layer was extracted with CH_2Cl_2 (2x 50 mL). The combined organic layers were dried ($MgSO_4$), filtered and the solvent was removed under reduced pressure. Purification by flash column chromatography (SiO_2, EtOAc/PE_{40-60}, 1:1) gave the desired compound as a yellow solid (2.48 g, 10 mmol, 48% over 3 steps). **^{1}H-NMR (400 MHz, $CDCl_3$)** δ/ppm 7.32 (d, J = 9.0 Hz, 1H, C-H_{Ar}), 6.56 (dd, J = 9.0, 2.6 Hz, 1H, C-H_{Ar}), 6.51 (d, J = 2.6 Hz, 1H, C-H_{Ar}), 6.25 (t, J = 1.4 Hz, 1H, C-H), 4.83 (dd, J = 6.2, 1.4 Hz, 2H, CH_2), 3.41 (q, J = 7.1 Hz, 4H, CH_2), 1.97 (t, J = 6.2 Hz, 1H, O-H), 1.20 (t, J = 7.1 Hz, 6H CH_3). **MS (ESI$^+$)** found 270.0 [M+H$^+$]. Data in accordance with literature.[1]

4-(((*tert*-Butyldimethylsilyl)oxy)methyl)-7-(diethylamino)-2H-chromen-2-one 2

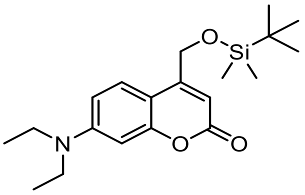

Following a literature procedure:[2] To a 250 mL round-bottom flask equipped with a stirrer bar, coumarin-alcohol **1** (2 g, 8.0 mmol) and TBDMS-Cl (1.46 g, 9.6 mmol) were added and dissolved in CH_2Cl_2 (100 mL). To this, DMAP (328 mg, 2.44 mmol) and NEt_3 (0.84 mL, 12 mmol) were added and the reaction stirred for 3 hours. The reaction was quenched by addition of sat. $NaHCO_3$ (50 mL). The aqueous layer was extracted with EtOAc (2x 25 mL), the organic layers combined, dried ($MgSO_4$), filtered and reduced *in vacuo*. The crude residue was then purified by flash column chromatography (SiO_2, 9:1-3:1 Hexane:EtOAc) to yield a pale yellow solid as the desired compound (2.635 g, 7.43 mmol, 98%). **^{1}H-NMR (400 MHz, $CDCl_3$)** δ/ppm 7.24 (s, 1H, C-H_{Ar}), 6.54 (dd, *J* = 8.9, 2.6 Hz, 1H, C-H_{Ar}), 6.51 (d, *J* = 2.6 Hz, 1H, C-H_{Ar}), 6.28 (t, *J* = 1.5 Hz, 1H, C-H), 4.81 (d, *J* = 1.5 Hz, 2H, CH_2), 3.41 (q, *J* = 7.1 Hz, 4H, CH_2), 1.20 (t, *J* = 7.1 Hz, 6H, CH_3), 0.96 (s, 9H, CH_3), 0.14 (s, 6H, CH_3). **MS (ESI^+)** found 362.2 [$M+H^+$]. Data in accordance with literature.[2]

3-Bromo-4-(((*tert*-butyldimethylsilyl)oxy)methyl)-7-(diethylamino)-2H-chromen-2-one 3

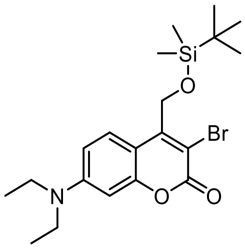

Following a literature procedure:[2] TBDMS-Coumarin **2** (1 g, 2.77 mmol) and N-bromosuccinimide (0.55 g, 3.04 mmol) were added to a 50 mL round-bottom flask equipped with a stirrer bar and dissolved in MeCN (9 mL). To this, NH_4OAc (21 mg, 0.27 mmol) was added and the reaction stirred for 1 hour, during which time the reaction turned from yellow to orange. The reaction was then quenched with water (20 mL), the aqueous layer extracted with EtOAc (2x 20 mL), the organic layers combined, dried ($MgSO_4$), filtered and reduced *in vacuo*. The resulting residue was then subjected to flash column chromatography (SiO_2, 8:1 Hexane:EtOAc) to yield a bright yellow solid as the desired product (1.22 g, 2.77 mmol, 100%). **^{1}H-NMR (400 MHz, $CDCl_3$)** δ/ppm 7.76 (d, *J* = 9.2 Hz, 1H, C-H_{Ar}), 6.61 (dd, *J* = 9.2, 2.6 Hz, 1H, C-H_{Ar}), 6.48 (d, *J* = 2.6 Hz, 1H, C-H_{Ar}), 5.00 (s, 2H, CH_2), 3.42 (q, *J* = 7.1 Hz, 4H, CH_2), 1.21 (t, *J* = 7.1 Hz, 6H, CH_3), 0.90 (s, 9H CH_3), 0.14 (s, 6H, CH_3). **MS (ESI$^+$)** found 440.2 [M+H$^+$]. Data in accordance with literature.[2]

***tert*-Butyl-(*E*)-3-(4-(((tert-butyldimethylsilyl)oxy)methyl)-7-(diethylamino)-2-oxo-2H-chromen-3-yl)acrylate 4**

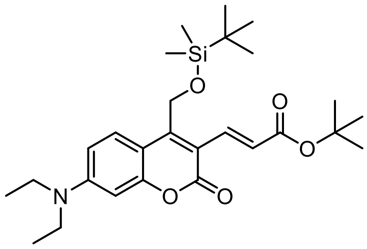

Following a literature procedure:[2] To a solution of Bromo-TBDMS-Coumarin **3** (2 g, 4.56 mmol) in DMF (44 mL) in a Schlenk flask was added tert-butyl acrylate (2.2 mL, 14.96 mmol). To this, LiCl (328 mg, 7.72 mmol), $NaHCO_3$ (1.14 g, 13.6 mmol) and tetrabutylammonium chloride (1.39 g, 5 mmol) were added and the solution degassed through purging with nitrogen for 30 minutes. Then $Pd(OAc)_2$ (52 mg, 0.2 mmol) was added under a gentle stream of nitrogen and the flask sealed under positive pressure. The reaction was then heated to 110 °C for 60 minutes, during which the reaction turned dark orange first, and then blackish green. The reaction was then cooled to room temperature, quenched with water (40 mL), the aqueous layer extracted with EtOAc (3x 30 mL), the organic layers combined, washed with brine (30 mL), dried ($MgSO_4$), filtered and reduced *in vacuo*. The crude mixture was then purified by flash column chromatography (SiO_2, 9:1 PE:EtOAc) to yield the desired compound as an orange-yellow solid (1.9 g, 3.9 mmol, 85%). **^{1}H-NMR (400 MHz, $CDCl_3$)** δ/ppm 7.75 (d, *J* = 15.7 Hz, 1H, C-H), 7.66 (d, *J* = 9.2 Hz, 1H, C-H_{Ar}), 6.99 (d, *J* = 15.6 Hz, 1H, C-H), 6.62 (dd, *J* = 9.2, 2.7 Hz, 1H, C-H_{Ar}), 6.48 (d, *J* = 2.6 Hz, 1H, C-H_{Ar}), 4.91 (s, 2H, CH_2), 3.43 (q, *J* = 7.1 Hz, 4H, CH_2), 1.52 (s, 9H, CH_3), 1.22 (t, *J* = 7.1 Hz, 6H, CH_3), 0.90 (s, 9H, CH_3), 0.15 (s, 6H, CH_3). **MS (ESI^+)** found 510.0 [$M+Na^+$]. Data in accordance with literature.[2]

***tert*-Butyl-(*E*)-(2-(2-(2-(3-(4-(((*tert*-butyldimethylsilyl)oxy)methyl)-7-(diethylamino)-2-oxo-2H-chromen-3-yl)acrylamido)ethoxy)ethoxy)ethyl)carbamate 5a**

and

***tert*-Butyl-(*E*)-(2-(2-(2-(3-(7-(diethylamino)-4-(hydroxymethyl)-2-oxo-2H-chromen-3-yl)acrylamido)ethoxy)ethoxy)ethyl)carbamate 5b**

Coumarin **4** (146.8 mg, 0.301 mmol) was dissolved in 6 mL 1:1 CH_2Cl_2:TFA and left stirring for 30 minutes, until TLC showed full consumption of starting material. The solvent was then removed *in vacuo*, the resulting residue resuspended in CH_2Cl_2 and the solvent removed *in vacuo* again to remove residual TFA. This process was repeated two times. The resulting orange solid was then resuspended in dry MeCN (10 mL). To this was added *N*-Boc-Diamine **S1** (187 mg, 0.753 mmol, 2.5 eq.) and EDC·HCl (144 mg, 0.753 mmol, 2.5 eq.) and the reaction left stirring overnight. The solvent was then removed *in vacuo* and the crude reaction mixture was purified using flash column chromatography (3:1 EtOAc:PE – 1:0 EtOAc:PE – 1:0 CH_2Cl_2 – 5% MeOH in CH_2Cl_2) to yield TBDMS-Protected compound **5a** (104.3 mg, 158 mmol, 52%) as well as deprotected compound **5b** (72.9 mg, 0.133 mmol, 44%) as yellow solids with a combined total yield of 96%.

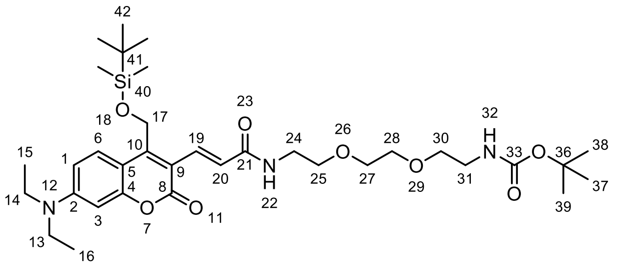

R*f*. (SiO2, 3:1 EtOAc:PE) 0.30. **MS (ESI$^+$)** found 662.3 [M+H$^+$]. **^{1}H-NMR (600 MHz, $CDCl_3$)** δ/ppm 7.84 (d, *J* = 15.1 Hz, 1H, 19, C-H), 7.70 (d, *J* = 9.2 Hz, 1H, 6, CH_{Ar}), 7.24 (d, *J* = 15.1 Hz, 1H, 20, C-H), 6.62 (dd, *J* = 9.2, 2.6 Hz, 1H, 1, CH_{Ar}), 6.47 (d, *J* = 2.6 Hz, 1H, 3, CH_{Ar}), 6.23 (s, 1H, 22, N-H), 5.08 (s, 1H, 32, N-H), 4.96 (s, 2H, 17, CH_2), 3.63 (s, 4H, 27, 28, CH_2), 3.63 – 3.52 (m, 2H, 25, CH_2), 3.43 (q, *J* = 7.1 Hz, 4H, 13, 14, CH_2), 3.35 (s, 2H, 31, CH_2), 1.43 (s, 9H, 44, 45, 46, CH_3), 1.22 (t, *J* = 7.1 Hz, 6H, 15, 16, CH_3), 0.90 (s, 9H, 40, 41, 42, CH_3), 0.17 (s, CH_3, 38, 39, 6H). **^{13}C-NMR (151 MHz, $CDCl_3$)** δ/ppm 166.78 (21), 161.03 (8), 156.22 (33), 155.87 (4), 151.75 (10), 151.11 (2), 132.10 (19), 127.82 (6), 124.97 (20), 113.29 (9), 109.32 (1), 108.62 (5), 97.16 (3), 79.35 (43), 70.54 and 70.43 and 70.30 and 70.16 (4C, 25, 27, 28, 30), 57.73 (17), 44.98 (2C, 13, 14), 40.44 (31), 39.62 (24), 28.57 (3C, 44, 45, 46), 25.97 (3C, 40, 41, 42), 18.39 (37), 12.67 (2C, 15, 16), -4.97 (2C, 38, 39). **HRMS (ESI$^+$)** found 662.3831 (theoretical m/z = 662.3831 $[C_{34}H_{56}O_8N_3Si]^+$). **IR** (film, $CDCl_3$/cm^{-1}) 3328 (wb, N-H), 2927 (m), 1711 (s, C=O), 1602 (s, C=O), 1510 (s), 1359 (m), 1091 (m).

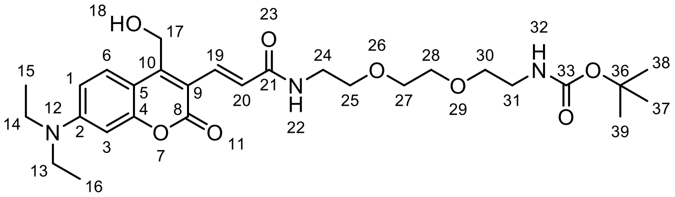

R*f* (SiO$_2$, 5% MeOH/CH$_2$Cl$_2$) 0.36. **^{1}H-NMR (500 MHz, CDCl$_3$)** δ/ppm 7.82 (d, *J* = 15.2 Hz, 1H, 19, C-H), 7.73 (d, *J* = 9.2 Hz, 1H, 6, C-H$_{Ar}$), 7.20 (d, *J* = 15.2 Hz, 1H, 20, C-H), 6.61 (dd, *J* = 9.2, 2.7 Hz, 1H, 1, C-H$_{Ar}$), 6.52 (s, 1H, 32, N-H), 6.44 (d, *J* = 2.6 Hz, 1H, 3, C-H$_{Ar}$), 5.16 (s, 1H, 22, N-H), 4.94 (s, 1H, 17, CH$_2$), 4.21 (s, 1H, 18, O-H), 3.61 (s, 4H, 27, 28, CH$_2$), 3.61 – 3.49 (m, 4H, 25, 30, CH$_2$), 3.52 (q, *J* = 5.2 Hz, 2H, 31, CH$_2$), 3.41 (q, *J* = 7.1 Hz, 4H, 13, 14, CH$_2$), 3.32 (q, *J* = 6.1, 5.5 Hz, 2H, 24, CH$_2$), 1.41 (s, 9H, 37, 38, 39, CH$_3$), 1.20 (t, *J* = 7.1 Hz, 6H, 15, 16, CH$_3$). **^{13}C-NMR (126 MHz, CDCl$_3$)** δ/ppm 167.35 (21), 161.01 (8), 156.27 (33), 155.91 (4), 152.01 (10), 151.17 (2), 132.35 (19), 127.64 (6), 124.61 (20), 113.31 (9), 109.54 (1), 108.50 (5), 97.15 (3), 79.38 (36), 70.44 and 70.35 and 70.23 and 69.92 (4C, 25, 27, 28, 30), 56.53 (17), 44.98 (2C, 13, 14), 40.40 (24), 39.68 (31), 28.53 (3C, 37, 38, 39), 12.63 (2C, 15, 16). **MS (ESI$^+$)** found 570.2 [M+Na$^+$]. **HRMS (ESI$^+$)** found 570.2786 (theoretical m/z = 570.2786 [C$_{21}$H$_{41}$O$_8$N$_3$^{23}Na]$^+$). **IR** (film, CDCl$_3$/cm^{-1}) 3305 (wb, O-H), 2972 (w C-H$_{Ar}$), 2928 (w, C-H$_{Ar}$), 1704 (s, C=O), 1618 (s, C=O), 1596 (s), 1566 (s) 1514 (m).

N-(2-(2-(2-((*E*)-3-(4-(((*tert*-Butyldimethylsilyl)oxy)methyl)-7-(diethylamino)-2-oxo-2H-chromen-3-yl)acrylamido)ethoxy)ethoxy)ethyl)-5-((3aS,4S,6aR)-2-oxohexahydro-1H-thieno[3,4-d]imidazol-4-yl)pentanamide 6

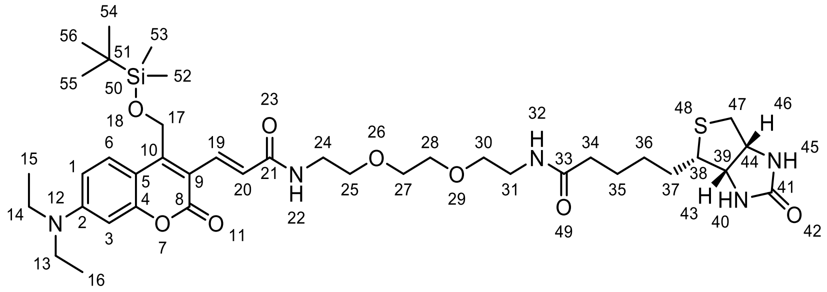

To a solution of TBDMS-protected coumarin **5a** (104 mg, 0.157 mmol) in 2 mL CH_2Cl_2 was added 2 mL of TFA and the reaction mixture stirred for 1 hour at room temperature. The solvent was removed *in vacuo* and the residue redissolved in CH_2Cl_2 and the solvent removed again two times to remove residual TFA. The resulting residue was then dissolved in 2 mL of dry DMF, to which was added dry triethylamine (65 µL, 0.471 mmol, 3 eq.) and then Biotin-NHS was added (75 mg, 1.5 eq.) and the reaction left stirring for 4 hours. The solvent was removed *in vacuo* and the compound was purified using flash chromatography (SiO_2, 10% MeOH/$CHCl_3$) to give compound **6** as a yellow solid (35.3 mg, 0.045 mmol, 29%) as well as compound **7** as a yellow solid (75 mg, 0.111 mmol, 71%).

R_f (SiO_2, 10% MeOH/CH_2Cl_2) 0.48. **^{1}H-NMR (500 MHz, $CDCl_3$)** δ/ppm 7.81 (d, *J* = 15.1 Hz, 1H, 19, C-H), 7.69 (d, *J* = 9.2 Hz, 1H, 6, CH_{Ar}), 7.25 (d, *J* = 14.6 Hz, 2H, 20, C-H, partially covered by $CDCl_3$), 6.91 (t, *J* = 5.4 Hz, 2H, 22, 32, N-H), 6.62 (dd, *J* = 9.2, 2.6 Hz, 1H, 1, CH_{Ar}), 6.45 (d, *J* = 2.7 Hz, 2H, 3, 40, CH_{Ar} and N-H), 5.59 (s, 1H, 45, N-H), 4.94 (s, 2H, 17, CH_2), 4.49 (ddd, *J* = 12.6, 7.7, 4.8 Hz, 1H, 46, C-H), 4.31 (td, *J* = 7.9, 4.3 Hz, 1H, 43, C-H), 3.65 – 3.38 (m, 10H, 24, 25, 27, 28, 30, 31, CH_2), 3.42 (q, *J* = 6.9 Hz, 6H, 13, 14, CH_2), 3.12 (qd, *J* = 7.0, 3.5 Hz, 1H, 38, C-H), 2.87 (dd, *J* = 12.5, 5.0 Hz, 1H, 47, CH_2), 2.72 (d, *J* = 12.4 Hz, 1H, 47, CH_2), 2.22 (t, *J* = 7.4 Hz, 2H, 34, CH_2), 1.87 – 1.56 (m, 4H, 35, 37, CH_2), 1.46 – 1.36 (m, 2H, 36, CH_2), 1.21 (t, *J* = 7.1 Hz, 6H, 15, 16, CH_3), 0.88 (s, 9H, 54, 55, 56, CH_3), 0.15 (s, 6H, 52, 53, CH_3). **^{13}C-NMR (126 MHz, $CDCl_3$)** δ/ppm 173.71 (33), 166.95 (21), 164.11 (41), 161.12 (8), 155.78 (4), 151.88 (10), 151.11 (2), 131.80 (19), 127.82 (6), 125.13 (20), 113.11 (9), 109.39 (1), 108.54 (5), 97.01 (3), 70.29 and 70.17 and 70.10 and 70.05 (4C, 25, 27, 28, 30), 61.89 (39), 60.34 (44), 57.69 (17), 55.68 (38), 44.95 (13, 14), 40.60 (47), 39.60 (24 or 31) 39.18 (24 or 31) 35.97 (34), 28.15 (36), 25.94 (3C, 54, 55, 56), 25.74 and 25.68 (2C, 35, 37), 18.36 (51), 12.64 (2C, 15, 16), -5.01 (2C, 52, 53). **MS (ESI$^+$)** found 788.3 $[M+H]^+$. **HRMS (ESI$^+$)** found 788.4077 (theoretical m/z = 788.4083 $[C_{39}H_{62}N_5O_8SSi]^+$). **IR** (film, $CDCl_3$) 3291 (wb, N-H), 2926 (m, C-H), 1700 (s, C=O), 1601 (s), 1510 (m), 1083 (m)

N-(2-(2-(2-((*E*)-3-(7-(Diethylamino)-4-(hydroxymethyl)-2-oxo-2H-chromen-3-yl)acrylamido)ethoxy)ethoxy)ethyl)-5-((3aS,4S,6aR)-2-oxohexahydro-1H-thieno[3,4-d]imidazol-4-yl)pentanamide 7

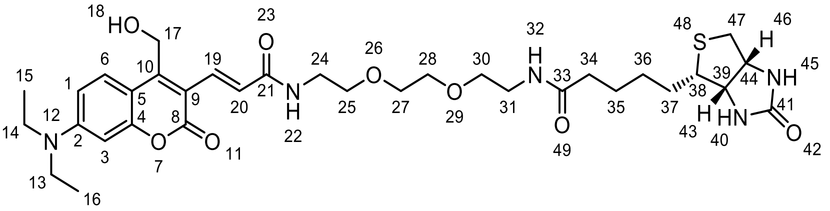

To a solution of OH-coumarin **5b** (40 mg, 0.073 mmol) in CH_2Cl_2 (2 mL) was added TFA (2 mL) and left stirring for 30 minutes, until TLC indicated full deprotection. The solvent was subsequently removed *in vacuo*, the residue resuspended in CH_2Cl_2 and the solvent removed *in vacuo* again. The residue was then dissolved in DMF (3 mL). To this was added Biotin-NHS Ester (37.5 mg, 0.110 mmol) and NEt_3 (60 µL). The reaction was left stirring for 3 hour. The solvent was removed *in vacuo* and the resulting crude mixture purified using flash column chromatography (SiO_2, 5-10% MeOH/CH_2Cl_2) and further purified using flash chromatography (SiO_2, 10% MeOH/$CHCl_3$) to yield a bright yellow solid as the desired compound (46.3 mg, 0.069 mmol, 69% over 2 steps).

Alternatively, to a solution of Coumarin **6** (219 mg, 0.278 mmol) in THF (3 mL) was added a solution of TBAF in THF (1M, 1.6 mL, 6 eq.) and left stirring for 45 minutes. The solvent was subsequently removed, and the resulting residue purified using flash column chromatography (10% MeOH/$CHCl_3$) to yield **7** as a bright yellow solid (169 mg, 250 mmol, 90%).

R_f (SiO_2, 10% MeOH/CH_2Cl_2) 0.29. **^{1}H-NMR (500 MHz, DMSO-d6)** δ/ppm 8.27 (t, *J* = 5.7 Hz, 1H, 22, N-H), 7.83 (t, *J* = 5.7 Hz, 1H, 32, N-H), 7.76 (d, *J* = 9.3 Hz, 1H, 6, CH_{Ar}), 7.65 (d, *J* = 15.4 Hz, 1H, 19, C-H), 7.13 (d, *J* = 15.4 Hz, 1H, 20, C-H), 6.77 (dd, *J* = 9.3, 2.6 Hz, 1H, 1, CH_{Ar}), 6.53 (d, *J* = 2.5 Hz, 1H, 3, CH_{Ar}), 6.41 (bt, *J* = 1.9 Hz, 1H, 40, N-H), 6.35 (bs, 1H, 45, N-H), 5.57 (t, *J* = 5.5 Hz, 1H, 18, O-H), 4.72 (d, *J* = 4.6 Hz, 2H, 17, CH_2), 4.29 (dd, *J* = 7.7, 5.1 Hz, 1H, 46, C-H), 4.14 – 4.08 (m, 1H, 43, C-H), 3.55 – 3.49 (m, 4H, 27, 28, CH_2), 3.46 (skewed q, *J* = 6.8 Hz, 6H, 13, 14, 25, CH_2), 3.40 (t, *J* = 5.9 Hz, 2H, 30, CH_2), 3.33 – 3.28 (m, 2H, 24, CH_2 partially hidden in H_2O peak), 3.18 (q, *J* = 5.8 Hz, 2H, 31, CH_2), 3.08 (ddd, *J* = 8.6, 6.2, 4.4 fHz, 1H, 38, C-H), 2.80 (dd, *J* = 12.5, 5.1 Hz, 1H, 47', CH_2), 2.57 (d, *J* = 12.4 Hz, 1H, 47", CH_2), 2.06 (t, *J* = 7.4 Hz, 2H, 34, CH_2), 1.66 – 1.24 (m, 6H, 35, 36, 37, CH_2 Biotin), 1.13 (t, *J* = 7.0 Hz, 6H, 15, 16, CH_3). **^{13}C-NMR (126 MHz, DMSO-d6)** δ/ppm 172.14 (33), 165.88 (21), 162.70 (41), 159.89 (8), 155.23 (4), 152.41 (10), 150.74 (2), 131.09 (19), 127.96 (6), 125.15 (20), 112.37 (9), 109.38 (1), 107.60 (5), 96.24 (3), 69.56 and 69.53 (2C, 27, 28), 69.18 (25),

69.09 (30), 61.03 (39), 59.18 (44), 55.43 (17), 55.24 (38), 44.10 (13, 14), 39.69 (47), 38.82 (24), 38.44 (31), 35.10 (34), 28.19 (36), 28.04 (37), 25.27 (35), 12.40 (2C, 15, 16). **MS (ESI$^+$)** found 696.2 [M+Na$^+$]. **HRMS (ESI$^+$)** found 696.3036 (theoretical m/z = 696.3038 [$C_{33}H_{47}O_8N_5{}^{23}NaS$]$^+$). **IR** (neat/cm^{-1}) 3284 (sb, O-H), 2924 (w, C-H_{Ar}), 1704 (s, C=O), 1645 (C=O Biotin) 1620 (s, C=O), 1597 (s), 1551 (m), 1515 (m)

(7-(Diethylamino)-3-((*E*)-3,14-dioxo-18-((3aS,4S,6aR)-2-oxohexahydro-1H-thieno[3,4-d]imidazol-4-yl)-7,10-dioxa-4,13-diazaoctadec-1-en-1-yl)-2-oxo-2H-chromen-4-yl)methyl (2,5-dioxopyrrolidin-1-yl) carbonate 8a

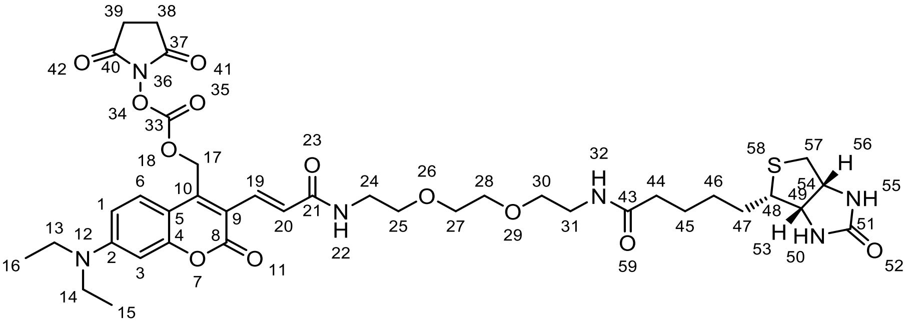

To a solution of coumarin **7** (24 mg, 0.036 mmol) in dry DMSO (0.7 mL) was added *N,N*-disuccinimidyl carbonate (28 mg, 0.108 mmol) and NEt_3 (15 µL, 0.108 mmol) and left stirring for 2 hours, during which the reaction turned dark orange. The crude solution in DMSO was lyophilised to remove the solvent and the residue was purified by flash column chromatography (SiO_2, 6:1 $CHCl_3$:MeOH), the fractions containing the molecule combined, dried, resuspended in CH_2Cl_2 and further purified using size exclusion chromatography (BioBeads SX-3, CH_2Cl_2) to yield the desired compound as a dark orange solid (20 mg, 0.026 mmol, 72%). $\mathbf{R_f}$ (SiO_2, 6:1 $CHCl_3$:MeOH) 0.41. $\mathbf{R_f}$ (SiO_2, 10%MeOH/CH_2Cl_2) 0.56. **^{1}H NMR (500 MHz, $CDCl_3$)** δ/ppm 8.01 (d, *J* = 9.2 Hz, 1H, 6, C-H_{Ar}), 7.76 (d, *J* = 15.6 Hz, 1H, 19, C-H), 7.21 (d, *J* = 15.6 Hz, 1H, 20, C-H), 7.01 (t, *J* = 5.6 Hz, 1H, 22, N-H), 6.82 (t, *J* = 5.7 Hz, 1H, 32, N-H), 6.70 (dd, *J* = 9.2, 2.6 Hz, 1H, 1, C-H_{Ar}), 6.45 (d, *J* = 2.6 Hz, 1H, 3, C-H_{Ar}), 6.35 (s, 1H, 55, N-H), 5.50 (s, 1H, 50, N-H), 5.30 (s, 2H, 17', CH_2), 4.47 (dd, *J* = 7.8, 4.9 Hz, 1H, 56, C-H), 4.33 – 4.26 (m, 1H, 53, C-H), 3.69 – 3.61 (m, 6H, 25, 27, 28, CH_2), 3.61 – 3.53 (m, 4H, 24, 30, CH_2), 3.49 (d, *J* = 5.1 Hz, 0H), 3.49 – 3.36 (m, 2H, 31, CH_2), 3.42 (q, *J* = 7.0 Hz, 4H, 13, 14, CH_2), 3.10 (td, *J* = 7.4, 4.6 Hz, 1H, 48, C-H), 2.87 (dd, *J* = 12.8, 5.0 Hz, 1H, 57', CH_2), 2.79 (s, 4H, 38'', 39'', CH_2), 2.70 (d, *J* = 12.8 Hz, 1H, 57'', CH_2), 2.20 (t, *J* = 7.5 Hz, 2H, 44, CH_2), 1.75 – 1.50 (m, 4H, 45, 47, CH_2), 1.40 (h, *J* = 7.5, 6.8 Hz, 2H, 46, CH_2), 1.21 (t, *J* = 7.1 Hz, 6H, 15, 16, CH_3). **^{13}C NMR (126 MHz, $CDCl_3$)** δ/ppm 173.60 (43), 171.50 (2C, 37, 40), 166.33 (21), 164.06 (51), 160.93 (8), 155.62 (4), 151.29 (2), 142.46 (10), 131.78 (19), 127.59 (6), 127.32 (20), 117.61 (9), 110.00 (1), 108.70 (5), 97.19 (3), 70.58 (17), 70.32 and 70.30 and 70.03 and 69.97 (4C, 25, 27, 28, 30), 61.92 (49), 60.30 (54), 55.69 (48), 45.04 (2C, 13, 14), 40.65 (57), 39.67 (24), 39.22 (31), 36.04 (44), 28.28 (46), 28.16 (47), 25.81 (2C, 38, 39), 25.70 (45), 12.65 (2C, 15, 16). **IR** (film $CDCl_3$/cm^{-1}) 3293.8 (bw), 2951 (w), 2925 (w), 1698 (m), 1655 (m), 1615 (m) 1601 (m), 1569 (m), 1514 (m), 729 (s). **HRMS (ESI$^+$)** found 815.3279 (theoretical m/z = 815.3280 $[C_{38}H_{51}O_{12}N_6S]^+$).

(7-(Diethylamino)-3-((*E*)-3,14-dioxo-18-((3aS,4S,6aR)-2-oxohexahydro-1H-thieno[3,4-d]imidazol-4-yl)-7,10-dioxa-4,13-diazaoctadec-1-en-1-yl)-2-oxo-2H-chromen-4-yl)methyl (perfluorophenyl) carbonate 8b

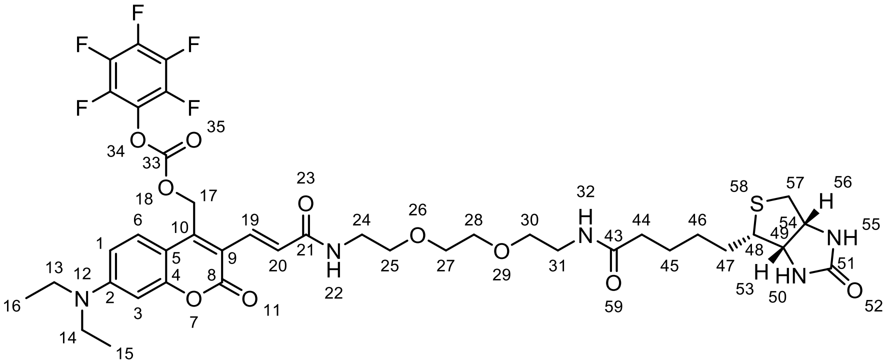

To a solution of coumarin **7** (74 mg, 0.111 mmol) in dry DMSO (2 mL) was added Bis(pentafluorophenyl)carbonate (110 mg, 0.278 mmol) and NEt_3 (39 µL, 0.278 mmol) and left stirring for 2 hours protected from light, during which the reaction turned a dark reddish-orange. The crude solution in DMSO was dried under convection to remove the majority of the solvent and the residue was purified by flash column chromatography (SiO_2, 9:1 CH_2Cl_2:MeOH), the fractions containing the molecule combined, dried, resuspended in CH_2Cl_2 and further purified using size exclusion chromatography (BioBeads SX-3, CH_2Cl_2) to yield the desired compound as a dark orange solid (27 mg, 0.030 mmol, 29%). **R_f**(SiO_2, 10%MeOH/$CHCl_3$) 0.27. **^{1}H-NMR (500 MHz, $CDCl_3$)** δ/ppm 7.80 (d, *J* = 15.1 Hz, 1H, 19), 7.55 (d, *J* = 9.3 Hz, 1H, 6), 7.31 (d, *J* = 15.0 Hz, 1H, 20), 6.91 (t, *J* = 5.4 Hz, 1H, 22), 6.67 (dd, *J* = 9.3, 2.0 Hz, 1H, 3), 6.60 (d, *J* = 5.9 Hz, 1H, 32), 6.50 (d, *J* = 2.5 Hz, 1H, 1), 6.12 (s, 1H, 40), 5.68 (s, 1H, 17), 5.25 (s, 1H, 45), 4.50 (t, *J* = 6.5 Hz, 1H, 46), 4.39 – 4.28 (m, 1H, 43), 3.71 – 3.54 (m, 10H, 24, 25, 27, 28, 30, 31), 3.45 (q, *J* = 7.1 Hz, 6H, 13, 14, 31), 3.13 (td, *J* = 7.4, 4.6 Hz, 1H, 38), 2.89 (dd, *J* = 12.8, 4.9 Hz, 1H, 47'), 2.73 (d, *J* = 12.8 Hz, 1H, 47''), 2.22 (t, *J* = 7.4 Hz, 2H, 34), 1.79 – 1.32 (m, 6H, 35, 36, 37), 1.23 (t, *J* = 7.0 Hz, 6H, 15, 16). **^{13}C-NMR (126 MHz, $CDCl_3$)** δ/ppm 173.49 (33), 166.61 (21), 163.79 (41), 160.42 (8), 155.75 (4), 151.53 (2), 151.15 (50), 144.15 (10), 130.92 (19), 126.64 (6), 126.59 (20), 115.48 (9), 110.01 (3), 107.73 (5), 97.39 (1), 70.33 and 70.21 and 69.99 and 69.95 (4C, 25, 27, 28, 30), 63.14 (17), 61.96 (39), 60.31 (44), 55.55 (38), 45.10 (13, 14), 40.61 (47), 39.73 (24), 39.18 (31), 36.00 (34), 28.19 (2C, 36, 37), 25.62 (35), 12.64 (2C, 15, 16). Pentafluorophenyl-carbons were not observed due to ^{13}C-^{19}F coupling. **^{19}F-NMR (471 MHz, $CDCl_3$) δ/ppm** -152.26 – -152.95 (m, 2F), -156.96 (t, *J* = 21.8 Hz, 1F), -161.66 (td, *J* = 22.7, 5.0 Hz, 2F). **IR** (film $CDCl_3$/cm^{-1}) 3302 (w), 2927 (w), 1701 (s, C=O), 1620 (s), 1600 (s), 1519 (s). **MS (ESI$^+$)** found 906.3 [M+Na$^+$]. **HRMS (ESI$^+$)** found 884.2949 (theoretical m/z = 884.2958 $[C_{40}H_{47}O_{10}N_5F_5S_1]^+$). **UV/Vis** (0.05% DMF in H_2O) λ_{max}/nm (ε/mM^{-1} cm^{-1}) 280 (7.7), 443 (16.1)

(7-(Diethylamino)-3-((*E*)-3,14-dioxo-18-((3aS,4S,6aR)-2-oxohexahydro-1H-thieno[3,4-d]imidazol-4-yl)-7,10-dioxa-4,13-diazaoctadec-1-en-1-yl)-2-oxo-2H-chromen-4-yl)methyl (4-nitrophenyl) carbonate 8c

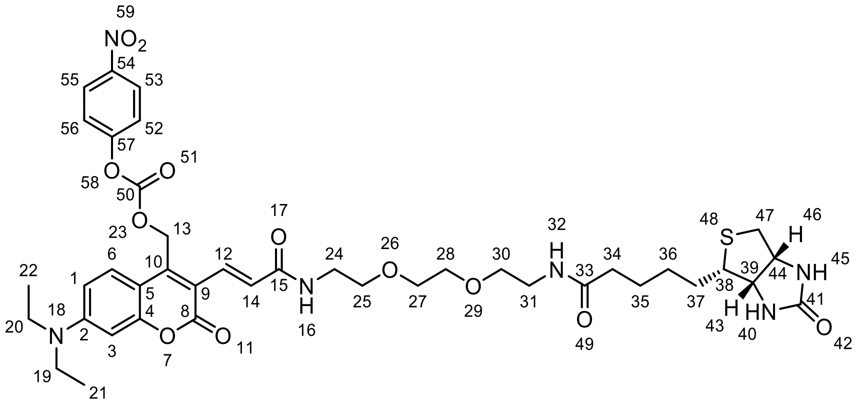

In a 10 mL round-bottom flask covered in foil, coumarin **7** (96.6 mg, 0.143 mmol) and Bis(4-nitrophenyl)carbonate (130 mg, 0.429 mmol) were dissolved in dry DMSO (2.8 mL). To this, NEt_3 (60 µL, 0.429 mmol) was added and left stirring for 2 hours, during which the reaction turned a dark orange. The DMSO was then removed with a strong flow of nitrogen overnight. The resulting residue was purified by flash column chromatography (SiO_2, 5-10% MeOH in $CHCl_3$) to yield a dark yellow solid as the desired product (49.2 mg, 0.059 mmol, 41%). $\mathbf{R_f}$ (SiO_2, 10%MeOH/$CHCl_3$) 0.37. **^{1}H-NMR (600 MHz, $CDCl_3$)** δ/ppm 8.28 – 8.25 (d, 2H, 55, 57, CH_{Ar}), 7.84 (d, *J* = 15.1 Hz, 1H, 12, C-H), 7.61 (d, *J* = 9.2 Hz, 1H, 6, CH_{Ar}), 7.43 – 7.40 (d, 2H, 54, 58, CH_{Ar}), 7.34 (d, *J* = 15.1 Hz, 1H, 14, C-H), 6.87 (t, *J* = 5.6 Hz, 1H, 16, N-H), 6.67 (dd, *J* = 9.2, 2.6 Hz, 1H, 1, CH_{Ar}), 6.51 (d, *J* = 2.8 Hz, 2H, 3, 32, CH_{Ar} and N-H), 6.02 (s, 1H, 40, N-H), 5.64 (s, 2H, 13, CH_2), 5.06 (s, 1H, 45, N-H), 4.49 (dd, *J* = 7.8, 5.0 Hz, 1H, 46, C-H), 4.32 (dd, *J* = 8.2, 4.8 Hz, 1H, 43, C-H), 3.77 – 3.53 (m, 8H, 25, 27, 28, 30, CH_2), 3.60 (q, *J* = 5.6 Hz, 2H, 24, CH_2), 3.54 – 3.41 (m, 6H, 19, 20, 31, CH_2), 3.14 (td, *J* = 7.3, 4.6 Hz, 1H, 38, C-H), 2.90 (dd, *J* = 12.7, 5.0 Hz, 1H, 47'), 2.72 (d, *J* = 13.2 Hz, 1H, 47'', CH_2), 2.22 (t, *J* = 7.3 Hz, 2H, 34, CH_2), 1.76 – 1.39 (m, 6H, 35, 36, 37, CH_2), 1.23 (t, *J* = 7.1 Hz, 6H, 21, 22, CH_2). **^{13}C-NMR (151 MHz, $CDCl_3$)** 173.39 (33), 166.71 (15), 163.62 (41), 160.48 (8), 155.82 (4), 155.51 (56), 152.30 (50), 151.56 (2), 145.70 (53), 144.81 (10), 131.20 (12), 126.77 (6), 126.45 (14), 125.48 (2C, 55, 57), 121.99 (2C, 54, 58), 115.42 (9), 109.99 (1), 107.92 (5), 97.43 (3), 70.36 and 70.24 and 69.98 and 96.93 (4C, 25, 27, 28, 30), 62.01 and 61.98 (2C, 13, 39), 60.26 (44), 55.53 (38), 45.12 (2C, 19, 20), 40.65 (47), 39.77 (24), 39.18 (31), 36.04 (34), 28.24 (37), 28.21 (36), 25.62 (35), 12.66 (2C, 21, 22). **MS (ESI$^+$)** found 839.3 [M+H$^+$]. **HRMS (ESI$^+$)** found 839.3276 (theoretical m/z = 839.3280 $[C_{40}H_{51}O_{12}N_6S_1]^+$). **IR** (film $CDCl_3$/cm^{-1}) 3293 (wb), 2980 (m, C-H), 2926, 1699 (s, C=O), 1616 (s), 1599 (s), 1523 (m, NO_2), 1350 (m, NO_2), 1313 (m), 1212 (s, C-O), 731 (m, CH_{Ar}). **UV/Vis** (0.05% DMF in H_2O) λ_{max}/nm (ε/mM^{-1} cm^{-1}) 277 (11.0), 441 (16.0)

tert-Butyl (2-(2-(2-aminoethoxy)ethoxy)ethyl)carbamate S1

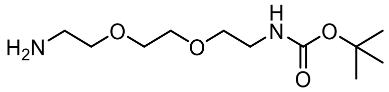

To a solution of 2,2′-(Ethylenedioxy)diethylamine (2.7 mL, 10 eq.) in CH_2Cl_2 (174 mL) at 0 °C was added di-*tert*-butyl dicarbonate (567 mg, 2.60 mmol, 0.15 eq.) and the reaction stirred for 3 hours, then allowed to warm to room temperature and left stirring overnight, during which time the reaction became turbid. The organic layer was washed with a dilute solution of NaCl (5x 100 mL), dried ($MgSO_4$), filtered and the solvent reduced in vacuo to yield a clear, colourless oil as the desired compound (484 mg, 1.95 mmol, 75%). **^{1}H-NMR (400 MHz, $CDCl_3$)** δ/ppm 5.11 (bs, 2H, amine N-H), 5.02 (bs, 1H, amide N-H), 3.61 (s, 4H, CH_2), 3.53 (dt, J = 12.6, 5.2 Hz, 4H, CH_2), 3.31 (q, J = 5.3 Hz, 2H, CH_2), 2.88 (t, J = 5.2 Hz, 2H, CH_2), 1.44 (s, 9H, CH_3). **MS (ESI$^+$)** 371.2 $[M+Na]^+$. Data in accordance with literature.[3]

Biotin-NHS S2

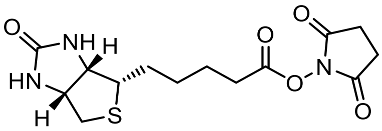

Biotin (500 mg, 2.05 mmol) and N-Hydroxysuccinimide (240 mg, 2.08 mmol) were placed into a 100 mL round bottom flask and dissolved in hot, dry DMF. To this was added dicyclohexylcarbodiimide (550 mg, 2.67 mmol) and the reaction left stirring overnight. The reaction was quenched by pouring into 100 mL of Et_2O, which formed a white precipitate. The precipitate was then filtered off and washed with Et_2O and dried to yield a white powder as the desired compound (710 mg, 2.08 mmol, >100%) with minor impurities of dicyclohexylurea. The compound was used without further purification. **^{1}H-NMR (400 MHz, DMSO-d6)** δ/ppm 6.42 (t, *J* = 1.8 Hz, 1H), 6.36 (s, 1H), 4.38 – 4.24 (m, 1H), 4.15 (ddd, *J* = 7.7, 4.4, 1.8 Hz, 1H), 3.10 (ddd, *J* = 8.1, 6.4, 4.4 Hz, 1H), 2.85 (dd, *J* = 19.3, 14.2 Hz, 1H, SCH_2), 2.81 (s, 4H, NHS-CH_2), 2.71 – 2.63 (m, 2H), 2.58 (d, *J* = 12.4 Hz, 1H), 1.78 – 1.33 (m, 6H). **MS (ESI$^+$)** found 705.2 $[2M+Na^+]$. Data in Accordance with the literature.[4]

(7-(Diethylamino)-3-((*E*)-3,14-dioxo-18-((3aS,4S,6aR)-2-oxohexahydro-1H-thieno[3,4-d]imidazol-4-yl)-7,10-dioxa-4,13-diazaoctadec-1-en-1-yl)-2-oxo-2H-chromen-4-yl)methyl prop-2-yn-1-ylcarbamate S3

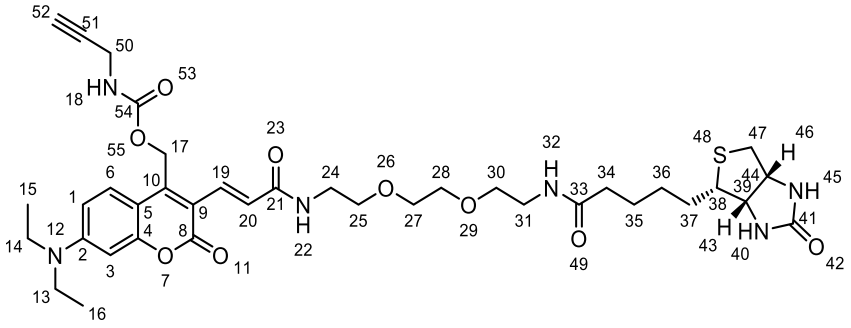

To a scintillation vial under argon equipped with a stirrer bar, 49 mg (0.058 mmol) of p-nitrophenyl-carbonate coumarin **8c** was dissolved in dry CH_2Cl_2. To this was added 94 µL (1.47 mmol, 25 eq.) of freshly distilled propargylamine and the reaction vial capped. After 40 minutes, TLC indicated complete conversion. The solvent was removed *in vacuo* and the compound purified by flash column chromatography (SiO_2, 5-10% MeOH/$CHCl_3$) to yield a bright yellow solid as the desired compound (40.2 mg, 0.052 mmol, 92%). $\mathbf{R_f}$ (SiO_2, 10% MeOH/CH_2Cl_2) 0.29. **^{1}H-NMR** (600 MHz, $CDCl_3$) δ/ppm 7.76 (d, *J* = 15.2 Hz, 1H, 19, C-H), 7.68 (d, *J* = 9.2 Hz, 1H, 6, CH_{Ar}), 7.24 (d, *J* = 15.2 Hz, 1H, 20, C-H), 7.10 (t, *J* = 5.6 Hz, 1H, 22, N-H), 6.82 (t, *J* = 5.7 Hz, 1H, 32, N-H), 6.62 (dd, *J* = 9.3, 2.6 Hz, 1H, 1, CH_{Ar}), 6.55 (s, 1H, 45, N-H), 6.43 (d, *J* = 2.5 Hz, 1H, 3, CH_{Ar}), 6.12 (t, *J* = 5.7 Hz, 1H, 18, N-H), 5.65 (s, 1H, 40, N-H), 5.47 – 5.31 (m, 2H, 17, CH_2), 4.47 (dd, *J* = 7.8, 4.9 Hz, 1H, 46, C-H), 4.34 – 4.18 (m, 1H, 43, C-H), 3.95 (dd, *J* = 5.7, 2.6 Hz, 2H, 50, CH_2), 3.68 – 3.33 (m, 12H, 24, 25, 27, 28, 30, 31, CH_2), 3.41 (q, *J* = 7.2 Hz, 4H, 13, 14, CH_2), 3.10 (td, *J* = 7.4, 4.6 Hz, 1H, 38, C-H), 2.86 (dd, *J* = 12.8, 4.9 Hz, 1H, 47', CH_2), 2.73 (d, *J* = 12.7 Hz, 1H, 47'', CH_2), 2.23 (t, *J* = 2.5 Hz, 1H, 52, C-H), 2.18 (t, *J* = 7.7 Hz, 2H, 34, CH_2), 1.78 – 1.30 (m, 6H, 35'', 36', 37'', CH_2), 1.20 (t, *J* = 7.1 Hz, 6H, 15, 16, CH_3). **^{13}C-NMR** (151 MHz, $CDCl_3$) δ/ppm 173.79 (33), 167.03 (21), 164.25 (41), 160.75 (8), 155.69 (2C, 4, 54), 151.36 (2), 147.34 (10), 131.86 (19), 127.29 (6), 125.91 (20), 115.14 (9), 109.82 (1), 108.16 (5), 97.16 (3), 79.88 (51), 71.67 (52), 70.23 and 70.15 and 69.94 and 69.91 (4C, 25, 27, 28, 30), 61.97 (39), 60.38 (44), 58.11 (17), 55.73 (38), 45.02 (2C, 13, 14), 40.56 (47), 39.74 (24), 39.19 (31), 36.08 (34), 31.01 (50), 28.33 (35, 36 or 37), 28.23 (35, 36 or 37), 25.73 (35, 36 or 37), 12.65 (2C, 15, 16). **MS (ESI$^+$)** found 755.3 $[M+H]^+$. **HRMS (ESI$^+$)** found 755.3427 (theoretical m/z = 755.3433 $[C_{37}H_{51}O_9N_6S_1]^+$). **IR** (film $CDCl_3$/cm^{-1}) 3306 (w, propargyl-CH), 2929 (w), 1699 (s, C=O), 1652 (m), 1617 (s, C=O), 1601 (s), 1456 (s), 909 (s)

Photochemistry

Photocleavage of 8b and 8c

0.5 µL of a 50 mM solution of *p*NP-coumarin **8c** or PFP-coumarin **8b** in DMF was added to 1000 µL H_2O in a Thorlabs 1 mL cuvette with blackened sides. UV-Visible recordings were performed from 260 to 600 nm on a Varian Cary 50 Bio UV-Visible Spectrophotometer. The sample was then illuminated with a ThorLabs 455 nm LED (M455L4) equipped with a collimator (COP4-A) from a distance of 20 cm in 1 minute intervals at 6/6 power, controlled by a ThorLabs Driver (LEDD1B) set at 1 Ampere maximum drive current.

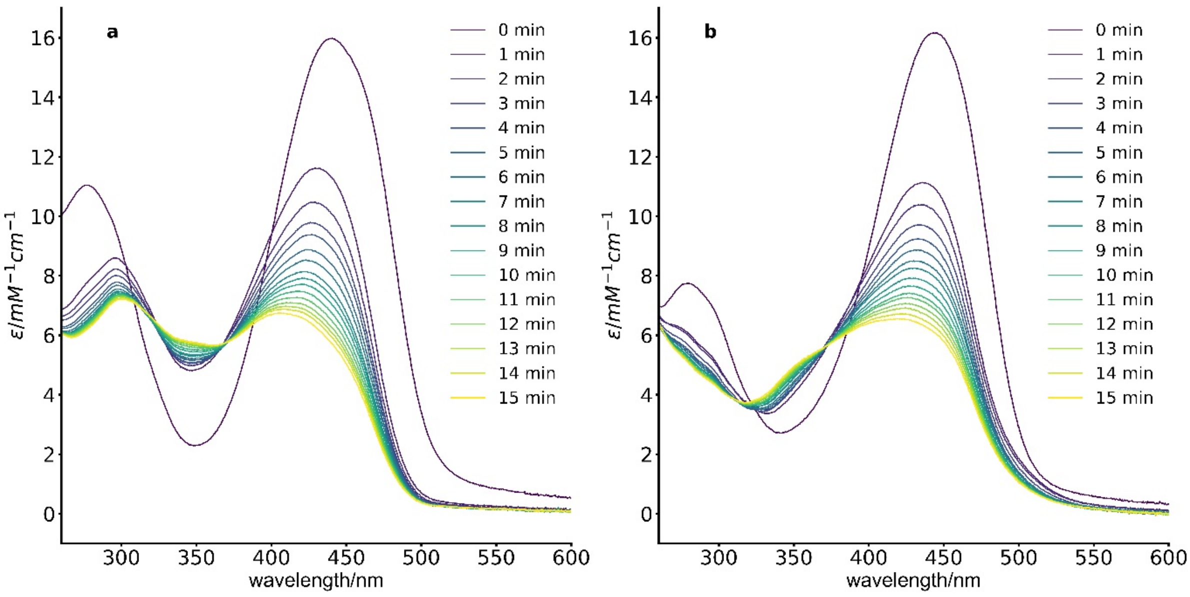

Supplementary Figure 1: Photocleavage of pNP-Coumarin **8c** (**a**) and PFP-Coumarin **8b** (**b**) in 0.05% DMF/H_2O upon irradiation with 455 nm LED. We saw a decrease in absorbance at λ_{max} of ~450 nm upon irradiation, as well as a small hypsochromic shift.

Photocleavage of Propargyl-Coumarin S3

0.5 µL of a 50 mM solution of propargyl-coumarin **S3** in DMSO was added to 1000 µL H_2O in a Thorlabs 1 mL cuvette with blackened sides. UV-Visible recordings were performed from 240 to 600 nm on a Varian Cary 50 Bio UV-Visible Spectrophotometer. The sample was then illuminated with a ThorLabs 455 nm LED (M455L4) equipped with a collimator (COP4-A) from a distance of 20 cm in 1 minute intervals at either 6/6 (A) or 1/6 power (B), controlled by a ThorLabs Driver (LEDD1B) set at 1 Ampere maximum drive current.

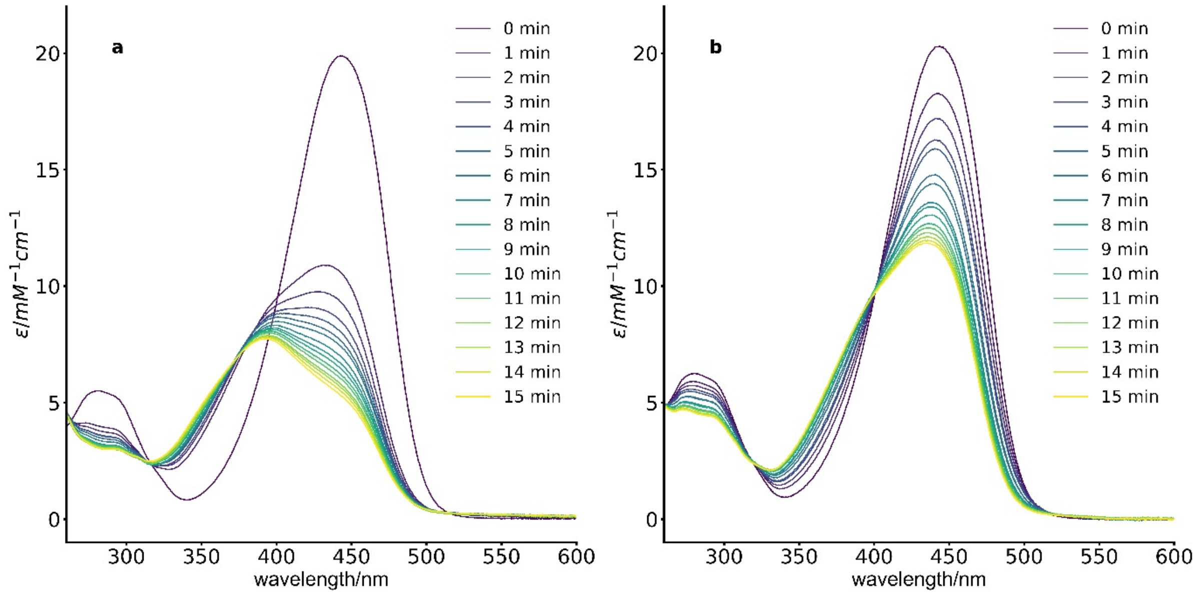

Supplementary Figure 2: Photocleavage of Propargyl-Coumarin **S3** at 6/6 (a) and 1/6 Power (b) in 0.05% DMSO/H_2O upon irradiation with 455 nm LED. We saw a decrease in absorbance at λ_{max} of ~450 nm upon irradiation, as well as a small hypsochromic shift.

In a separate experiment, 2 µL of a 25 mM solution of coumarin **9** in DMSO was added to 198 µL H_2O and 20 µL DMSO. A 20 µL aliquot was taken, then the sample was illuminated with a ThorLabs 455 nm LED (M455L4) equipped with a collimator (COP4-A) from a distance of 20 cm at 4/6 power, controlled by a ThorLabs Driver (LEDD1B) set at 1 Ampere maximum drive current. Another 20 µL aliquot was taken and both aliquots were subsequently analysed on a Waters XEVO G2-XS Q-TOF LCMS system equipped with an Acquity UPLC C18 Column (2.1 x 50 mm) using a gradient of 5-95% MeCN over 10 minutes with 0.1% formic acid throughout.

We observed full cleavage after 5 minutes of irradiation, with 2 major products. Product 1, with a m/z of 674.3 is hydroxylated compound **7**. The other photolysis product with m/z 688.3 could not be identified, but the m/z of 706.3 can be attributed to a reaction with MeOH (674.3 + 32 [CH_3OH]).

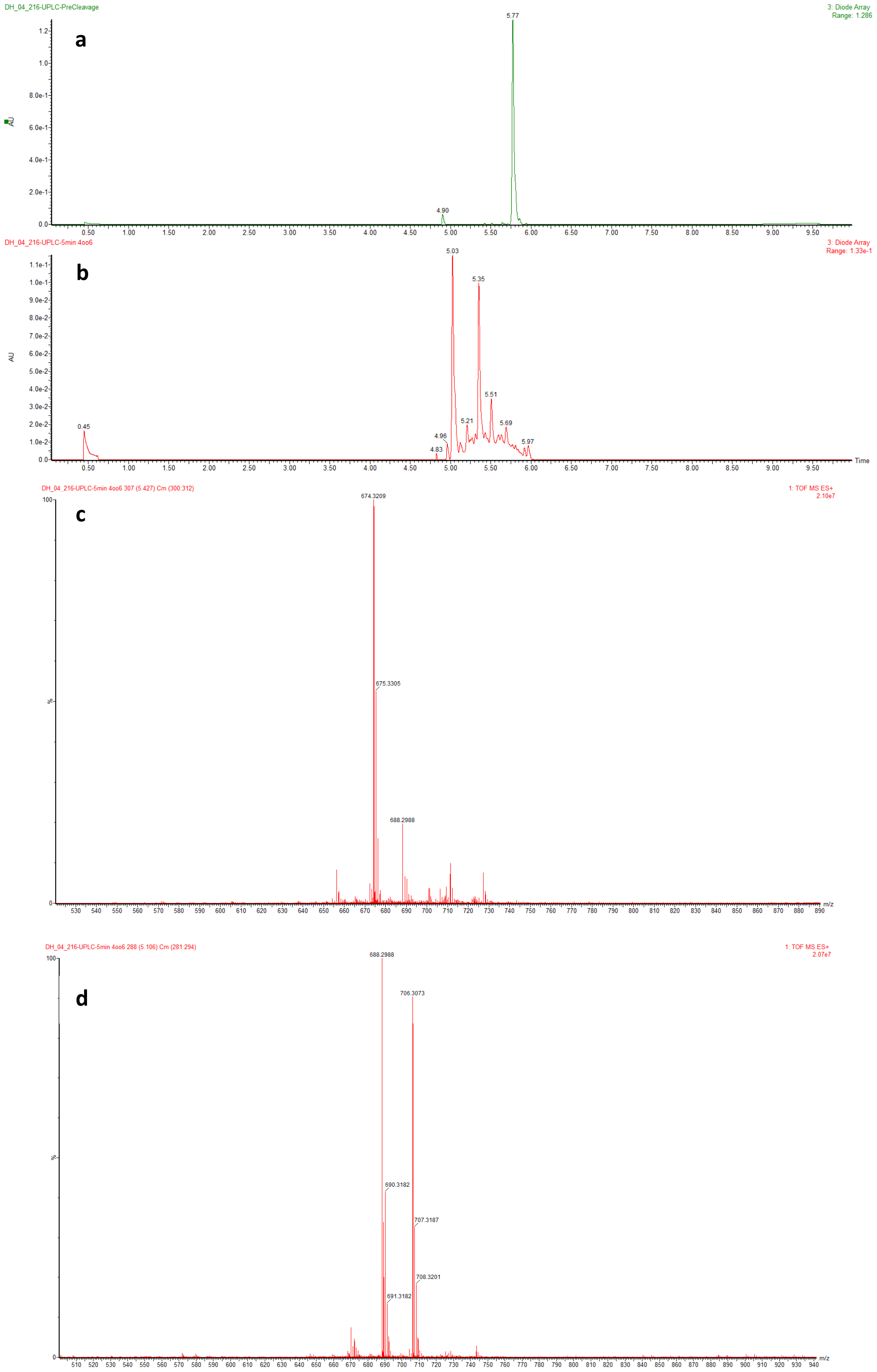

Supplementary Figure 3: Photocleavage of propargyl-coumarin **S3** and subsequent LC-MS analysis. **a/b**, LC-UV-traces of **S3** before (**a**) and after (**b**) irradiation with 455 nm LED. **c**, Mass-Spectrum of Peak at minute 5.35 in **b. d**, Mass-Spectrum of Peak at minute 5.03 in **b.**

Fluorescence of PFP-coumarin 8b and propargyl-coumarin S3

1 µL of the desired molecule as a solution of 25 mM in DMF was placed into 39 µL of H_2O. One sample was irradiated for 15 minutes with a 455 min LED at 4/6 power. The samples were then placed into a black corning 384-well plate. The wells were then measured on a Tecan Infinity M1000 plate reader and the excitation/emission of the wells was measured in the range of 390 nm to 610 nm with 3 nm steps at a gain of 120. The data was then processed to remove the majority of the auto-excitation/emission observed along the diagonal. The samples were also measured at the excitation/emission wavelengths of common fluorescent proteins at a gain of 120 and the blank measurement subtracted for the Ex/Em pairs. Protein (Ex/Em): mV (515/527), eGFP (488/507), mNG (506/517), mC (587/610).

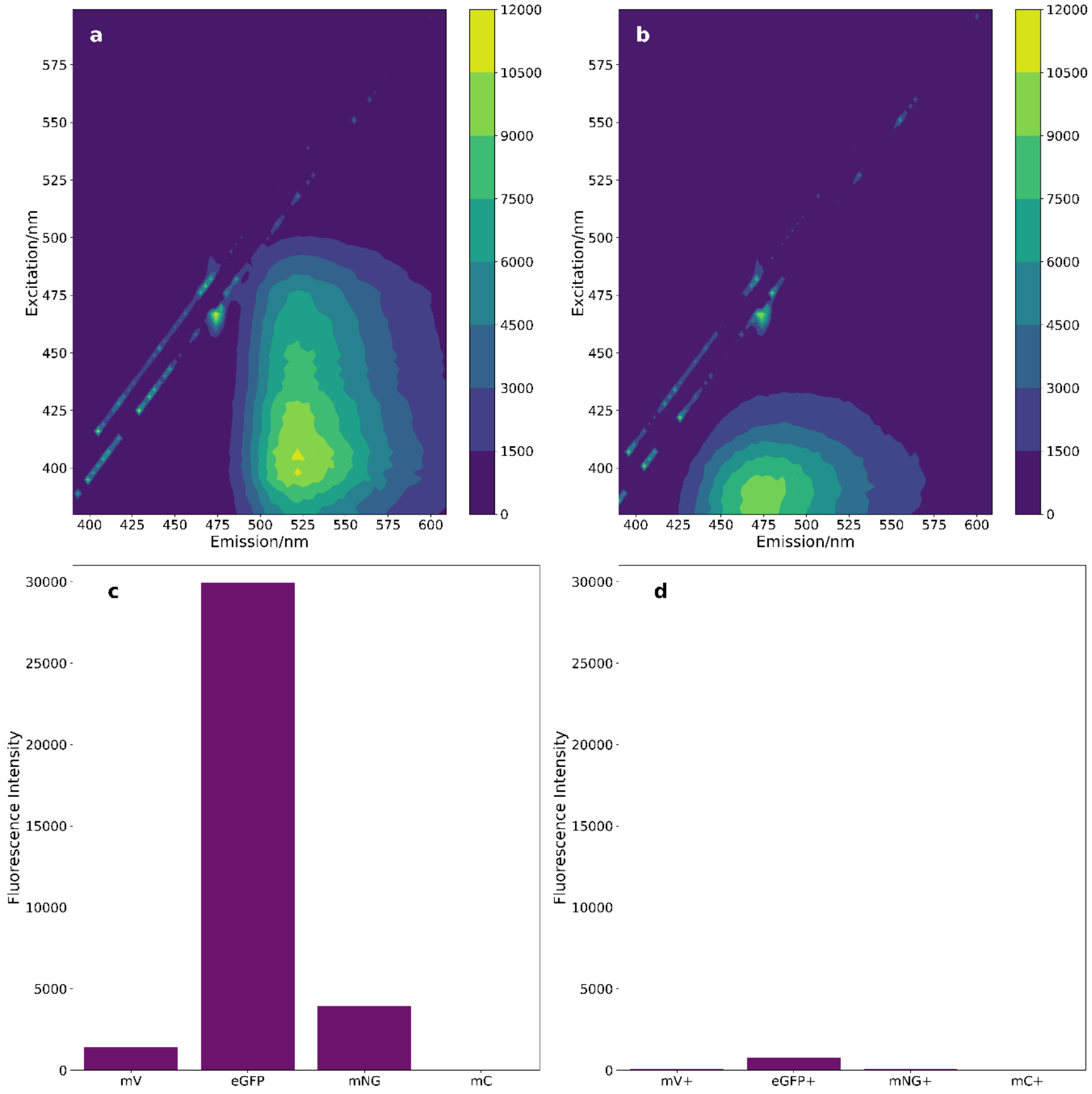

Supplementary Figure 4: Excitation/Emission of PFP-coumarin **8b** before and after irradiation as a solution of 625 µM in 2.5% DMF in H_2O. **a/b**, Ex/Em plot of PFP-Coumarin **8b** before(**a**)/after(**b**)

Irradiation with 455 nm LED for 15 minutes. **c/d**, Measured fluorescence emission of PFP-coumarin **8b** before(**c**)/after(**d**) irradiation with 455 nm LED at Ex/Em-pairs of common fluorescent proteins.

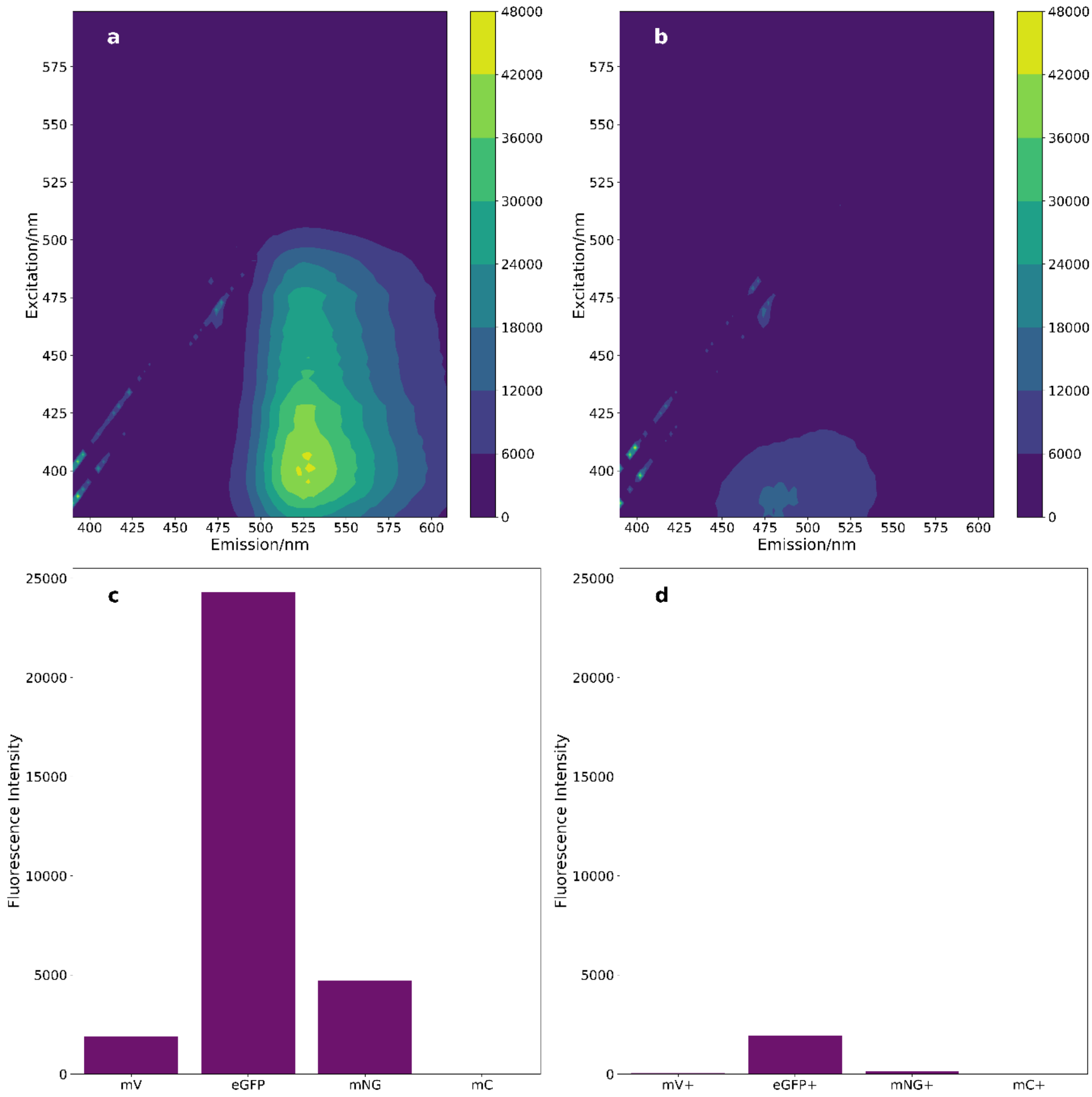

Supplementary Figure 5: Excitation/Emission of propargyl-coumarin **S3** before and after irradiation as a solution of 625 μM in 2.5% DMF in H_2O. **a**/**b**, Ex/Em plot of **S3** before (**a**)/after(**b**) Irradiation with 455 nm LED for 15 minutes. **c/d**, Measured fluorescence emission of **S3** before(**c**)/after(**d**) irradiation with 455 nm LED at Ex/Em-pairs of common fluorescent proteins.

Oligonucleotide sequences

General

Amine-modified ssDNA was purchased from atdBio and made up to 100 µM in 10 mM potassium phosphate buffer, pH 8. The modified oligonucleotide sequences employed are referenced by the number of amine modifications throughout the text.

Amine-modified T7 sequences:

Index	Nr of Amines	Length/nB	Sequence (5' - 3')
**1**	7	29	GAAATXAAXACGACXCACXAXAGGGXCXAG
**2**	4	20	2TAATXCGACTCXCTATXGGG
**3**	1	20	2TAATACGACTCACTATAGGG

Supplementary Table 1: ssDNA sequences and the corresponding number of functionalisations. X = internal C_6-hexylamino-dT modification, 2 = 5'-C_6-hexylamino-phosphate modification

PCR Primers

Index	Primer	Sequence (5'-3')
1	LA-T7 Reverse Primer	GATATAGTTCCTCCTTTCAG
2	LZFP1	CTTTAAGAAGGAGGTATACATATGACCATGATTACGGATTCACTGG C
3	LZRP2	CGAGAATTCCCGGGATCCTCATTATTTTTGACACCAGACCAAC
4	FPLacZCT	CCATTACCAGTTGGTCTGGTGTCAAAAATAATGAGGATCCCGGGA ATTC
5	RPLacZCT	GACGGCCAGTGAATCCGTAATCATGGTCATATGTATACCTCCTTC
6	α_ for_I	GCATTTATCAGGGTTATTGTCTCATGAGCGGATAC
7	α_rev_I	CTTACTCGAGAATTCCCGGGATCCTCATTATTAGGCGTGGGACGC GGC
8	α_rev_V	CCGCTCATGAGACAATAACCCTG
9	α_ for_V	AATGAGGATCCCGGGAATTCTCGAGTAAG
10	ω_for_I	GTTTAACTTTAAGAAGGAGGTATACATATGATCCCGCATCTGACCA CCAG
11	ω_rev_I	GCCTCCTGCAGGTTAACCTTAC
12	ω_rev_V	CATATGTATACCTCCTTCTTAAAGTTAAACAAAATTATTTCTAGAC
13	ω_for_V	CTCGAGTAAGGTTAACCTGCAGGAG

Supplementary Table 2: Primers used in PCR for LA-DNA templates and for the generation of *lacZ* split-β-Galactosidase

DNA Sequences of Genes:

	Gene	Sequence (5' – 3')
1	*mVenus*	GAAATTAATACGACTCACTATAGGGTCTAGAAATAATTTTGTTTA<br>ACTTTAAGAAGGAGGTATACAT**ATG**GTGAGCAAGGGCGAGGAG<br>CTGTTCACCGGGGTGGTGCCCATCCTGGTCGAGCTGGACGGC<br>GACGTAAACGGCCACAAGTTCAGCGTGTCCGGCGAGGGCGAG<br>GGCGATGCCACCTACGGCAAGCTGACCCTGAAGCTCATCTGCA<br>CCACCGGCAAGCTGCCCGTGCCCTGGCCCACCCTCGTGACCA<br>CCCTCGGCTACGGCCTGCAGTGCTTCGCCCGCTACCCCGACC<br>ACATGAAGCAGCACGACTTCTTCAAGTCCGCCATGCCCGAAGG<br>CTACGTCCAGGAGCGCACCATCTTCTTCAAGGACGACGGCAAC<br>TACAAGACCCGCGCCGAGGTGAAGTTCGAGGGCGACACCCTG<br>GTGAACCGCATCGAGCTGAAGGGCATCGACTTCAAGGAGGAC<br>GGCAACATCCTGGGGCACAAGCTGGAGTACAACTACAACAGCC<br>ACAACGTCTATATCACCGCCGACAAGCAGAAGAACGGCATCAA<br>GGCCAACTTCAAGATCCGCCACAACATCGAGGACGGCGGCGT<br>GCAGCTCGCCGACCACTACCAGCAGAACACCCCCATCGGCGA<br>CGGCCCCGTGCTGCTGCCCGACAACCACTACCTGAGCTACCA<br>GTCCAAGCTGAGCAAAGACCCCAACGAGAAGCGCGATCACAT<br>GGTCCTGCTGGAGTTCGTGACCGCCGCCGGGATCACTCTCGG<br>CATGGACGAGCTGTACAAG**TAA**TGAGGATCCCGGGAATTCTCG<br>AGTAAGGTTAACCTGCAGGAGGCCTTTAATTAAGGTGGTGCGG<br>CCGCGCTAGCGGTCCCGGGGGATCGATCCGGCTGCTAACAAA<br>GCCCGAAAGGAAGCTGAGTTGGCTGCTGCCACCGCTGAGCAA<br>TAACTAGCATAACCCCTTGGGGCCTCTAAACGGGTCTTGAGGG<br>GTTTTTTGCTGAAAGGAGGAACTATATC
2	*lacZ*	GAAATTAATACGACTCACTATAGGGTCTAGAAATAATTTTGTTTA<br>ACTTTAAGAAGGAGGTATACAT**ATG**ACCATGATTACGGATTCAC<br>TGGCCGTCGTTTTACAACGTCGTGACTGGGAAAACCCTGGCGT<br>TACCCAACTTAATCGCCTTGCAGCACATCCCCCTTTCGCCAGCT<br>GGCGTAATAGCGAAGAGGCCCGCACCGATCGCCCTTCCCAAC<br>AGTTGCGCAGCCTGAATGGCGAATGGCGCTTTGCCTGGTTTCC<br>GGCACCAGAAGCGGTGCCGGAAAGCTGGCTGGAGTGCGATCT<br>TCCTGAGGCCGATACTGTCGTCGTCCCCTCAAACTGGCAGATG

		CACGGTTACGATGCGCCCATCTACACCAACGTGACCTATCCCA<br>TTACGGTCAATCCGCCGTTTGTTCCCACGGAGAATCCGACGGG<br>TTGTTACTCGCTCACATTTAATGTTGATGAAAGCTGGCTACAGG<br>AAGGCCAGACGCGAATTATTTTTGATGGCGTTAACTCGGCGTTT<br>CATCTGTGGTGCAACGGGCGCTGGGTCGGTTACGGCCAGGAC<br>AGTCGTTTGCCGTCTGAATTTGACCTGAGCGCATTTTTACGCGC<br>CGGAGAAAACCGCCTCGCGGTGATGGTGCTGCGCTGGAGTGA<br>CGGCAGTTATCTGGAAGATCAGGATATGTGGCGGATGAGCGG<br>CATTTTCCGTGACGTCTCGTTGCTGCATAAACCGACTACACAAA<br>TCAGCGATTTCCATGTTGCCACTCGCTTTAATGATGATTTCAGC<br>CGCGCTGTACTGGAGGCTGAAGTTCAGATGTGCGGCGAGTTG<br>CGTGACTACCTACGGGTAACAGTTTCTTTATGGCAGGGTGAAA<br>CGCAGGTCGCCAGCGGCACCGCGCCTTTCGGCGGTGAAATTA<br>TCGATGAGCGTGGTGGTTATGCCGATCGCGTCACACTACGTCT<br>GAACGTCGAAAACCCGAAACTGTGGAGCGCCGAAATCCCGAAT<br>CTCTATCGTGCGGTGGTTGAACTGCACACCGCCGACGGCACG<br>CTGATTGAAGCAGAAGCCTGCGATGTCGGTTTCCGCGAGGTGC<br>GGATTGAAAATGGTCTGCTGCTGCTGAACGGCAAGCCGTTGCT<br>GATTCGAGGCGTTAACCGTCACGAGCATCATCCTCTGCATGGT<br>CAGGTCATGGATGAGCAGACGATGGTGCAGGATATCCTGCTGA<br>TGAAGCAGAACAACTTTAACGCCGTGCGCTGTTCGCATTATCC<br>GAACCATCCGCTGTGGTACACGCTGTGCGACCGCTACGGCCT<br>GTATGTGGTGGATGAAGCCAATATTGAAACCCACGGCATGGTG<br>CCAATGAATCGTCTGACCGATGATCCGCGCTGGCTACCGGCGA<br>TGAGCGAACGCGTAACGCGAATGGTGCAGCGCGATCGTAATC<br>ACCCGAGTGTGATCATCTGGTCGCTGGGGAATGAATCAGGCCA<br>CGGCGCTAATCACGACGCGCTGTATCGCTGGATCAAATCTGTC<br>GATCCTTCCCGCCCGGTGCAGTATGAAGGCGGCGGAGCCGAC<br>ACCACGGCCACCGATATTATTTGCCCGATGTACGCGCGCGTGG<br>ATGAAGACCAGCCCTTCCCGGCTGTGCCGAAATGGTCCATCAA<br>AAAATGGCTTTCGCTACCTGGAGAGACGCGCCCGCTGATCCTT<br>TGCGAATACGCCCACGCGATGGGTAACAGTCTTGGCGGTTTCG<br>CTAAATACTGGCAGGCGTTTCGTCAGTATCCCCGTTTACAGGG<br>CGGCTTCGTCTGGGACTGGGTGGATCAGTCGCTGATTAAATAT<br>GATGAAAACGGCAACCCGTGGTCGGCTTACGGCGGTGATTTTG<br>GCGATACGCCGAACGATCGCCAGTTCTGTATGAACGGTCTGGT

		CTTTGCCGACCGCACGCCGCATCCAGCGCTGACGGAAGCAAA<br>ACACCAGCAGCAGTTTTTCCAGTTCCGTTTATCCGGGCAAACC<br>ATCGAAGTGACCAGCGAATACCTGTTCCGTCATAGCGATAACG<br>AGCTCCTGCACTGGATGGTGGCGCTGGATGGTAAGCCGCTGG<br>CAAGCGGTGAAGTGCCTCTGGATGTCGCTCCACAAGGTAAACA<br>GTTGATTGAACTGCCTGAACTACCGCAGCCGGAGAGCGCCGG<br>GCAACTCTGGCTCACAGTACGCGTAGTGCAACCGAACGCGAC<br>CGCATGGTCAGAAGCCGGACACATCAGCGCCTGGCAGCAGTG<br>GCGTCTGGCTGAAAACCTCAGCGTGACACTCCCCGCCGCGTC<br>CCACGCCATCCCGCATCTGACCACCAGCGAAATGGATTTTTGC<br>ATCGAGCTGGGTAATAAGCGTTGGCAATTTAACCGCCAGTCAG<br>GCTTTCTTTCACAGATGTGGATTGGCGATAAAAAACAACTGCTG<br>ACGCCGCTGCGCGATCAGTTCACCCGTGCACCGCTGGATAAC<br>GACATTGGCGTAAGTGAAGCGACCCGCATTGACCCTAACGCCT<br>GGGTCGAACGCTGGAAGGCGGCGGGCCATTACCAGGCCGAA<br>GCAGCGTTGTTGCAGTGCACGGCAGATACACTTGCTGATGCGG<br>TGCTGATTACGACCGCTCACGCGTGGCAGCATCAGGGGAAAA<br>CCTTATTTATCAGCCGGAAAACCTACCGGATTGATGGTAGTGGT<br>CAAATGGCGATTACCGTTGATGTTGAAGTGGCGAGCGATACAC<br>CGCATCCGGCGCGGATTGGCCTGAACTGCCAGCTGGCGCAGG<br>TAGCAGAGCGGGTAAACTGGCTCGGATTAGGGCCGCAAGAAA<br>ACTATCCCGACCGCCTTACTGCCGCCTGTTTTGACCGCTGGGA<br>TCTGCCATTGTCAGACATGTATACCCCGTACGTCTTCCCGAGC<br>GAAAACGGTCTGCGCTGCGGGACGCGCGAATTGAATTATGGC<br>CCACACCAGTGGCGCGGCGACTTCCAGTTCAACATCAGCCGCT<br>ACAGTCAACAGCAACTGATGGAAACCAGCCATCGCCATCTGCT<br>GCACGCGGAAGAAGGCACATGGCTGAATATCGACGGTTTCCAT<br>ATGGGGATTGGTGGCGACGACTCCTGGAGCCCGTCAGTATCG<br>GCGGAATTCCAGCTGAGCGCCGGTCGCTACCATTACCAGTTGG<br>TCTGGTGTCAAAAA**TAA**TGAGGATCCCGGGAATTCTCGAGTAA<br>GGTTAACCTGCAGGAGGCCTTTAATTAAGGTGGTGCGGCCGC<br>GCTAGCGGTCCCGGGGGATCGATCCGGCTGCTAACAAAGCCC<br>GAAAGGAAGCTGAGTTGGCTGCTGCCACCGCTGAGCAATAACT<br>AGCATAACCCCTTGGGGCCTCTAAACGGGTCTTGAGGGGTTTT<br>TTGCTGAAAGGAGGAACTATATC

**3**	*α*	GAAATTAATACGACTCACTATAGGGTCTAGAAATAATTTTGTTTA<br>ACTTTAAGAAGGAGGTATACAT**ATG**ACCATGATTACGGATTCAC<br>TGGCCGTCGTTTTACAACGTCGTGACTGGGAAAACCCTGGCGT<br>TACCCAACTTAATCGCCTTGCAGCACATCCCCCTTTCGCCAGCT<br>GGCGTAATAGCGAAGAGGCCCGCACCGATCGCCCTTCCCAAC<br>AGTTGCGCAGCCTGAATGGCGAATGGCGCTTTGCCTGGTTTCC<br>GGCACCAGAAGCGGTGCCGGAAAGCTGGCTGGAGTGCGATCT<br>TCCTGAGGCCGATACTGTCGTCGTCCCCTCAAACTGGCAGATG<br>CACGGTTACGATGCGCCCATCTACACCAACGTGACCTATCCCA<br>TTACGGTCAATCCGCCGTTTGTTCCCACGGAGAATCCGACGGG<br>TTGTTACTCGCTCACATTTAATGTTGATGAAAGCTGGCTACAGG<br>AAGGCCAGACGCGAATTATTTTTGATGGCGTTAACTCGGCGTTT<br>CATCTGTGGTGCAACGGGCGCTGGGTCGGTTACGGCCAGGAC<br>AGTCGTTTGCCGTCTGAATTTGACCTGAGCGCATTTTTACGCGC<br>CGGAGAAAACCGCCTCGCGGTGATGGTGCTGCGCTGGAGTGA<br>CGGCAGTTATCTGGAAGATCAGGATATGTGGCGGATGAGCGG<br>CATTTTCCGTGACGTCTCGTTGCTGCATAAACCGACTACACAAA<br>TCAGCGATTTCCATGTTGCCACTCGCTTTAATGATGATTTCAGC<br>CGCGCTGTACTGGAGGCTGAAGTTCAGATGTGCGGCGAGTTG<br>CGTGACTACCTACGGGTAACAGTTTCTTTATGGCAGGGTGAAA<br>CGCAGGTCGCCAGCGGCACCGCGCCTTTCGGCGGTGAAATTA<br>TCGATGAGCGTGGTGGTTATGCCGATCGCGTCACACTACGTCT<br>GAACGTCGAAAACCCGAAACTGTGGAGCGCCGAAATCCCGAAT<br>CTCTATCGTGCGGTGGTTGAACTGCACACCGCCGACGGCACG<br>CTGATTGAAGCAGAAGCCTGCGATGTCGGTTTCCGCGAGGTGC<br>GGATTGAAAATGGTCTGCTGCTGCTGAACGGCAAGCCGTTGCT<br>GATTCGAGGCGTTAACCGTCACGAGCATCATCCTCTGCATGGT<br>CAGGTCATGGATGAGCAGACGATGGTGCAGGATATCCTGCTGA<br>TGAAGCAGAACAACTTTAACGCCGTGCGCTGTTCGCATTATCC<br>GAACCATCCGCTGTGGTACACGCTGTGCGACCGCTACGGCCT<br>GTATGTGGTGGATGAAGCCAATATTGAAACCCACGGCATGGTG<br>CCAATGAATCGTCTGACCGATGATCCGCGCTGGCTACCGGCGA<br>TGAGCGAACGCGTAACGCGAATGGTGCAGCGCGATCGTAATC<br>ACCCGAGTGTGATCATCTGGTCGCTGGGGAATGAATCAGGCCA<br>CGGCGCTAATCACGACGCGCTGTATCGCTGGATCAAATCTGTC<br>GATCCTTCCCGCCCGGTGCAGTATGAAGGCGGCGGAGCCGAC

		ACCACGGCCACCGATATTATTTGCCCGATGTACGCGCGCGTGG<br>ATGAAGACCAGCCCTTCCCGGCTGTGCCGAAATGGTCCATCAA<br>AAAATGGCTTTCGCTACCTGGAGAGACGCGCCCGCTGATCCTT<br>TGCGAATACGCCCACGCGATGGGTAACAGTCTTGGCGGTTTCG<br>CTAAATACTGGCAGGCGTTTCGTCAGTATCCCCGTTTACAGGG<br>CGGCTTCGTCTGGGACTGGGTGGATCAGTCGCTGATTAAATAT<br>GATGAAAACGGCAACCCGTGGTCGGCTTACGGCGGTGATTTTG<br>GCGATACGCCGAACGATCGCCAGTTCTGTATGAACGGTCTGGT<br>CTTTGCCGACCGCACGCCGCATCCAGCGCTGACGGAAGCAAA<br>ACACCAGCAGCAGTTTTTCCAGTTCCGTTTATCCGGGCAAACC<br>ATCGAAGTGACCAGCGAATACCTGTTCCGTCATAGCGATAACG<br>AGCTCCTGCACTGGATGGTGGCGCTGGATGGTAAGCCGCTGG<br>CAAGCGGTGAAGTGCCTCTGGATGTCGCTCCACAAGGTAAACA<br>GTTGATTGAACTGCCTGAACTACCGCAGCCGGAGAGCGCCGG<br>GCAACTCTGGCTCACAGTACGCGTAGTGCAACCGAACGCGAC<br>CGCATGGTCAGAAGCCGGACACATCAGCGCCTGGCAGCAGTG<br>GCGTCTGGCTGAAAACCTCAGCGTGACACTCCCCGCCGCGTC<br>CCACGCC**TAA**TAATGAGGATCCCGGGAATTCTCGAGTAAGGTT<br>AACCTGCAGGAGGCCTTTAATTAAGGTGGTGCGGCCGCGCTA<br>GCGGTCCCGGGGGATCGATCCGGCTGCTAACAAAGCCCGAAA<br>GGAAGCTGAGTTGGCTGCTGCCACCGCTGAGCAATAACTAGCA<br>TAACCCCTTGGGGCCTCTAAACGGGTCTTGAGGGGTTTTTTGC<br>TGAAAGGAGGAACTATATCCGGAA
**4**	*ω*	GAAATTAATACGACTCACTATAGGGTCTAGAAATAATTTTGTTTA<br>ACTTTAAGAAGGAGGTATACAT**ATG**ATCCCGCATCTGACCACCA<br>GCGAAATGGATTTTTGCATCGAGCTGGGTAATAAGCGTTGGCA<br>ATTTAACCGCCAGTCAGGCTTTCTTTCACAGATGTGGATTGGCG<br>ATAAAAAACAACTGCTGACGCCGCTGCGCGATCAGTTCACCCG<br>TGCACCGCTGGATAACGACATTGGCGTAAGTGAAGCGACCCG<br>CATTGACCCTAACGCCTGGGTCGAACGCTGGAAGGCGGCGGG<br>CCATTACCAGGCCGAAGCAGCGTTGTTGCAGTGCACGGCAGAT<br>ACACTTGCTGATGCGGTGCTGATTACGACCGCTCACGCGTGGC<br>AGCATCAGGGGAAAACCTTATTTATCAGCCGGAAAACCTACCG<br>GATTGATGGTAGTGGTCAAATGGCGATTACCGTTGATGTTGAA<br>GTGGCGAGCGATACACCGCATCCGGCGCGGATTGGCCTGAAC<br>TGCCAGCTGGCGCAGGTAGCAGAGCGGGTAAACTGGCTCGGA

		TTAGGGCCGCAAGAAAACTATCCCGACCGCCTTACTGCCGCCTGTTTTGACCGCTGGGATCTGCCATTGTCAGACATGTATACCCCGTACGTCTTCCCGAGCGAAAACGGTCTGCGCTGCGGGACGCGCGAATTGAATTATGGCCCACACCAGTGGCGCGGCGACTTCCAGTTCAACATCAGCCGCTACAGTCAACAGCAACTGATGGAAACCAGCCATCGCCATCTGCTGCACGCGGAAGAAGGCACATGGCTGAATATCGACGGTTTCCATATGGGGATTGGTGGCGACGACTCCTGGAGCCCGTCAGTATCGGCGGAATTCCAGCTGAGCGCCGGTCGCTACCATTACCAGTTGGTCTGGTGTCAAAAA**TAA**TGAGGATCCCGGGAATTCTCGAGTAAGGTTAACCTGCAGGAGGCCTTTAATTAAGGTGGTGCGGCCGCGCTAGCGGTCCCGGGGGATCGATCCGGCTGCTAACAAAGCCCGAAAGGAAGCTGAGTTGGCTGCTGCCACCGCTGAGCAATAACTAGCATAACCCCTTGGGGCCTCTAAACGGGTCTTGAGGGGTTTTTTGCTGAAAGGAGGAACTATATC
**5**	LA-T7-Broccoli Template Strand	GGGTCTAGGAGCCCACACTCTACTCGACAGATACGAATATCTGGACCCGACCGTCTCCTAGACCCTATAGTGAGTCGTATTAATTC
**6**	LA-T7 Broccoli Complementary Strand	GAGACGGTCGGGTCCAGATATTCGTATCTGTCGAGTAGAGTGTGGGCTCCTAGACCC
**7**	*mNeonGreen*	GAAATTAATACGACTCACTATAGGGTCTAGAAATAATTTTGTTTAACTTTAAGAAGGAGGTATACATATGGTGAGCAAAGGCGAAGAGGATAATATGGCAAGCCTGCCTGCAACAC**ATG**AACTGCATATTTTTGGTAGCATTAACGGCGTGGATTTTGATATGGTTGGTCAAGGCACCGGTAATCCGAATGATGGTTATGAAGAACTGAATCTGAAAAGCACCAAAGGCGATCTGCAGTTTAGCCCGTGGATTCTGGTTCCGCATATTGGTTATGGTTTTCATCAGTATCTGCCGTATCCGGATGGTATGAGCCCGTTTCAGGCAGCAATGGTTGATGGTAGCGGTTATCAGGTTCATCGTACCATGCAGTTTGAAGATGGTGCAAGCCTGACCGTTAATTATCGTTATACCTATGAAGGCAGCCACATTAAAGGTGAAGCACAGGTTAAAGGTACAGGTTTTCCGGCAGATGGTCCGGTTATGACCAATAGTCTGACCGCAGCAGATTGGTGTCGTAGCAAAAAAACCTATCCGAACGATAAAACCATCATCAGCACCTTCAAAT

		GGTCATATACCACCGGCAATGGTAAACGTTATCGTAGCACCGC<br>ACGTACCACCTATACCTTTGCAAAACCGATGGCAGCAAACTATC<br>TGAAAAATCAGCCGATGTATGTGTTTCGCAAAACGGAACTGAAA<br>CATTCCAAAACCGAGCTGAACTTTAAAGAATGGCAGAAAGCATT<br>TACCGATGTGATGGGTATGGATGAGCTGTACAAA**TAA**TGAGGA<br>TCCCGGGAATTCTCGAGTAAGGTTAACCTGCAGGAGGCCTTTA<br>ATTAAGGTGGTGCGGCCGCGCTAGCGGTCCCGGGGGATCGAT<br>CCGGCTGCTAACAAAGCCCGAAAGGAAGCTGAGTTGGCTGCT<br>GCCACCGCTGAGCAATAACTAGCATAACCCCTTGGGGCCTCTA<br>AACGGGTCTTGAGGGGTTTTTTGCTGAAAGGAGGAACTATATC

Supplementary Table 3: Sequences of linear DNA templates used for cell-free expression, produced by PCR. T7-Promoter and -Terminator Regions are underlined, protein start- and stop-codons are in bold.

Chemical Biology Methods

General Chemical Biology Methods

All experiments involving bLA-DNA were performed under reduced ambient lighting (direct overhead lights turned off). Polyacrylamide/Bisacrylamide (19:1) (PAA/BisPAA), Urea, Ammonium Persulfate (APS) and Tris-Borate-EDTA (TBE) buffer were purchased from Sigma-Aldrich. Agarose (SeaKem®, low electroendosmosis) was purchased from Lonza Bioscience. UltraPure™ Tris-Acetate-EDTA (TAE) buffer (10X) was purchased from ThermoFisher. *N,N*-tetramethyl ethylene diamine (TMEDA) was purchased from Bio-Rad. Egg-PC was purchased from Avanti Polar Lipids. Water used was purified using a Milli-Q® (Merck Millipore) water purification system. Gels were visualised on a BioRad Gel Doc XR+ gel imager. PCRs and Incubations were performed in an Applied Bioscience 2720, Applied Bioscience ProFlex™ or Peqlabs peqSTAR thermocycler. High Performance Liquid Chromatography (HPLC) purifications were performed on an Agilent 1260 Infinity system. No unexpected or unusually high safety hazards were encountered.

Data Analysis and Plotting

Data was plotted using python's matplotlib and seaborn libraries. Error bars and confidence intervals were computed using seaborn and show a 95% confidence interval. T-tests were performed using python's scipy library, using the stats.ttest_ind() method and a two-tailed test was applied. Activation percentages were computed using the following equation: $\frac{\mu_S - \mu_N}{\mu_R - \mu_N} \times 100$, where μ = mean, N = background sample, S = sample of interest, R = reference sample. Gel electrophoresis data was analysed using ImageLab software.

Polyacrylamide Gel Electrophoresis (PAGE)

Gels were cast by hand with the BioRad Mini-PROTEAN® handcasting equipment at a thickness of 0.75 mm. Denaturing gels were run with 16% PAA/BisAA (19:1), 7M Urea, 1x TBE Buffer (polymerised using 0.8% APS and 0.05% TMEDA) at 250 V in 1X TBE buffer. Samples were loaded with 95% formamide, 0.015% SDS, 5 mM EDTA, 0.025% bromophenol blue and 0.025% xylene cyanol. Gels were stained with Gel-Red® nucleic acid stain (Biotium).

Agarose Gel Electrophoresis (AGE)

DNA Gels were prepared at 1% agarose (for DNA with bound streptavidin) in 1X TAE buffer and run at 100 V in 1x TAE buffer. Samples were prepared with purple loading dye (NEB, B7024S) and run against a 1kb DNA ladder (NEB, N3232S). Gels were stained with Gel-Red® nucleic acid stain (Biotium).

RNA Gels were prepared at 2% agarose in 1x TBE buffer, prestained with Sybr® Green II and run at 100 V in 1x TBE buffer. Samples were prepared using RNA loading dye (NEB B0363S) and heated to 70 °C for 10 minutes and cooled on ice before loading to denature the RNA.

Samples were run against a low-range ssRNA ladder (NEB, N0364S) or ssRNA ladder (NEB, N0362S).

Preparation of UV light-activatable T7 Primer (uvLA-T7)

Method adapted from Booth *et al.*[5] In a 0.5 mL Eppendorf DNA LoBind Tube, 7-amino-T7 DNA (**Supplementary Table 1**, Entry 1, 10 µM), $NaHCO_3$ (100 mM) and PC Biotin-NHS ester (5 mM, Click Chemistry Tools (CCT), Catalogue Nr: 1225) were added in 50% DMF in a total reaction volume of 100 µL. The reaction mixture was vortexed, spun down using a table-top centrifuge and placed in a Thermomixer (Eppendorf) and shaken at 700 rpm, at room temperature, overnight. The reaction mixture was then washed 3 times with chloroform (200 µL) to remove the majority of organic contaminants, diluted to 100 µL with H_2O, spun for 1 minute at 14k RCF to pellet any insoluble impurities and the supernatant transferred to a HPLC injection vial. The oligonucleotide was then purified by HPLC on an Agilent Polaris C18 column (150 x 4.6 mm), heated to 50 °C using a gradient of 5-30% MeCN over 27 minutes, with 10 mM triethylammonium bicarbonate (TEAB) pH 8.5 as an ion-pairing buffer throughout. The DNA was analysed by LC-MS and LC-UV for purity (*vide infra*) and used in downstream applications (uvLA-mVenus **(Figure 3**), uvLA-α (**Figure 5**, f-h) and CCT-α (**Supplementary Figures 22, 23, 27, and 32**).

Analogous to the above, the preparation of the UV-activatable primer using the PC-Biotin-NHS molecule from AmberGen (PCB-N-001) was performed as previously described[5] on a 50 µL scale and purity was confirmed using oligonucleotide LC-MS (*vide infra*). This was used in **Figure 4** and as AmberGen-α in **Supplementary Figures 22, 23, 25, and 26**.

Preparation of blue light-activatable-T7 Primer (bLA-T7)

In a 0.5 mL Eppendorf DNA LoBind Tube, 7-amino-T7 DNA (**Supplementary Table 1**, Entry 1, 11.1 µM), MOPS Buffer pH 8.5 (111 mM) and PFP-Coumarin **8b** (13.89 mM) were added in 70% DMF in a total reaction volume of 34 µL. The reaction mixture was vortexed, centrifuged using a table-top centrifuge and placed in a Thermomixer (Eppendorf), heated to 37 °C and shaken at 800 rpm for 3 hours and then at room temperature overnight. The reaction mixture was then washed 3 times with chloroform to remove the majority of organic contaminants, diluted to 100 µL with 1:1 MeCN:H_2O, spun for 1 minute at 14k RCF to pellet any insoluble impurities and the supernatant transferred to a HPLC injection vial. The oligonucleotide was then purified by HPLC on an Agilent Polaris C18 column (150 x 4.6 mm) heated to 50 °C using a gradient of 5-34% MeCN over 36 minutes, with 10 mM triethylammonium bicarbonate (TEAB) pH 8.5 as an ion-pairing buffer throughout. The DNA was then analysed by LC-MS and LC-UV for purity (*vide infra*), and used in downstream applications.

Annealing of Broccoli-Construct

Amine-only-T7 Broccoli DNA was assembled by mixing the template and non-template strands (**Supplementary Table 3**, Entry 5 and 6) in an equimolar ratio (1 µL each of a solution of 10 µM in 10 mM Tris pH 8). To this was then added the 7-amine-containing T7 promoter sequence (**Supplementary Table 1**, Entry 1, 1 µL of a 10 µM solution in 10 mM Tris pH 8) and the volume adjusted to 10 µL with H_2O. Similarly, blue light-activatable Broccoli was assembled by mixing template and non-template strands in an equimolar ratio (0.5 µL each of a solution of 10 µM in 10 mM Tris pH 8) and the blue-light activatable T7 promoter sequence in 4-fold excess (5.41 µL of a 3.7 µM solution in H_2O) and the volume was adjusted to 7 µL with H_2O. The samples were then heated to 95 °C and slowly cooled to 25 °C over 40 minutes in a thermocycler. To this was then added a 50x molar excess of monovalent streptavidin, as described below.

Plasmid Digestion

Plasmids employed include *mVenus*,[5] *mNeonGreen*[6] and *lacZ*, including α and ω, (described below) inside the PURExpress Control Template.

Full-Length plasmid (5 ng/µl) was digested using HindIII-High Fidelity (0.8 U/µL, NEB R3104S) in 1x CutSmart Buffer (NEB B7204S) in a total volume of 25 µL. The solution was incubated for 30 minutes at 37 °C and then heated to 80 °C for 20 minutes. The resulting digested plasmid was used without further purification in PCR.

Preparation of LA-DNA Templates using PCR

PCR reactions were carried out using DreamTaq DNA polymerase MasterMix (2x, ThermoFisher), reverse primer **1** (**Supplementary Table 2**) at 0.25 µM concentration, uvLA-T7 primer at 0.325 µM effective concentration or bLA-T7 primer at an 0.8 µM effective concentration (determined by A_{260} absorbance) and 0.04 ng/µL of the desired, digested plasmid as template in a total reaction volume of 25 µL. The PCR was carried out according to the manufacturer's protocol for 35 cycles with an annealing temperature of 47 °C for 30 seconds (for uvLA-DNA) or 45 °C for 45 seconds (for bLA-DNA), an extension time of 72 °C for 1 min 15s/kbp and a final extension at 72 °C for 10 minutes. The resulting DNA was then purified using the GeneJet PCR purification columns (ThermoFisher) following the manufacturer's protocol and eluted in 25 µL H_2O.

Amine-only control DNA was prepared using the same method as for uvLA-DNA, with 0.2 µM forward and reverse primer (**Supplementary Table 1,** Entry 1, and **Supplementary Table 2,** Entry 1). Unmodified linear templates were prepared following a previously reported protocol.[7]

Streptavidin binding onto T7-modified genes

To cage DNA, monovalent streptavidin (provided by Howarth's laboratory, Department of Biochemistry, University of Oxford) was bound to the DNA templates containing the modified T7–promoter. The PCR product was incubated with a 50× molar excess of monovalent streptavidin (1 mg/mL in phosphate buffered saline (PBS), pH 7.4)) in protein LoBind tubes (Eppendorf) overnight at 4 °C. Amine-only DNA was also incubated with monovalent streptavidin in the same manner.

Illumination of Samples

Samples were illuminated in 200 µL PCR tubes with open lids and held in a PCR tube rack over aluminium foil. Irradiation was then performed top-down with a ThorLabs 455 nm LED (M455L4) equipped with a collimator (COP4-A) from a distance of 30 cm at an irradiance of 64 $mW{\cdot}cm^{-2}$ (power setting 4/6, unless otherwise indicated; 1/6 power corresponds to an irradiance of 7.34 $mW{\cdot}cm^{-2}$, 2/6 power corresponds to an irradiance of 17.54 $mW{\cdot}cm^{-2}$, 6/6 power corresponds to an irradiance of 134 $mW{\cdot}cm^{-2}$) or a ThorLabs 365 nm LED (M365L3) equipped with a collimator (COP5-A) from a distance of 34 cm at an irradiance of 1.06 $mW{\cdot}cm^{-2}$ (~2/6 power setting), both controlled by a ThorLabs driver (LEDD1B) set at 1 A maximum drive current.

Transcription of Broccoli Aptamer

In vitro transcription of broccoli aptamer was performed using 3 ng/µL of template DNA and 2 U/µL T7 RNA polymerase (ThermoFisher) in 40 mM Tris-HCl pH 8.0, 2 mM spermidine, 10 mM DTT, 48 mM $MgCl_2$, 10 mM each of UTP, CTP, ATP and GTP. Samples were illuminated for 1 minute using a 455 nm LED (if required) before the addition of DFHBI to a concentration of 60 µM. The total reaction volume is 6 µL. Samples were then incubated at 37 °C for 4 hours. 5 µL aliquots were then taken and diluted to 41 µL with water. 40 µL of the resulting solutions were then placed into a black polystyrene 384 well plate (OptiPlate, PerkinElmer) and fluorescence measurements were taken on a plate reader (Tecan infinite M1000Pro) using $\lambda_{ex/em}$:455/506 nm with a bandwidth of 10 nm.

Transcription of mVenus, α- and ω-Subunit mRNA

In vitro transcription was performed using 10 ng/µL (*mVenus*) or 3 ng/µL (α- and ω-subunit) of template DNA and 2 U/µL T7 RNA polymerase (ThermoFisher) in 40 mM Tris-HCl pH 8.0, 2 mM spermidine, 10 mM DTT, 48 mM $MgCl_2$, 10 mM each of UTP, CTP, ATP and GTP. Samples were then illuminated for 1 minute using a 455 nm LED or 3 minutes using a 365 nm LED, as described. Samples were incubated at 37 °C for 4 hours and 1.5 µL of the resulting solution was then diluted into 10 µL of water. Samples were denatured using 2x RNA loading dye (NEB) and analysed by AGE.

Cell-Free Protein Synthesis

In a 200 µL PCR tube, 10 ng/µl of linear template DNA (amine only or modified, both with 50x molar xs. mSA) were added to PURExpress® (NEB, E6800) with 2.67 U/µL RNase Inhibitor (murine, NEB, M0314 or RNaseOUT, recombinant *E. coli,* ThermoFisher), as prepared by the manufacturer's protocol, in a total volume of 3 µL. The resulting solutions were kept at room temperature in the dark and illuminated as required, before placing them in a thermocycler and incubated at 37 °C for 4 hours. 2 µL of each solution was then placed into 39 µL of H_2O and mixed by pipetting. 40 µL of the resulting solutions were then transferred into a 384 well plate and placed into a plate reader (Tecan Infinity M1000) and fluorescence measurements were taken ($\lambda_{Ex/Em}$: 515/527 nm for mVenus, Gain: 173).

For the light titration as well as for the unmodified vs. amino-modified DNA comparison (**Supplementary Figures 18 and 19**), reactions were prepared as above on a 2 µL scale, and after incubation, 1.6 µL of the solutions were added to 39.4 µL H_2O, mixed pipetting and 40 µL transferred into the 384 well plate for measurement as above.

Similarly, split β-Gal was expressed by the addition of 3 ng/ul of each α- and ω-template (amine only, or modified, both with 50x molar xs. mSA) as above. After incubation at 37 °C for 4 hours, 2 µL of each solution was then placed into 39 µL of a cold solution of carboxyumbelliferyl-β-ᴅ-galactopyranoside (CUG, Abcam, ab275047) in H_2O (100 µM) on ice and mixed by pipetting. 40 µL of the resulting solutions were then transferred into a pre-chilled 384 well plate, placed into a plate reader (Tecan Infinity M1000) heated to 37 °C and fluorescence measurements were taken at regular intervals ($\lambda_{Ex/Em}$: 388/455 nm, Gain: 100).

Cloning of β-Galactosidase within the PURExpress control template

To prepare *lacZ* inside the PURExpress control template plasmid, genomic DNA was extracted from BL21 DE3 *E. Coli* cells through phenol/chloroform extraction using standard procedures and the resulting DNA precipitated by addition of 10%vol. NaOAc pH 5 followed by 2.5 volumes of cold ethanol. The solution was then placed at -20 °C for a minimum of 2 hours before centrifugation at 16000 xg for 30 minutes at 4 °C. The supernatant was removed and the pellet washed twice with 70% ethanol and centrifuged again for 15 minutes, the supernatant removed, the pellet dried and resuspended in 10 mM Tris pH 8.

The β-Gal gene insert and plasmid vector of the PURExpress control template were then prepared using PCR, with primers containing homologous regions (**Supplementary Table 2**).

PCR cycle conditions can be found below. The β-Gal gene insert was prepared using Phusion Polymerase MasterMix (2x, NEB), with primers (**Supplementary Table 2**, Entries 2 and 3) at 0.5 µM concentrations and 0.06 ng/µL of the BL21 DE3 genomic DNA as template, as prepared above. The control template vector was prepared using Phusion Polymerase

MasterMix (2x, NEB), with primers (**Supplementary Table 2**, Entries 4 and 5) at 0.25 µM concentrations and 0.06 ng/µL of NdeI digested PURExpress Control Template as template. The PCR products were purified using the GeneJet PCR Purification Kit (ThermoFisher) according to the manufacturer's protocol. The insert and plasmid backbone DNA were mixed in a 1:4 ratio and 100 ng of the DNA transformed into 20 µL XL-10 Gold Ultracompetent cells (Agilent) via heat shock. The cells were plated on ampicillin containing LB Agar plates and grown for 16 hours. Individual colonies were picked and grown in 5 mL of LB with ampicillin for 16 hours at 37 °C in a shaking incubator at 200 rpm. The plasmid DNA was extracted using the QIAprep Miniprep Kit using the manufacturer's protocol and the sequence confirmed by Sanger sequencing (Source Bioscience).

For PCR amplification of the *lacZ* insert with overlapping regions with the control template backbone, the following cycle conditions were used:

<table>
<tr><th>Temp</th><th colspan="2">Time</th></tr>
<tr><td>98 °C</td><td colspan="2">30 s</td></tr>
<tr><td>98 °C</td><td rowspan="3">34x</td><td>10 s</td></tr>
<tr><td>55 °C</td><td>30 s</td></tr>
<tr><td>72 °C</td><td>1 min 34 s</td></tr>
<tr><td>72 °C</td><td colspan="2">10 mins</td></tr>
<tr><td>4 °C</td><td colspan="2">HOLD</td></tr>
</table>

For PCR amplification of the control template vector with overlapping regions with the *lacZ* insert, the following cycle conditions were used:

<table>
<tr><th>Temp</th><th colspan="2">Time</th></tr>
<tr><td>98 °C</td><td colspan="2">30 s</td></tr>
<tr><td>98 °C</td><td rowspan="3">35x</td><td>10 s</td></tr>
<tr><td>55 °C</td><td>30 s</td></tr>
<tr><td>72 °C</td><td>1 min 10 s</td></tr>
<tr><td>72 °C</td><td colspan="2">10 mins</td></tr>
<tr><td>4 °C</td><td colspan="2">HOLD</td></tr>
</table>

Generation of Split β-Galactosidase

Adapted from a procedure reported by J. P. Basilion.[8] Full Length β-Galactosidase inside the PURExpress control template was used as the starting genetic template (preparation described above) and split at amino-acid residue 738. The final constructs contained amino acids 1-737 (subunit α) and 738-1024 (subunit ω, with a starting methionine added).

Linear DNA inserts containing the relevant split protein parts as well as vectors to generate full-length plasmid, with homologous regions for recombination to full-length plasmid, were

generated via PCR (**Supplementary Table 2**). PCR cycle conditions can be found below. PCR reactions were carried out using Phusion DNA polymerase MasterMix (2x, NEB), primers (forward and reverse) at 0.5 μM concentration, 3% DMSO and 0.04 ng/μL full-length β-Galactosidase plasmid as template in a total reaction volume of 25 μL. 2 μL of the PCR reactions were then analysed by AGE and the to the remainder was added 0.5 μL of Dpn1 (NEB, R0176S, 10 U) and incubated at 37 °C for 1 hour to digest the plasmid template, and then the enzyme heat deactivated at 80 °C for 20 minutes. The DNA was purified using the GeneJet PCR purification kit.

For the α-insert, using Primers 6 and 7 (**Supplementary Table 2**), the following cycle conditions were used:

<table>
<tr><th>Temp</th><th colspan="2">Time</th></tr>
<tr><td>98 °C</td><td colspan="2">30 s</td></tr>
<tr><td>98 °C</td><td rowspan="3">30x</td><td>10 s</td></tr>
<tr><td>69 °C</td><td>20 s</td></tr>
<tr><td>72 °C</td><td>1 min 30 s</td></tr>
<tr><td>72 °C</td><td colspan="2">5 mins</td></tr>
<tr><td>4 °C</td><td colspan="2">HOLD</td></tr>
</table>

For the α-vector, using primers 8 and 9 (**Supplementary Table 2**), the following cycle conditions were used:

<table>
<tr><th>Temp</th><th colspan="2">Time</th></tr>
<tr><td>98 °C</td><td colspan="2">30 s</td></tr>
<tr><td>98 °C</td><td rowspan="3">30x</td><td>10 s</td></tr>
<tr><td>66 °C</td><td>20 s</td></tr>
<tr><td>72 °C</td><td>1 min 30 s</td></tr>
<tr><td>72 °C</td><td colspan="2">5 mins</td></tr>
<tr><td>4 °C</td><td colspan="2">HOLD</td></tr>
</table>

For the ω-insert, using Primers 10 and 11 (**Supplementary Table 2**), the following cycle conditions were used

<table>
<tr><th>Temp</th><th colspan="2">Time</th></tr>
<tr><td>98 °C</td><td colspan="2">30 s</td></tr>
<tr><td>98 °C</td><td rowspan="3">30x</td><td>10 s</td></tr>
<tr><td>66 °C</td><td>20 s</td></tr>
<tr><td>72 °C</td><td>30 s</td></tr>
<tr><td>72 °C</td><td colspan="2">5 mins</td></tr>
<tr><td>4 °C</td><td colspan="2">HOLD</td></tr>
</table>

For the ω-vector, using Primers 12 and 13 (**Supplementary Table 2**), the following cycle conditions were used:

Temp	Time	
98 °C	30 s	
98 °C	30x	10 s
67 °C		20 s
72 °C		1 min 30 s
72 °C	5 mins	
4 °C	HOLD	

2 µL of the PCR reactions were then analysed using AGE to confirm the correct product (**Supplementary Figure 21**).

The linear templates were combined using homologous recombination. For this, the insert and vector were mixed in a 1:1 ratio (for the α-4 subunit) or a 3:1 ratio (for the ω subunit) and 100 ng total was added to 15 µL of XL-10 gold *E. Coli* cells (Agilent) in a 14 mL round-bottom centrifuge tube (Falcon). The tube was flicked gently to mix and incubated on ice for 30 minutes. The tubes were placed in a 42 °C water bath for 45 seconds and then immediately placed on ice for around 2 minutes. To the tubes was then added 300 µL of SOC outgrowth medium (NEB, B9020S) and incubated in a shaking incubator at 250 rpm for 1 hour. 100 µL of the resulting solution was spread on ampicillin-containing agar plates and grown at 37 °C overnight.

Colonies positive for the desired gene were then used to inoculate 5 mL of Lysogeny Broth containing ampicillin in 50 mL centrifuge tubes and grown overnight at 37 °C in a shaking incubator at 200 rpm. Minipreparations were then carried out using the Promega Miniprep Kit, following the manufacturer's protocol and the sequences confirmed by Sanger sequencing (Source Bioscience).

Preparation of β-Galactosidase Calibration Curve

2 µL of a known amount of full-length β-Galactosidase (Merck, 48275-1mg-F) dissolved in 50% glycerol was added to 39 µL of an ice-cold solution of CUG (100 µM). The solution was mixed by pipetting before transferring 40 µL into a chilled 384-well plate. The plate was then placed into a plate reader (Tecan Infinity M1000) heated to 37 °C and fluorescence measurements were taken after 10 minutes of incubation ($\lambda_{Ex/Em}$: 388/455 nm, Gain: 100).

Preparation of Synthetic Cells from Giant Unilamellar Vesicles

Lipid films were prepared in 1.5 mL glass vials from solutions of egg-PC in chloroform by drying under N_2 flow and then desiccated for at least 1 hour. Mineral oil was added to a final lipid concentration of 5 mg/mL. The lipid/oil mix was vortexed for 1 minute, heated to 80 °C for 10 minutes, vortexed further for 1 minute, sonicated for 1 hour at 50 °C, stored at room temperature overnight and used the next day. PURExpress components with RNase inhibitor, 200 mM Sucrose, 6 ng/µL bLA-DNA and 25 µM Texas-Red Dextran were added to 200 µL of mineral oil and agitated mechanically along a tube rack to produce a water-in-oil emulsion. The emulsion was pipetted on top of an inverted emulsion column in a 1.5 mL Eppendorf tube consisting of 200 µL outer solution buffer (50 mM HEPES, 400 mM Potassium Glutamate, 200 mM Glucose, pH 7.6) and 100 µL of the same lipid/oil mix on top forming a lipid monolayer interface. The tubes were centrifuged at 16,000 x g for 30 minutes, the oil removed and the GUVs were transferred to a new Eppendorf tube for a wash step with centrifugation at 10,000 x g for 10 minutes. Excess oil and buffer were carefully removed and 30 µL of the GUVs were transferred to a fresh tube and incubated for 5 hours at 37 °C. 25 µL of the GUVs were imaged in Gene Frames on a Leica DMi8 inverted epi-fluorescence microscope.

Preparation of Synthetic Cells from Emulsion Droplets

Cell-free protein synthesis was performed as above using PURExpress containing 25 µM Texas-Red Dextran in a total volume of 3 µL. 5 ng/µL bLA-mNG template or 3 ng/µL each of the uvLA-α (Click-Chemistry-Tools) and bLA-ω templates were used. To the mNG expression was added 2.67 U/µL RNase Inhibitor whereas for split β-Gal, fluorescein-di-β-galactopyranoside (FDG, abcam, ab273643) was added to a concentration of 50 µM. In parallel, a solution of 10 mg/mL of Polystyrene-*block*-poly(ethylene-*ran*-butylene)-*block*-polystyrene (SEBS, average M_w ~118000) in Hexadecane,[9] containing 2wt% Span80 was melted at 50 °C. 30 µL of this solution was added to a 96 well plate with blackened sides and clear bottom (Corning 3603) and left to solidify to coat the bottom of the well. Then, 40 µL of the polymer solution was added to the prepared PURExpress solution, the solutions were quickly emulsified by agitation along a PCR rack and the resulting emulsion transferred to the pre-coated well and left to set for 5 minutes. The plate was then tilted and any ungelled liquid removed. The plate was illuminated through the bottom by flipping the plate (through photomasks if required) and incubated at 37 °C for 3 hours (for split β-Galactosidase) or 4 hours (for mNG) prior to imaging on a Leica DMi8 inverted epi-fluorescence microscope.

Reactions of active esters with amino-modified DNA

Reaction of Amino-modified DNA with 8a

To a 0.5 mL Eppendorf DNA LoBind tube was added 0.5 µL of the DNA (100 µM), 0.5 µL of $NaHCO_3$ (1 M), 15 µL of H_2O and 2.5 µL of a 25 mM Solution of NHS-CouBio in DMF. The reactions were vortexed, spun down in a tabletop centrifuge and placed in a Thermomixer (Eppendorf) overnight, shaking at 600 rpm at room temperature. The resulting solutions were then analysed using denaturing PAGE (16% Acrylamide:Bisacrylamide (19:1), 7M Urea) and compared to the unmodified oligonucleotide.

In all cases, no reaction was observed.

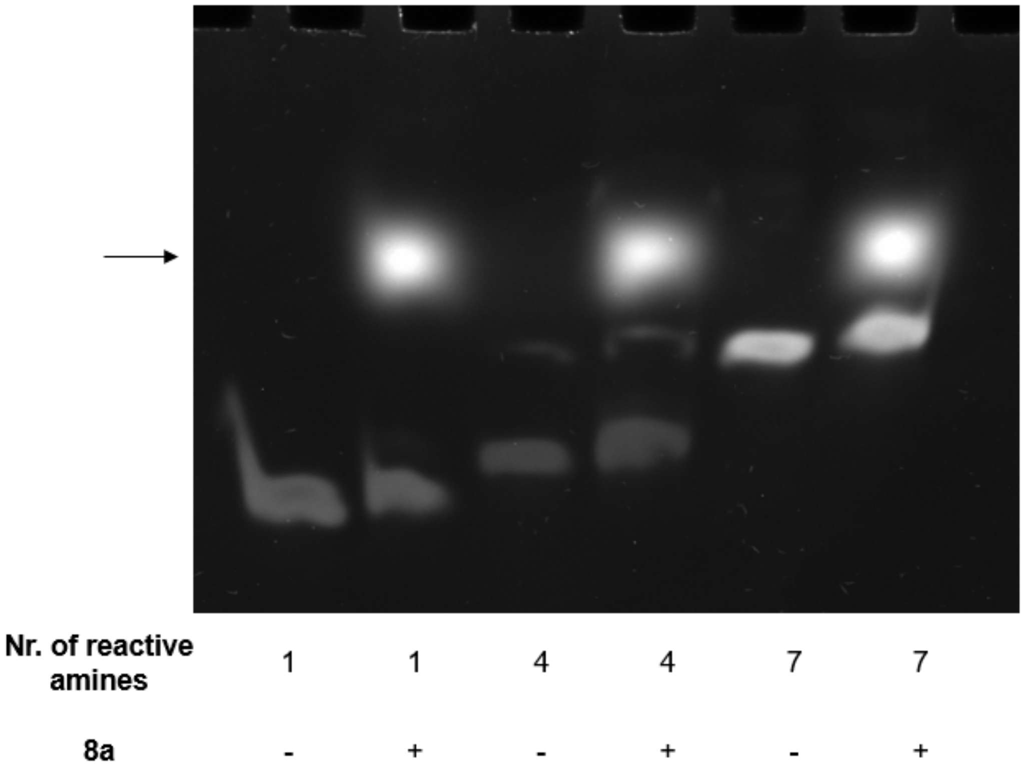

Supplementary Figure 6: Reaction of amino-modified DNAs from Supplementary Table 1 with NHS Coumarin **8a**, analysed by denaturing PAGE. The arrow indicates residual coumarin.

Reaction of 7-amino-DNA with PFP-Coumarin 8b in 100 mM $NaHCO_3$:

Following conditions from Booth *et al.*[5] 10 μM DNA was incubated in 100 mM of $NaHCO_3$ with 12.5 mM of PFP-Coumarin **8b** for 4 hours at room temperature and then 4 degrees overnight in a total volume of 5 μL in 50% and 80% DMF. The samples were then analysed the following day using denaturing PAGE (16% Acrylamide:Bisacrylamide (19:1), 7M Urea) and compared to the unmodified oligonucleotide.

In a separate experiment, the concentration of PFP-Coumarin **8b** was varied from 2.5-10 mM at 50% DMF.

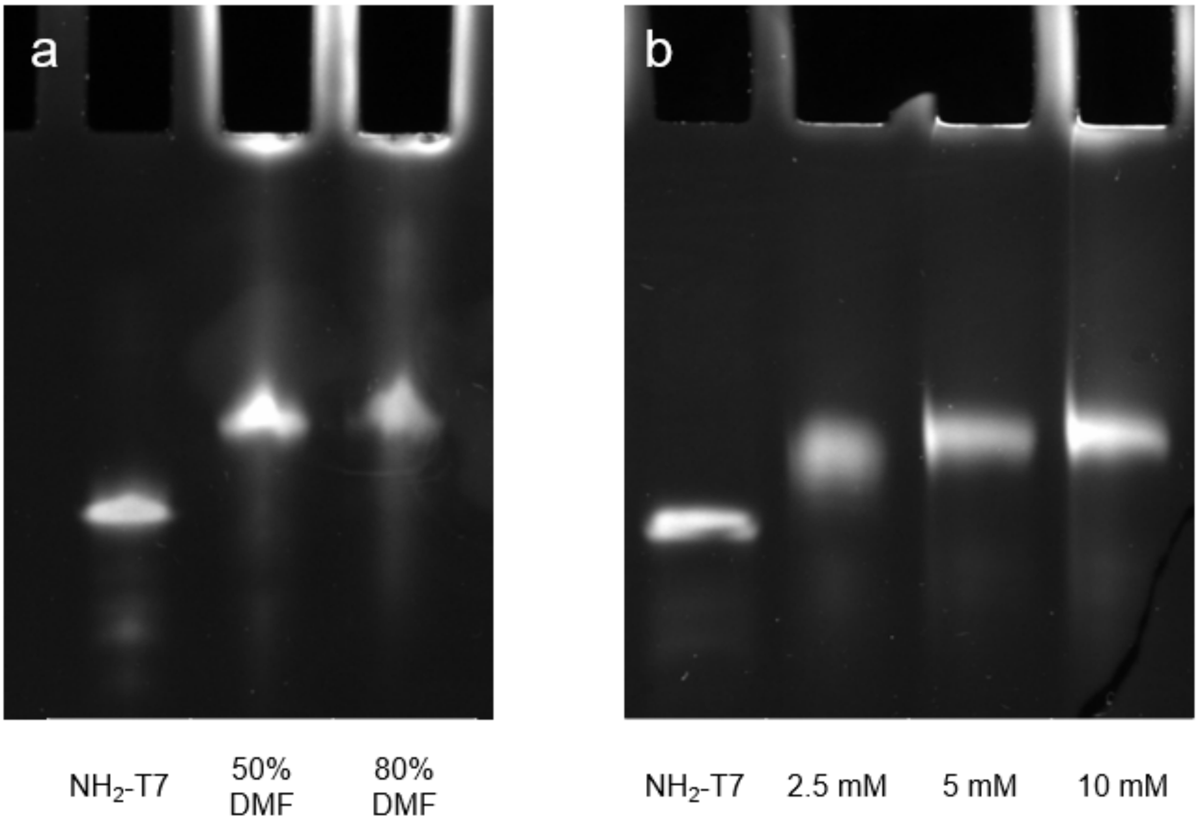

Supplementary Figure 7: Reaction of 7-amine oligonucleotide (**Supplementary Table 1,** Entry 1) with molecule **8b** at different % DMF (**a**) or different [**8b**] (**b**).

HPLC analysis of reaction conditions of 7-amine-T7 with PFP-coumarin:
Reactions were performed on a scale of 1 µL of 7-amine-DNA (**Supplementary Table 1**, Entry 1, 100 µM) in 1.5 mL Eppendorf tubes and all reactions incubated in an Eppendorf Thermomixer at 800 rpm for the required time at the required temperature.

<u>Conditions A</u>: 100 mM $NaHCO_3$, 25 mM PFP-coumarin **8b**, 50% DMF in a total volume of 10 µL, reaction left stirring overnight at room temperature.

<u>Conditions B</u>: 100 mM MOPS pH 8.56, 25 mM PFP-coumarin **8b**, 50% DMF in a total volume of 10 µL at room temperature overnight.

<u>Conditions C</u>: 118 mM MOPS pH 8.56, 12.5 mM PFP-coumarin **8b**, 59% DMF in a total volume of 8.5 µL, heated to 37 °C for 3 hours.

The *-marked peak was identified as the desired, fully modified oligonucleotide through the absorption spectrum recorded by the HPLC, as well as oligonucleotide-LCMS.

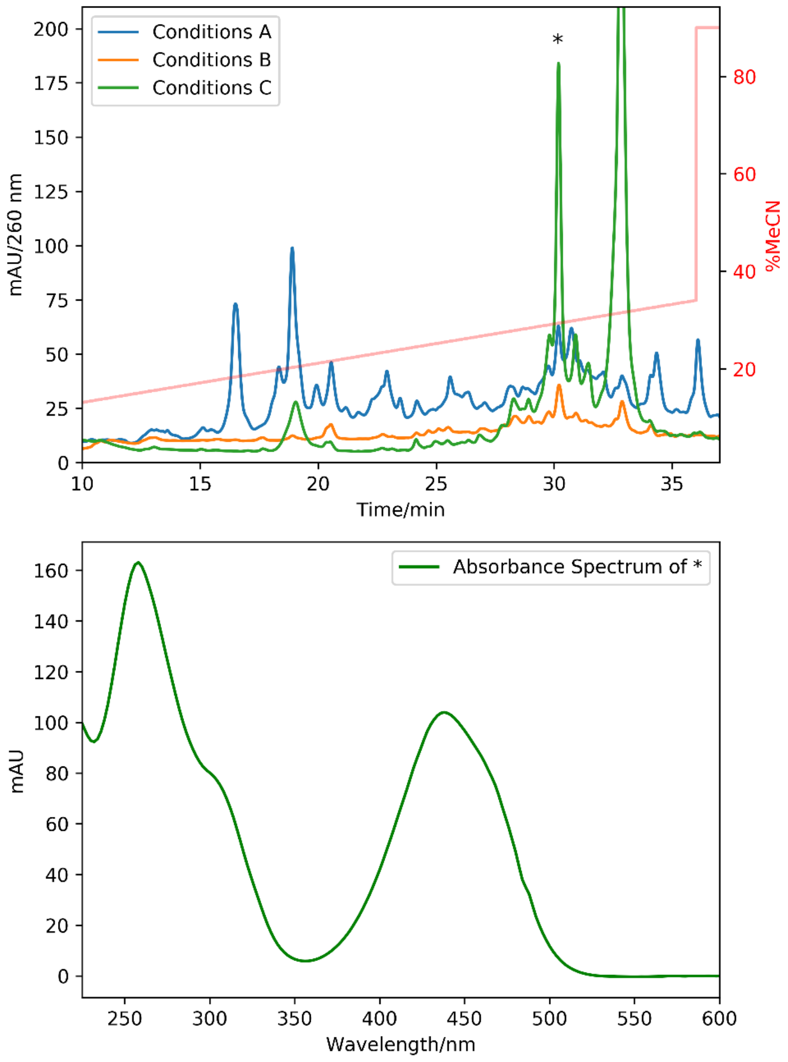

Supplementary Figure 8: Condition Screening for successful modification of 7-amine oligonucleotide (**Supplementary Table 1**, Entry 1). **Top:** HPLC analysis of reaction conditions A-C. **Bottom:** UV/Visible Spectrum of desired, highlighted peak recorded at apex of condition C.

Comparison of PFP- and pNP-Coumarin reactions:

Reactions were performed on a scale of 1 µL of 7-amine-DNA (**Supplementary Table 1**, Entry 1, 100 µM) in 1.5 mL Eppendorf tubes and all reactions incubated in an Eppendorf Thermomixer at 800 rpm for the following conditions C (118 mM MOPS pH 8.56, 12.5 mM of either PFP-coumarin **8c** or *p*NP-coumarin **8b**, 59% DMF in a total volume of 8.5 µL, 37 °C for 3 hours), and analysed by HPLC

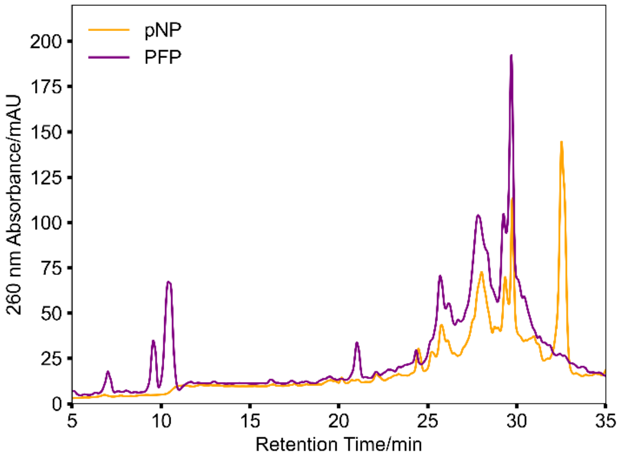

Supplementary Figure 9: Comparison by HPLC of the reaction of 7-amine-containing oligonucleotide (**Supplementary Table 1**, Entry 1) with PFP/pNP-Coumarin **8b/c**.

HPLC Traces of light-activatable Primers

UV-activatable T7 Primer (Click-Chemistry-Tools)

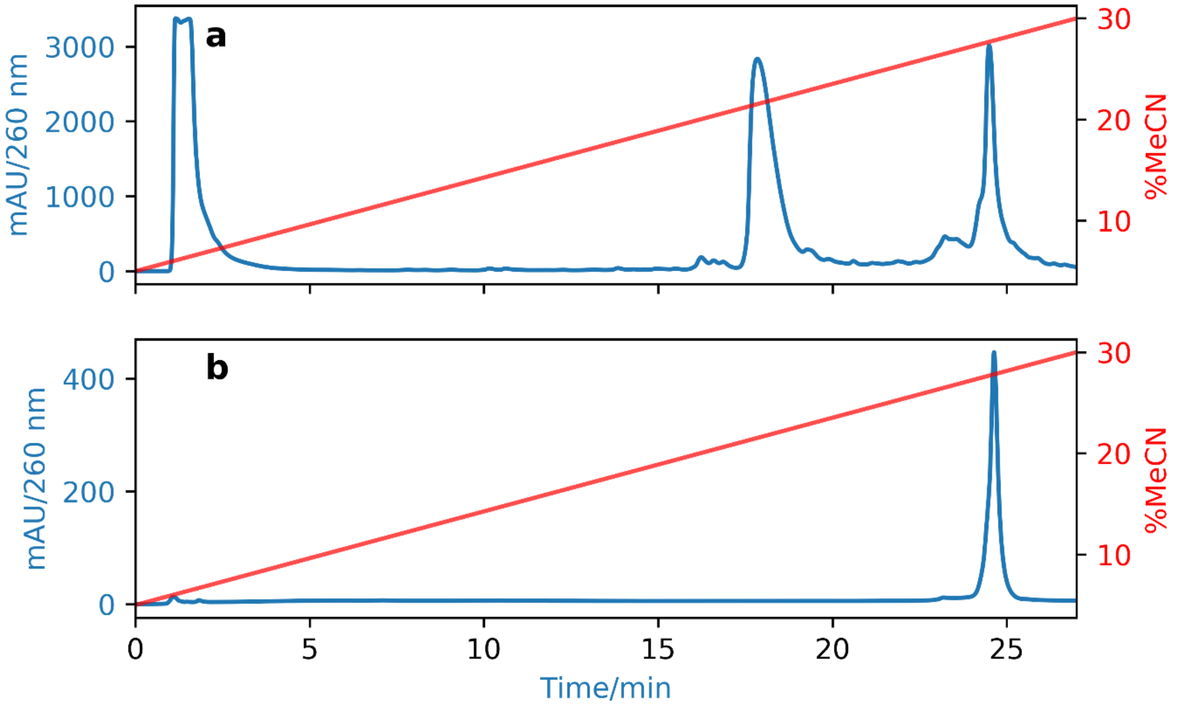

Supplementary Figure 10: HPLC trace of the purification of uvLA-T7 primer using the Click-Chemistry Tools molecule, pre- (**a**) and post-(**b**) purification.

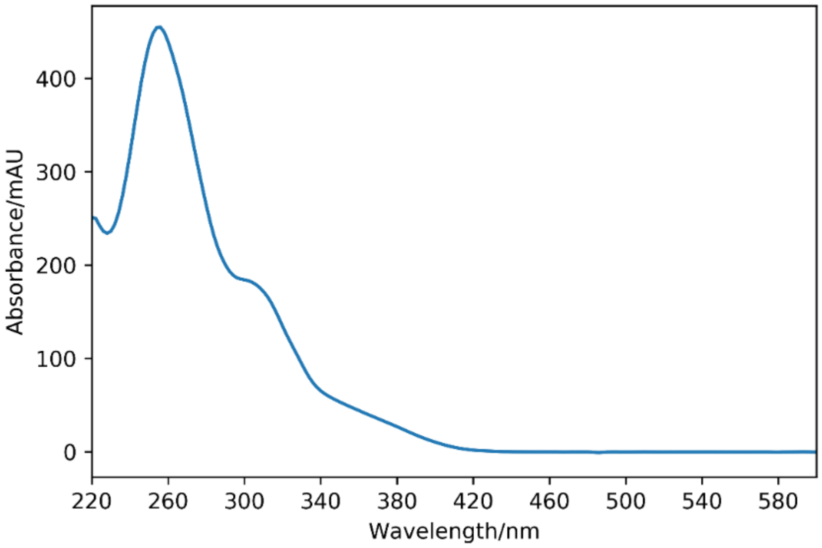

Supplementary Figure 11: UV/Vis trace of uvLA-T7 primer made using the PC-Biotin NHS from Click-Chemistry Tools, as recorded by HPLC.

UV-activatable T7 Primer (AmberGen)

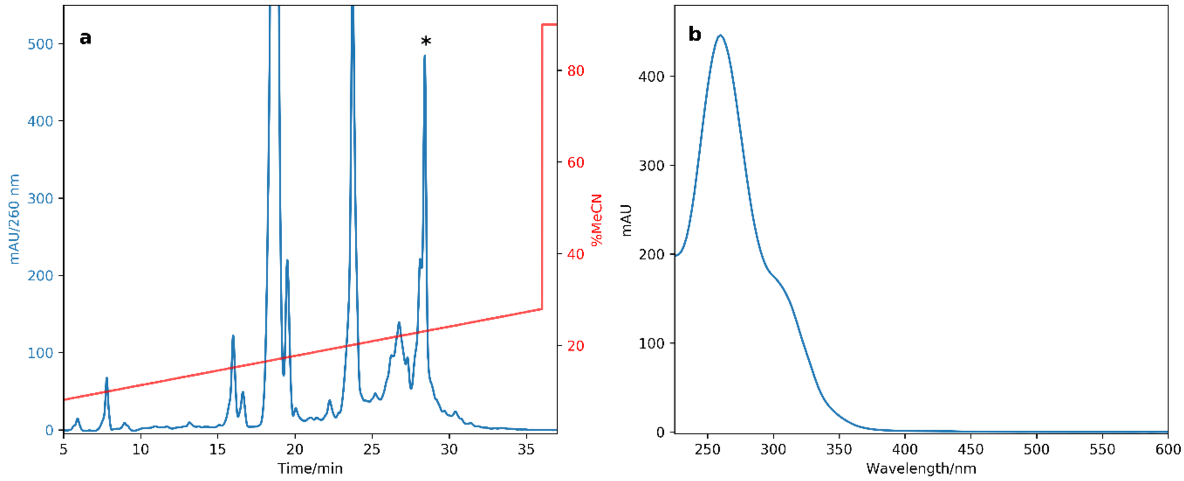

Supplementary Figure 12: **a**, Crude HPLC of the reaction of AmberGen PC-Biotin-NHS with 7-amine containing oligonucleotide (**Supplementary Table 1**, Entry 1). Desired product is highlighted with a *. **b**, UV/Vis trace of the highlighted peak as recorded by HPLC.

Blue light-activatable Primer bLA-T7

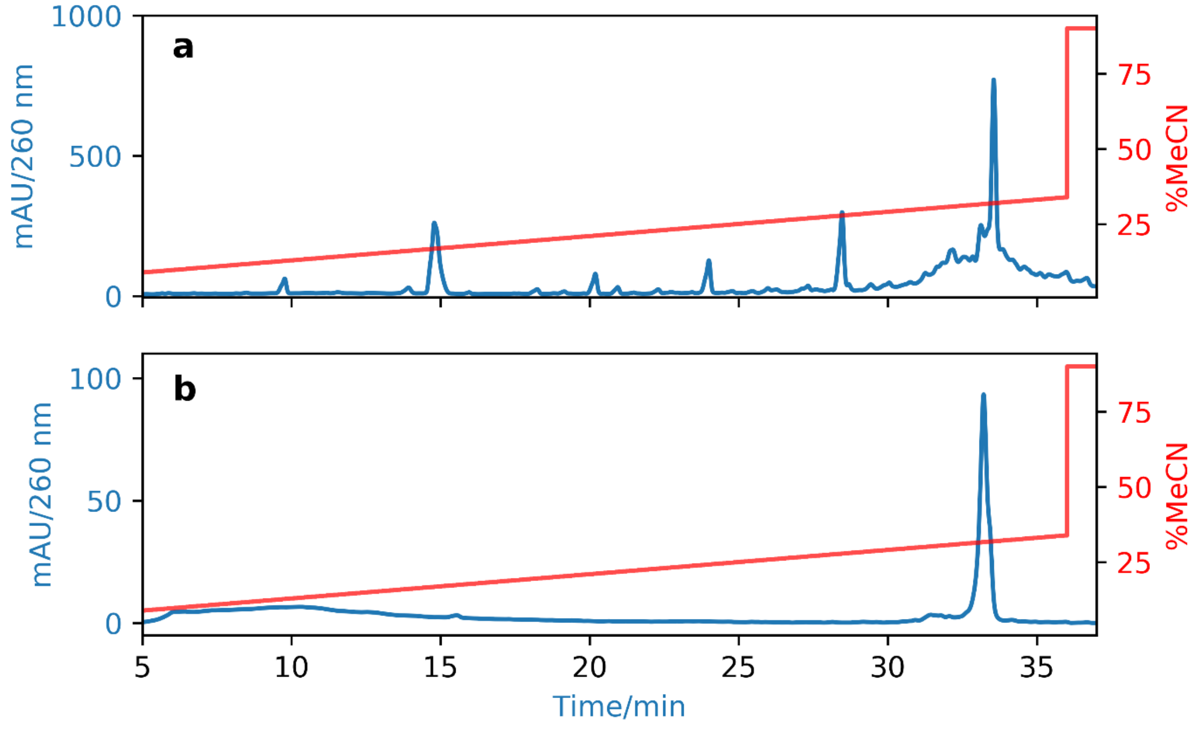

Supplementary Figure 13: Purification of bLA-T7 on HPLC. **a**, Crude HPLC trace of reaction of PFP-Carbonate **8b** with 7-amine containing oligonucleotide (**Supplementary Table 1**, Entry 1). **b,** HPLC trace of purified bLA-T7.

Supplementary Data

PAGE of amino-T7 and bLA-T7

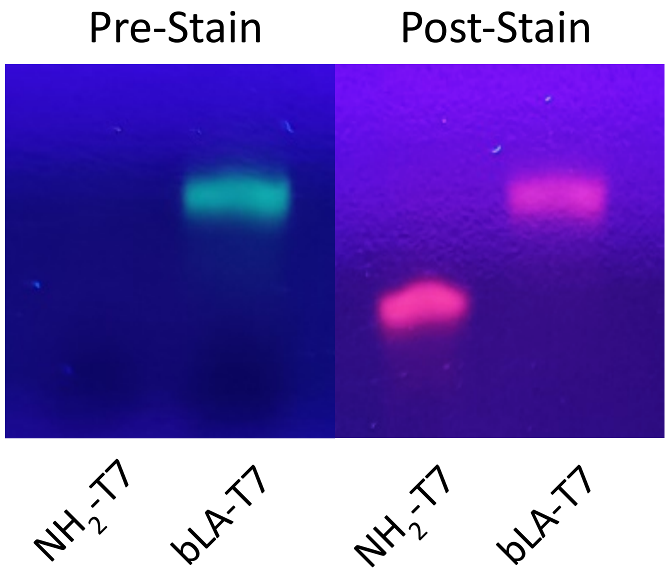

Supplementary Figure 14: Denaturing PAGE (16%) analysis of the 7-amino- and blue light-activated T7 ssDNA pre- and post-staining with GelRed. The green fluorescence of the ssDNA is due to the attached coumarin photocage.

Expression of Broccoli-aptamer from annealed DNA Templates

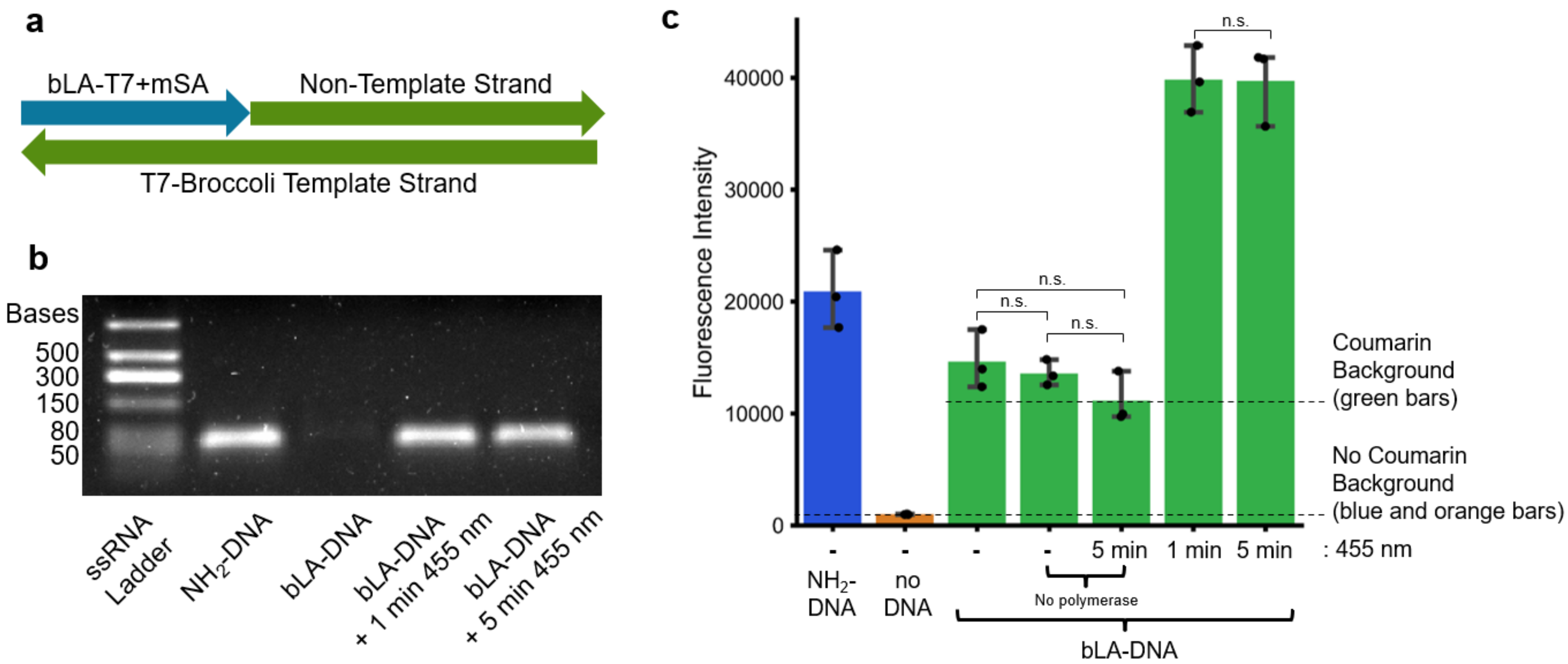

Supplementary Figure 15: Expression of the broccoli RNA aptamer from a caged bLA-DNA template. **a**, Schematic of the broccoli aptamer template DNA from bLA-T7, annealed from bLA-T7, T7-Broccoli Template Strand and Non-Template Strand (**Supplementary Table 3**). **b**, Agarose gel electrophoresis of transcribed RNA from NH_2- or bLA-DNA Broccoli templates, with or without 455 nm illumination. In the absence of light, the bLA-DNA did not show any appreciable amount of RNA (1% band intensity vs NH_2-DNA control), whereas upon illumination for 1 and 5 minutes, comparable amounts of RNA to the NH_2-control were produced (93% and 90% band intensity for 1 and 5 minutes illumination respectively vs. NH_2-DNA control). **c**, Fluorescence measurements of the *in vitro* transcription of broccoli aptamer in presence of DFHBI. A no polymerase control demonstrated the DNA itself showed a strong fluorescence background from the attached coumarin photocage. Taking this into account, tight control of fluorescence was observed, followed by a full recovery of expression with 455 nm illumination. n.s. – non-significant, i.e. p-value > 0.05

AGE of bLA-mV DNA at different Illumination Times

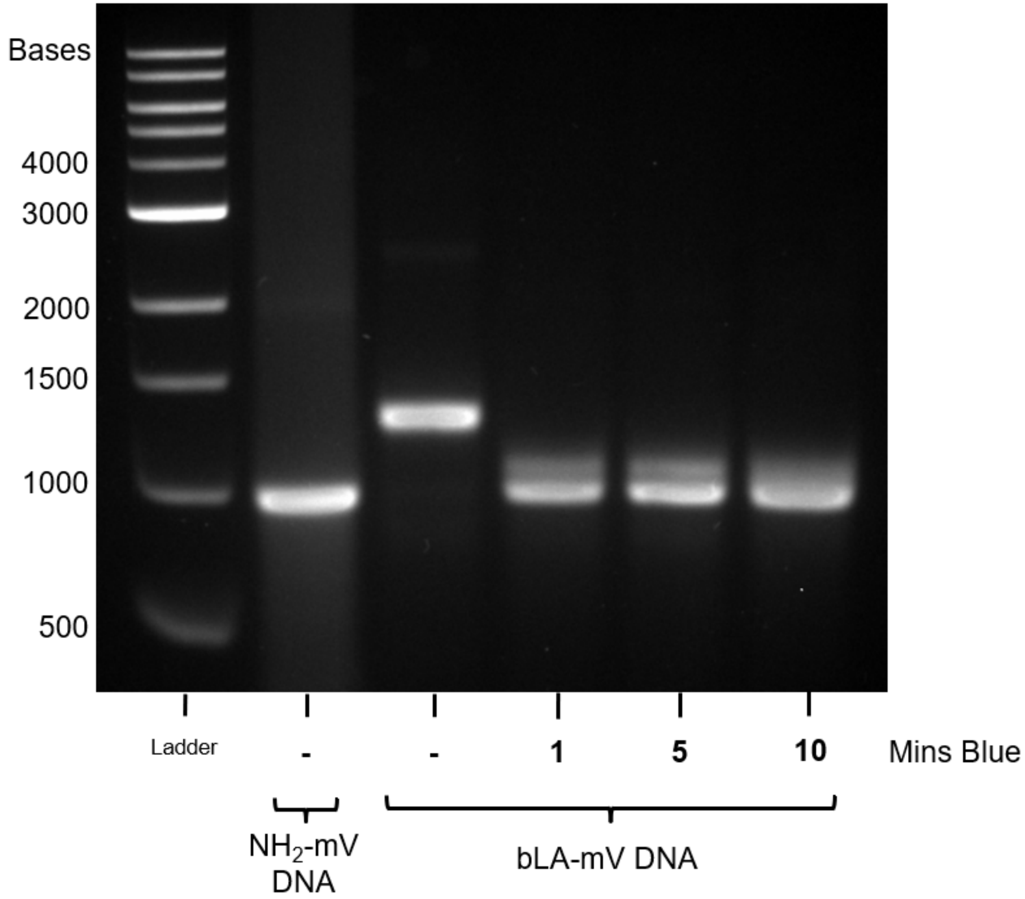

Supplementary Figure 16: 1% AGE of bLA-mV DNA with different durations of blue light illumination.

UV-Visible Absorbance of LA-T7 primers and spectra of the LEDs employed

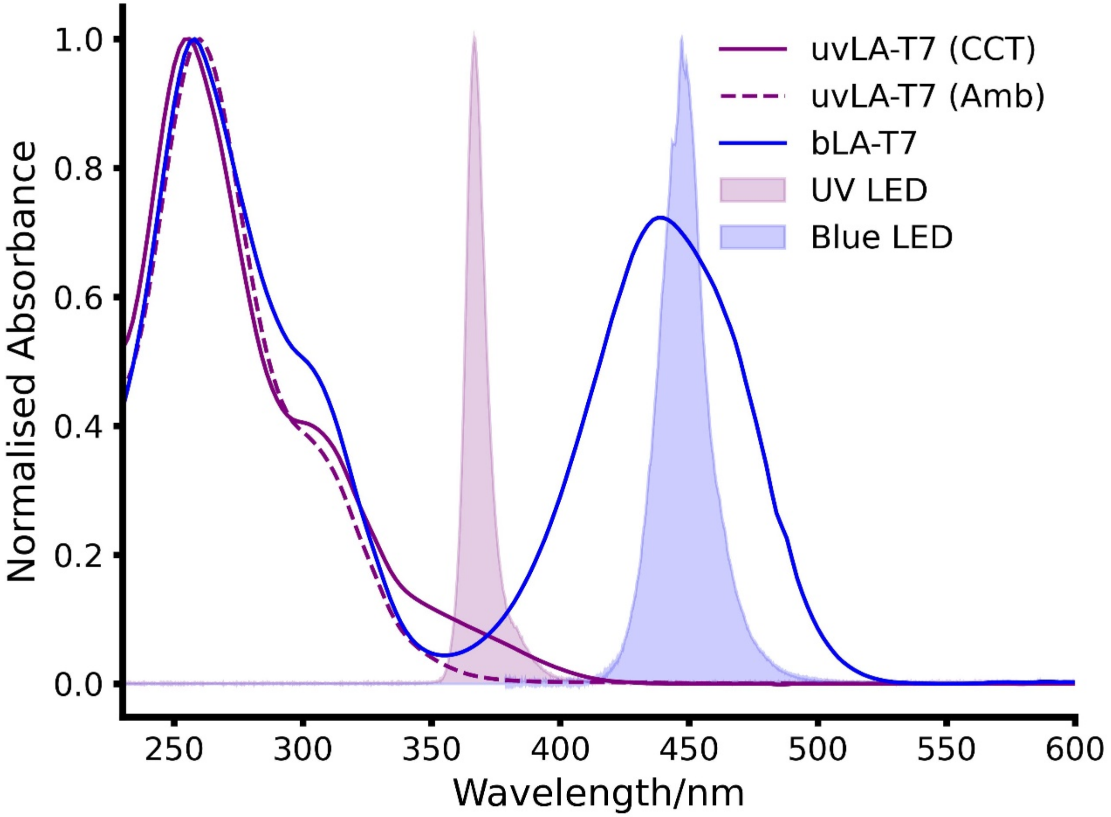

Supplementary Figure 17: UV-visible absorbance of LA-T7 ssDNA modified with the 2-nitrobenzyl-based and coumarin-based biotinylated photocages and the spectra of the LEDs used for their photocleavage. Absorbance data was normalised to the peak at 260 nm. LED spectra were obtained from ThorLabs. CCT refers to the UV-photocleavable biotin obtained from Click-Chemistry-Tools. Amb refers to the UV-photocleavable biotin obtained from AmberGen.

Comparison of unmodified and amino-C6-dT-modified DNA templates in cell-free protein synthesis

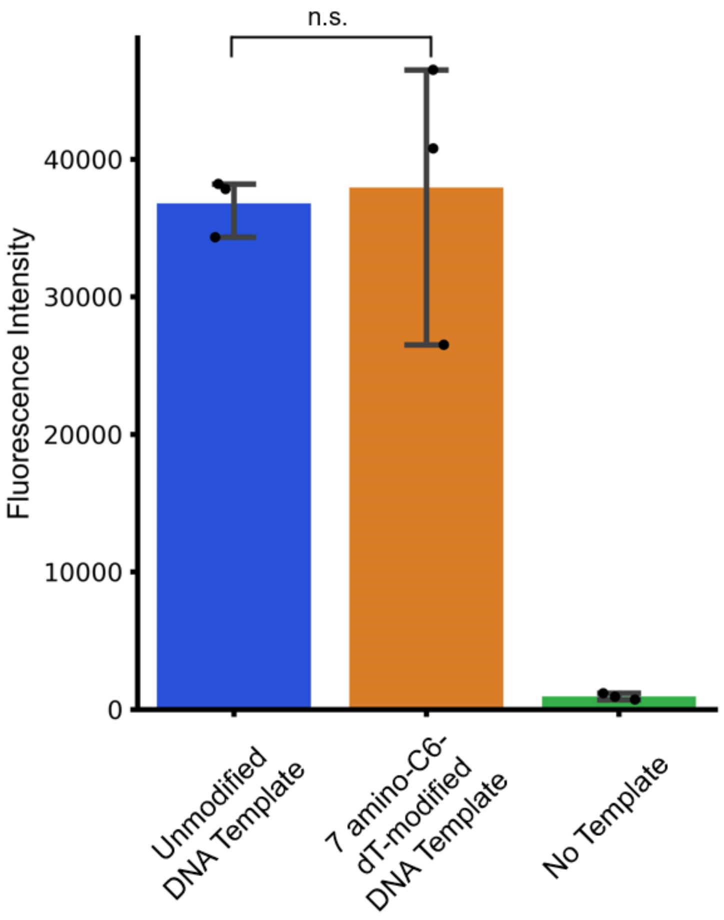

Supplementary Figure 18: Comparison between unmodified, and 7x amino-C6-dT-modified linear DNA templates encoding for mVenus used in cell-free protein synthesis. No significant difference between the unmodified and modified templates was observed after 4 hours of expression (103% vs. unmodified control, not significant, p-value = 0.85).

***In Vitro* Transcription of mVenus**

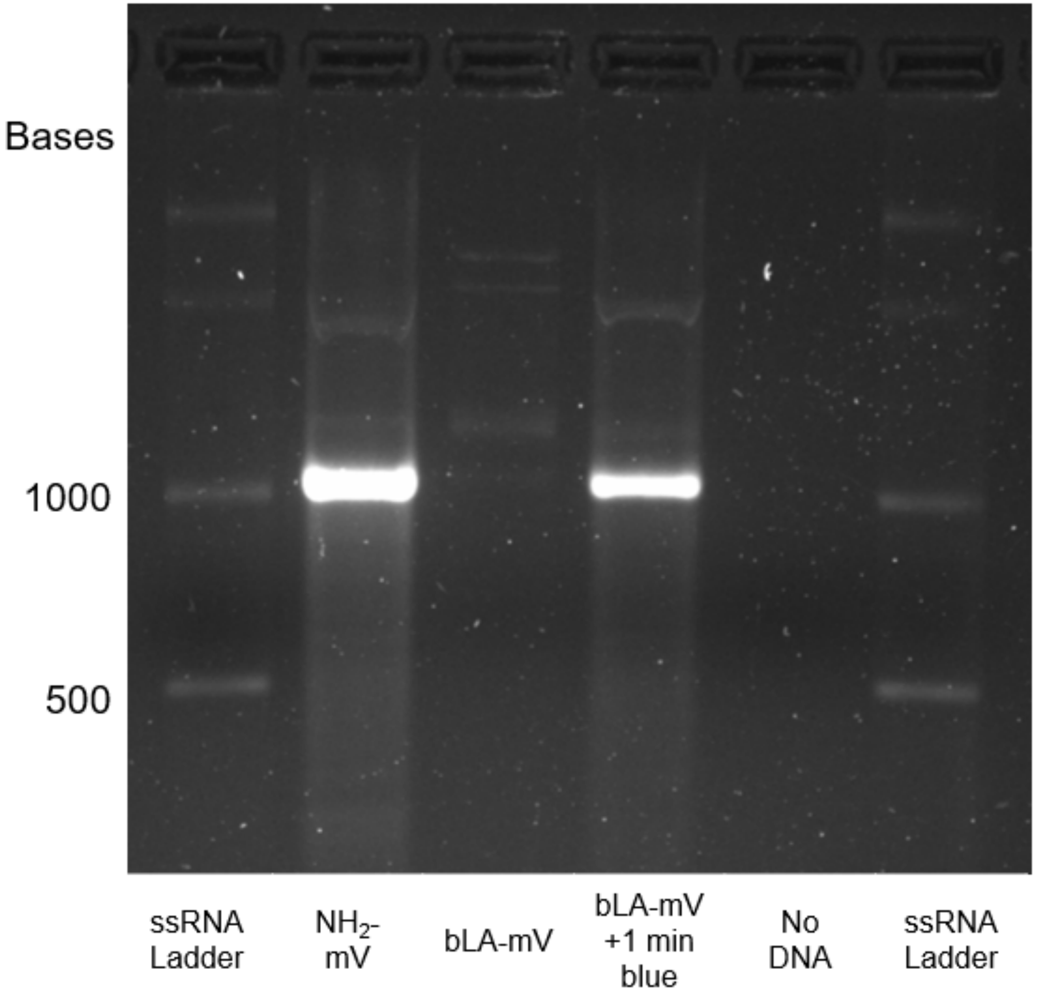

Supplementary Figure 19: *In vitro* transcription of bLA-mV DNA with or without light, analysed by agarose gel electrophoresis.

Light Titration for the expression of bLA-mV DNA in CFE

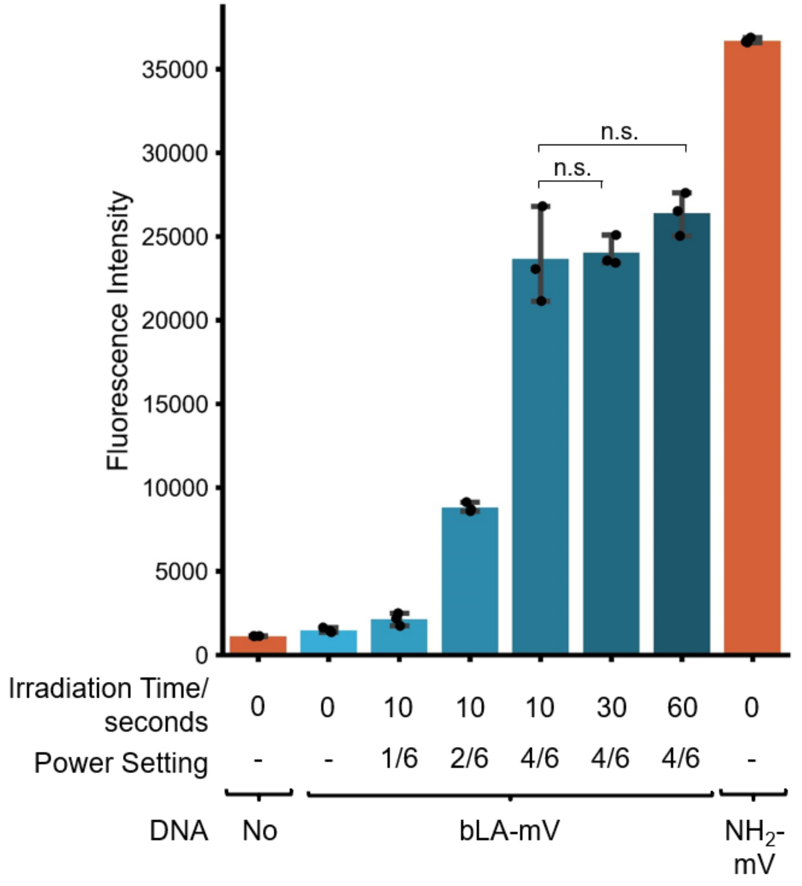

Supplementary Figure 20: Different amounts of irradiation to express mVenus protein from bLA-mV DNA in CFE. A tight off state of the bLA-mV DNA of only 1% activity vs. amino-DNA compared to the background is seen. Rapid activation at high LED power is observed, with 10 seconds at 4/6 power giving 63% activity vs amino-DNA. Increase in time does not lead to a large increase in expression. This is likely due to photobleaching of the photocage, as is also visible by a stalling of photorelease of DNA in **Supplementary Figure 16**. n.s. – non-significant, i.e. p-value >0.05

AGE of split β-Galactosidase Vectors and Inserts

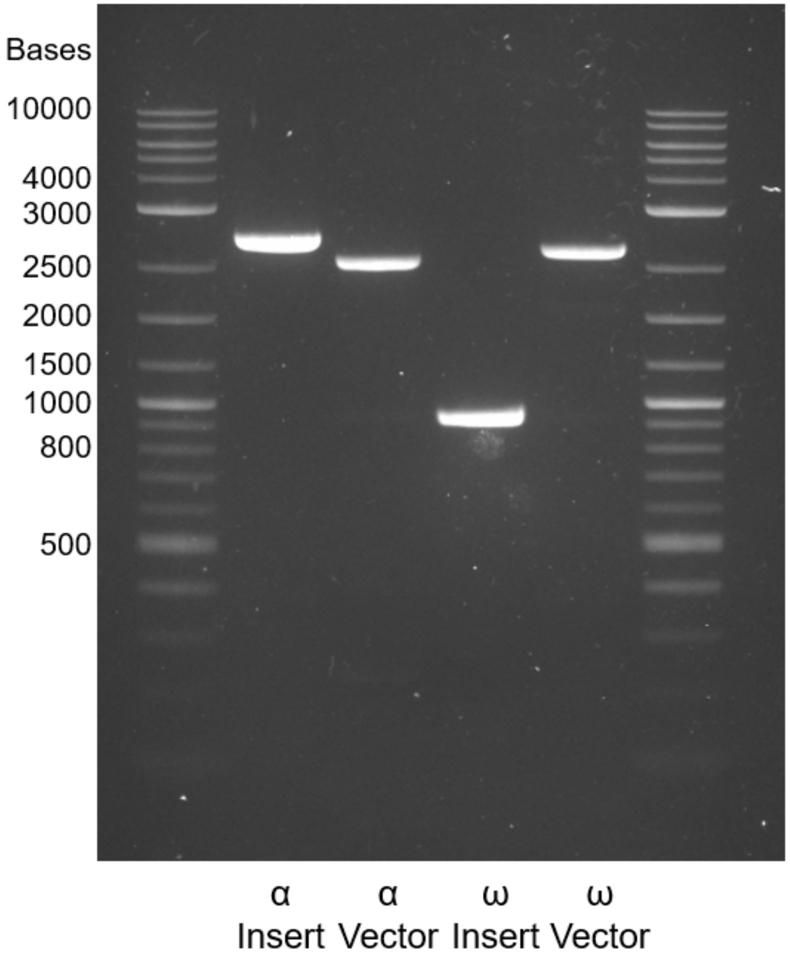

Supplementary Figure 21: Agarose gel of PCR fragments (insert and vector) for α- and ω-subunits ran against 1kb Plus Ladders (NEB).

AGE of α- and ω-subunit DNA

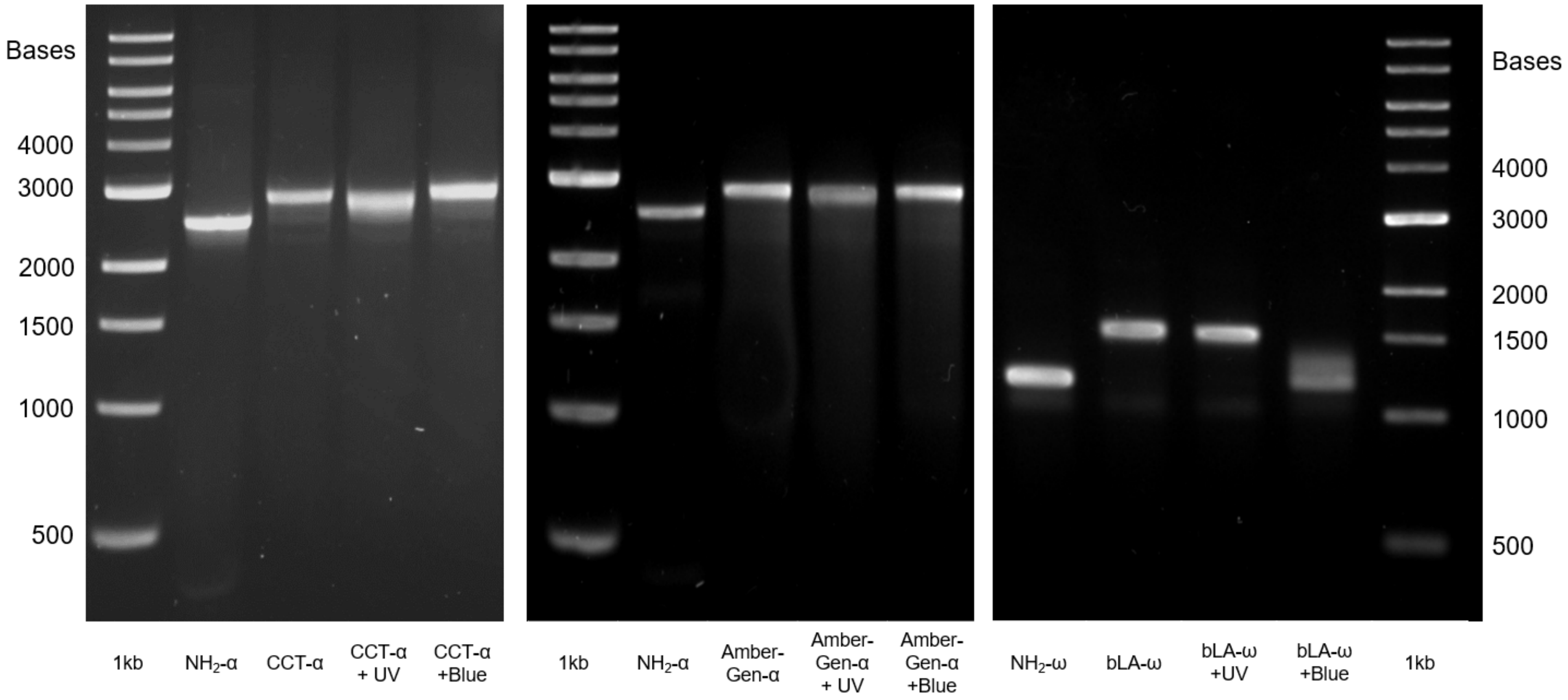

Supplementary Figure 22: AGE of amino- and modified α- and ω-subunit DNA with 50x excess monovalent streptavidin. CCT = Click-Chemistry-Tools

In vitro Transcription of Alpha- and Omega-Subunits of β-Galactosidase

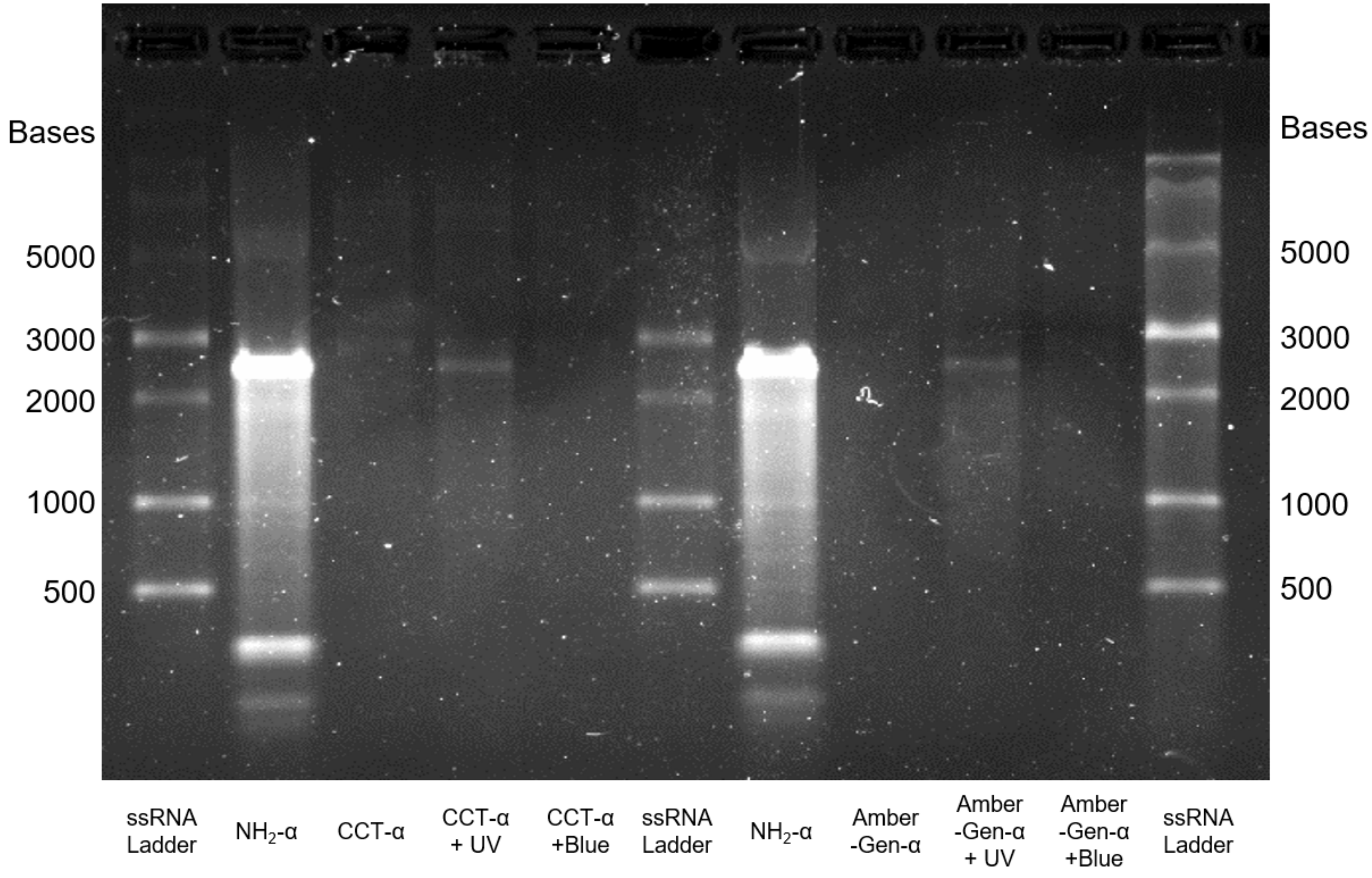

Supplementary Figure 23: *In vitro* transcription of uvLA-α DNA using two different UV-activatable molecules without or with UV/Blue light, analysed by agarose gel electrophoresis (2%). CCT = Click-Chemistry-Tools

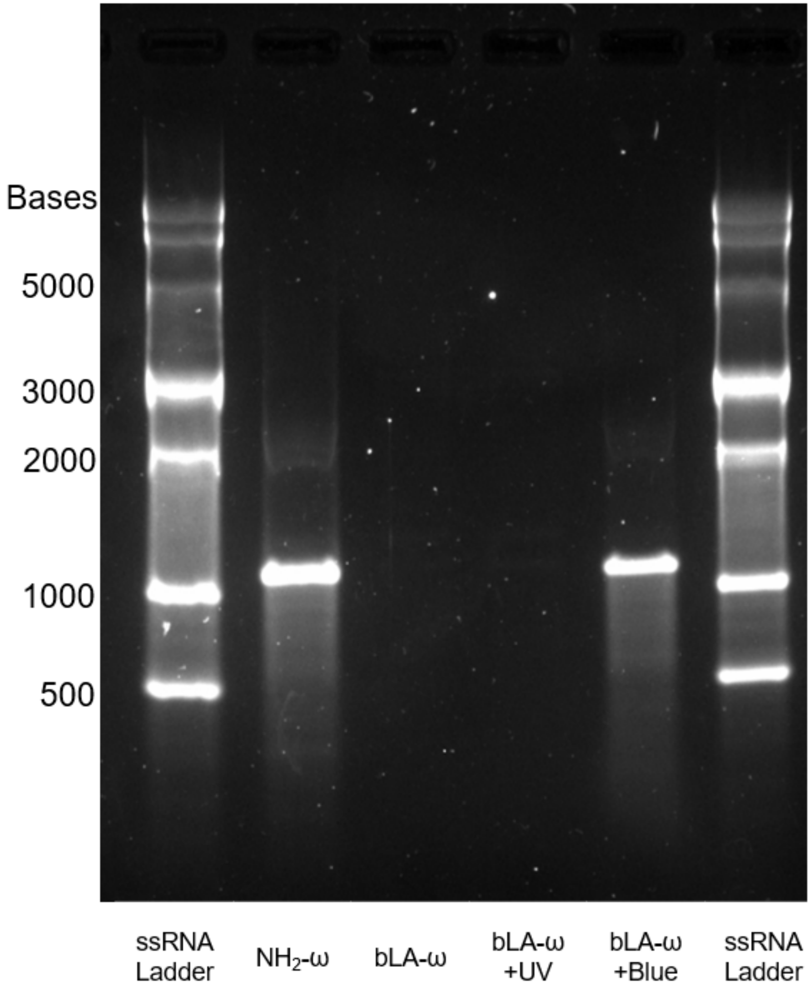

Supplementary Figure 24: *In vitro* transcription of bLA-ω DNA without or with UV/Blue light, analysed by AGE.

***In vitro* transcription/translation of split β-Galactosidase**

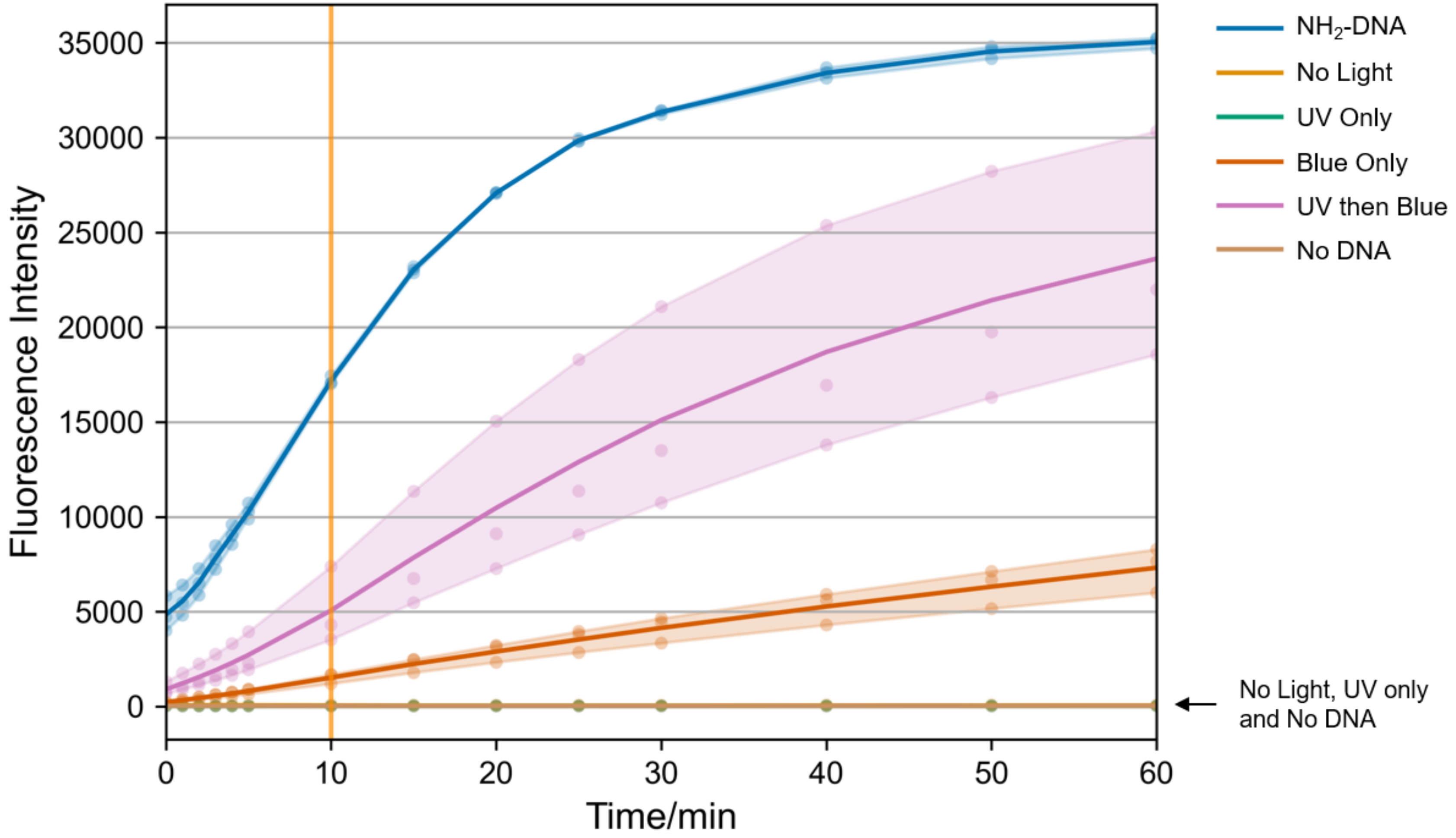

Supplementary Figure 25: Evolution of fluorescence over time using the uvLA-α Subunit DNA (prepared using the molecule from Click-Chemistry-Tools) in the AND gate. No Light, No DNA and UV-Only are overlapping at the baseline. Orange, horizontal line at minute 10 indicates data plotted in **Supplementary Figure 26**.

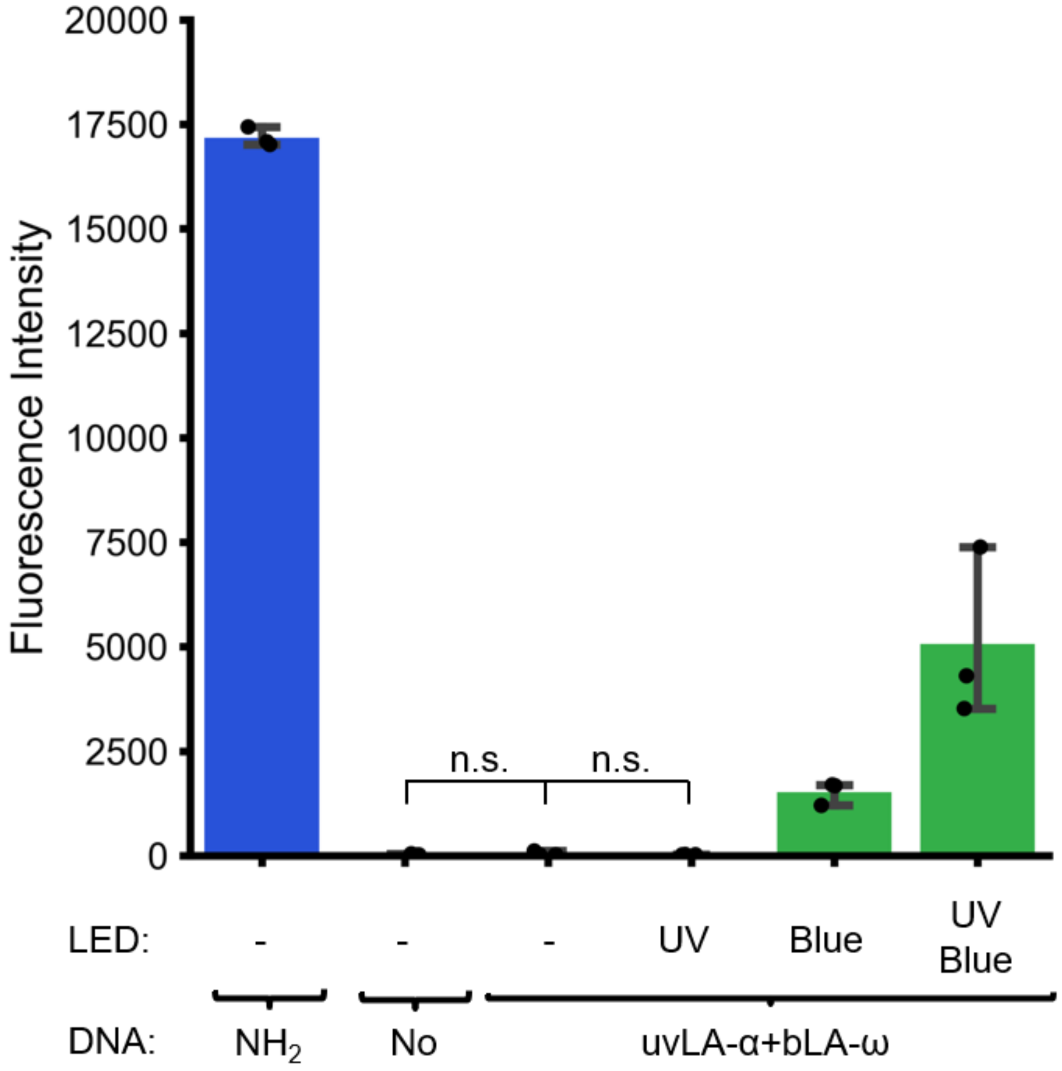

Supplementary Figure 26: Fluorescence of UCA at minute 10 (**Supplementary Figure 25,** orange line), produced via the AND-gate using the uvLA-α prepared with the molecule from Click-Chemistry-Tools. n.s. – non-significant, i.e. p-value > 0.05.

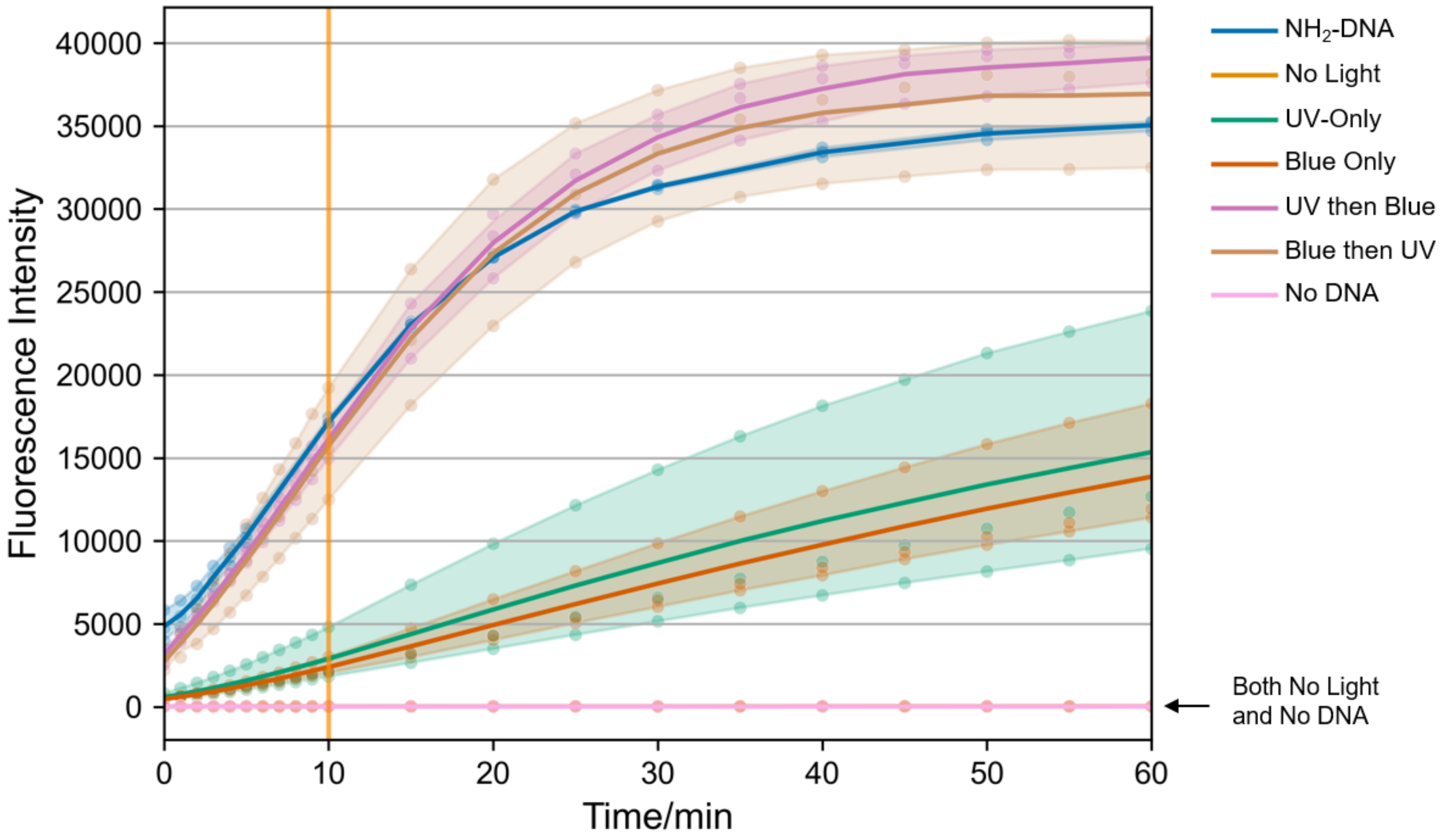

Supplementary Figure 27: Evolution of Fluorescence over Time using the uvLA-α subunit DNA prepared with the molecule from AmberGen in the AND gate compared to the unmodified NH_2-DNA (same as **Supplementary Figure 25**). No Light and No DNA are overlapping at the baseline. Orange, horizontal line at minute 10 indicates data plotted in **Figure 5c.**

β-Galactosidase Calibration Curve

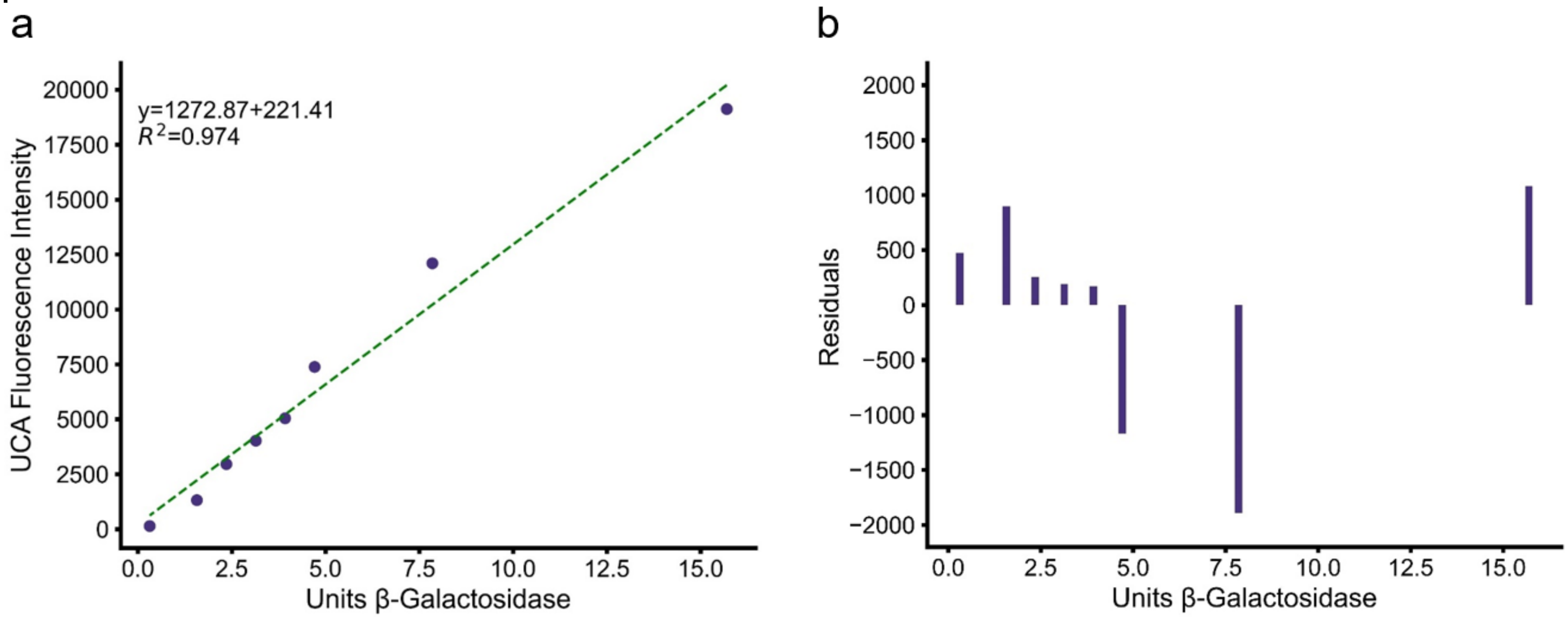

Supplementary Figure 28: Calibration curve (**a**) and residuals (**b**) of commercial β-Galactosidase added to CUG and measured after 10 minutes of incubation at 37 °C. Amount of observed fluorescence from the hydrolysis product umbelliferyl-3-carboxylic acid after 10 minutes is linear to the amount of β-Galactosidase in Units added to the solution. This data was used to quantify the amount of β-Galactosidase produced in **Supplementary Figure 27**.

Calibration Curve: Slope: 1272.87. Intercept: 221.41. p-value: $5.56*10^{-06}$. Standard Error: 84.94

Residuals: Mean: 0.01. Standard Error: 360.36. Median: 220.62. Sum: 0.11. Confidence Interval (95%): 852.12.

Quantification of produced β-Galactosidase in Supplementary Figure 27

Index	Experiment	Mean	Units β-Gal added	U/µL produced
1	NH_2-DNA	17180	13.32	6.66
2	LA-DNA	33.67	-0.15	-0.07
3	LA-DNA + UV	2905	2.11	1.05
4	LA-DNA + Blue	2405.67	1.72	0.86
5	LA-DNA + UV/Blue	16135.33	12.50	6.25
6	LA-DNA + Blue/UV	15746	12.20	6.10
7	No DNA	22	-0.16	-0.08
		Standard Error: 84.94	Standard Error: 0.11	Standard Error: 0.06

Supplementary Table 4: Quantification of produced split β-Galactosidase in **Figure 4c** and **Supplementary Figure 27** *via* calibration curve of intact, commercial β-Galactosidase in **Supplementary Figure 28**.

AGE of NH_2- and bLA-mNG DNA

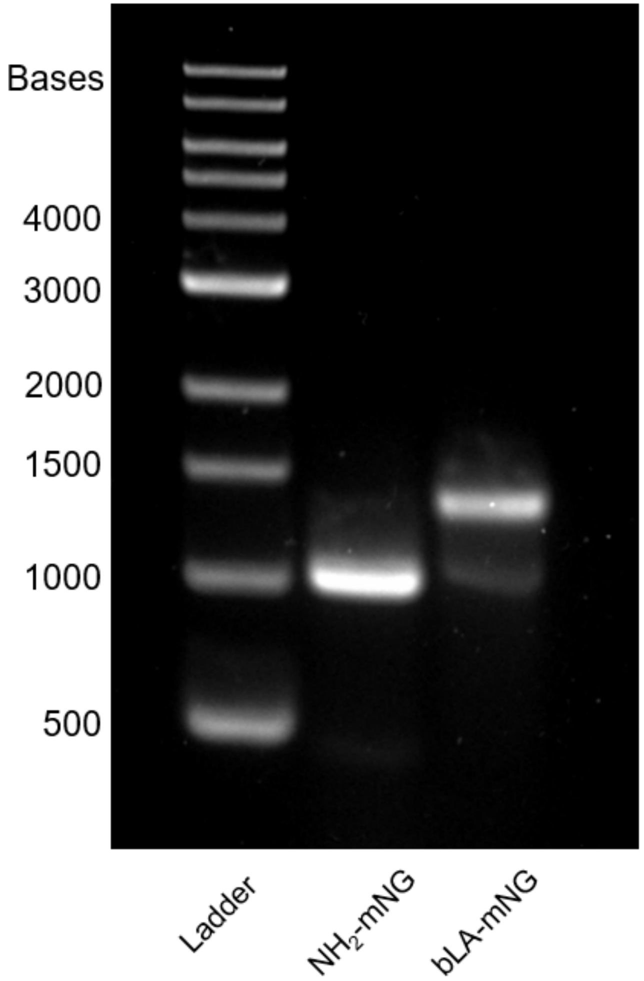

Supplementary Figure 29: AGE (1%) of Amino- and bLA-mNeonGreen DNA from PCR with 50x molar excess monovalent streptavidin

Control of Gene Expression inside Giant Unilamellar Vesicle-based Synthetic Cells

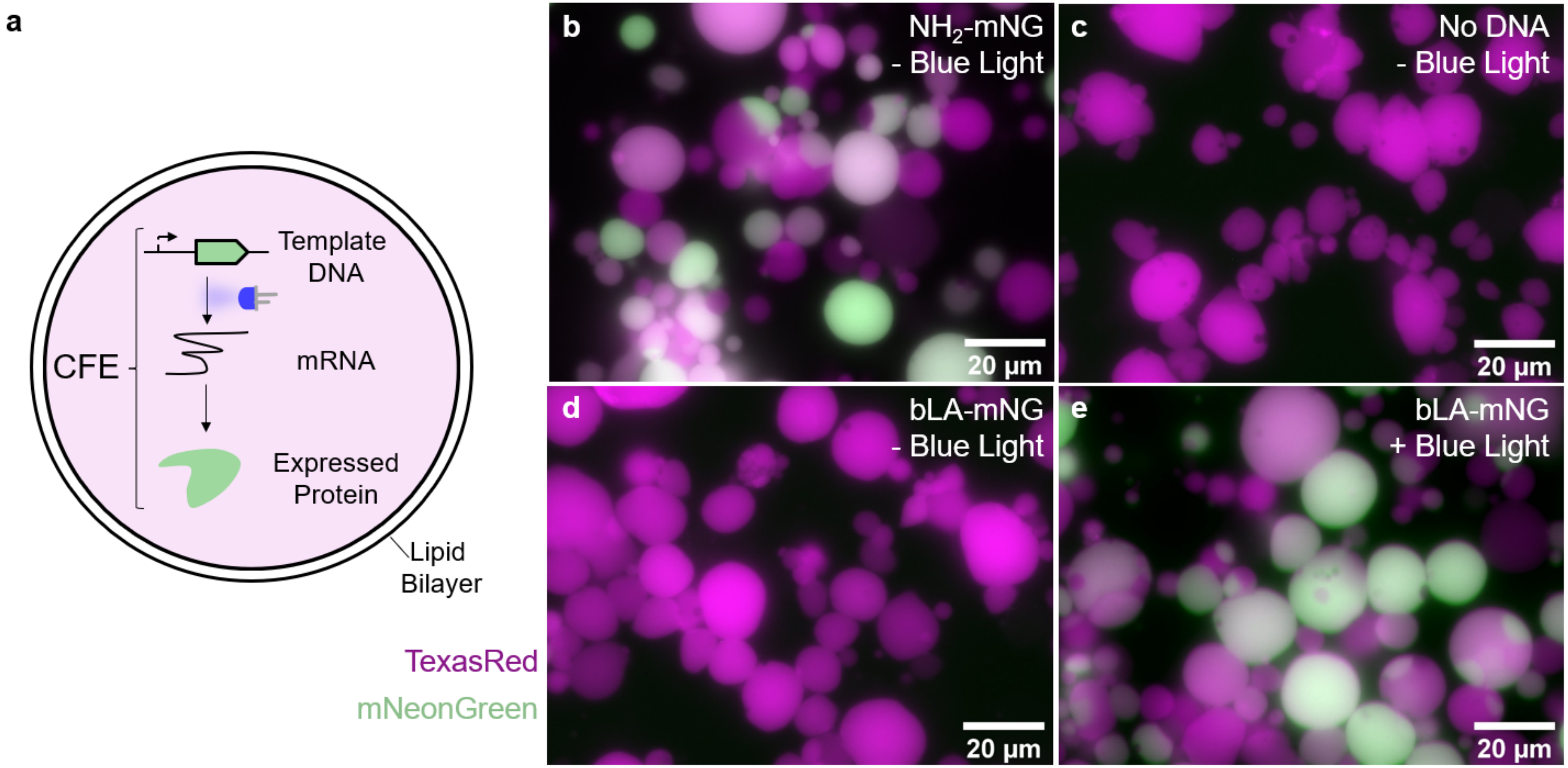

Supplementary Figure 30: Blue light-activated expression within giant unilamellar vesicle (GUV) synthetic cells. **a**, Schematic of a GUV synthetic cell. A lipid bilayer encapsulates a cell-free expression system and a template DNA, from which RNA and protein are expressed *in situ*. **b**, When NH_2-mNG DNA in the absence of blue light was included in the synthetic cells high fluorescence was observed. **c**, When no DNA was added within the synthetic cells in the absence of blue light no fluorescence was observed. **d-e**, When encapsulating bLA-mNG DNA inside synthetic cells, fluorescence was not observed in the absence of light (**d**), however, following 1 minute of 455 nm illumination, fluorescence was observed to a similar level as **b** (**e**). Scale bar = 20 µm.

NH_2-mNG Emulsion Droplets

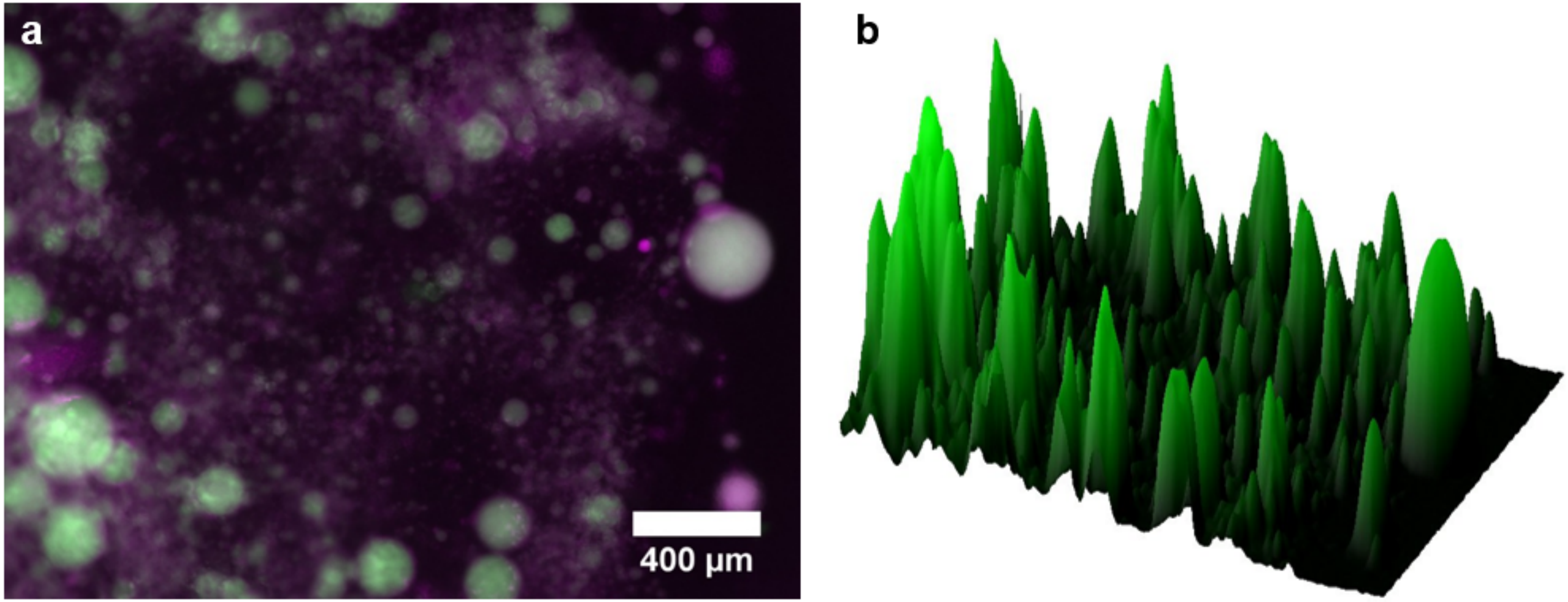

Supplementary Figure 31: Representative composite image (**a**) and its corresponding surface plot (**b**) for the GFP-channel of amine-modified mNG DNA being expressed inside emulsion droplets.

Additional Images of two-wavelength-controlled AND-gate inside Emulsion droplets

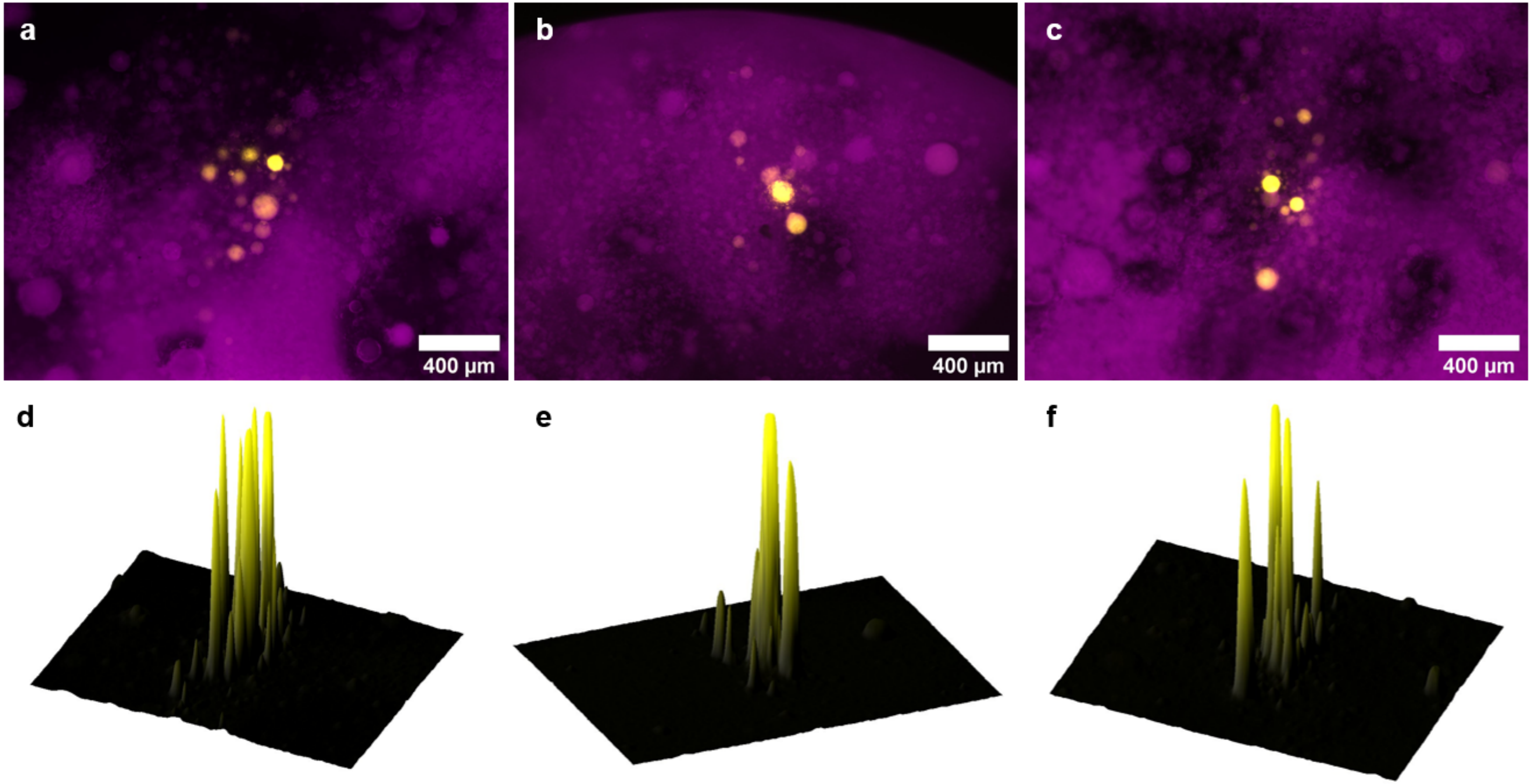

Supplementary Figure 32: Additional repeats of the 2-wavelength controlled activation of emulsion droplets using the split β-Galactosidase AND-gate composed of the uvLA-α DNA (prepared with the molecule from Click-Chemistry-Tools) and bLA-ω DNA, showing hydrolysis of FDG in the presence of active enzyme, producing fluorescein, only at the intersection of the two applied line-shaped photomasks. **a-c:** Composite images of TxRd- and GFP-Channels. **d-f**: 3D intensity profiles of GFP channel.

NH_2-α and -ω-containing Emulsion Droplets

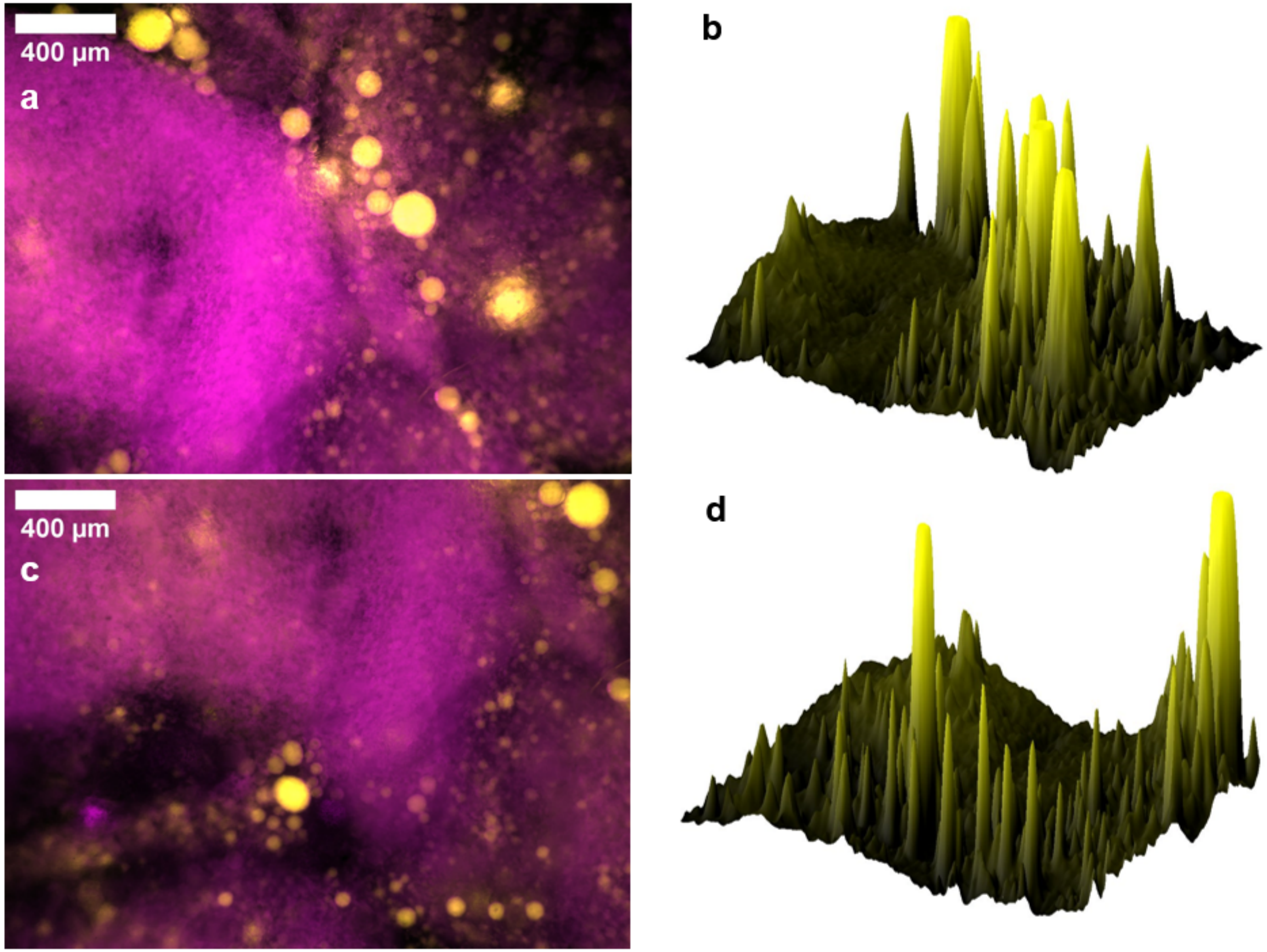

Supplementary Figure 33: Representative example of the amine-DNA split β-Galactosidase inside emulsion droplets. Images were taken from the same well at different positions.

Oligonucleotide Mass Spectra

Oligonucleotide Mass Spectrometry

Oligonucleotide Mass Spectra were recorded on a Waters Xevo G2 QTOF ESI- UPLC-MS system equipped with a Waters ACQUITY UPLC BEH C18 column (130 Å, 1.7 µm, 2.1 mm x 50 mm). A gradient of MeOH in Et_3N and hexafluoroisopropanol (HFIP) was used (buffer A, 8.6 mM Et_3N, 200 mM HFIP in 5% $MeOH/H_2O$ (v/v); buffer B, 20% buffer A in MeOH).[10] For nitrobenzyl-modified DNA, samples were analysed using a gradient of 0-70% buffer B over 8 minutes. For coumarin-modified DNA, samples were analysed using a gradient of 50-100% Buffer B over 8 minutes. Data was then deconvoluted using MassLynx v4.1.

Entry	Oligonucleotide	Mass after Deconvolution	Expected Mass
1	uvLA-T7 (Click-Chemistry-Tools)	15399	15400
2	uvLA-T7 (AmberGen)	15066	15064
3	bLA-T7	15217	15218

Supplementary Table 5: Modified oligonucleotides and their masses after deconvolution as measured by LC-MS.

uvLA-T7 (Click-Chemistry-Tools)

LC-UV and TIC

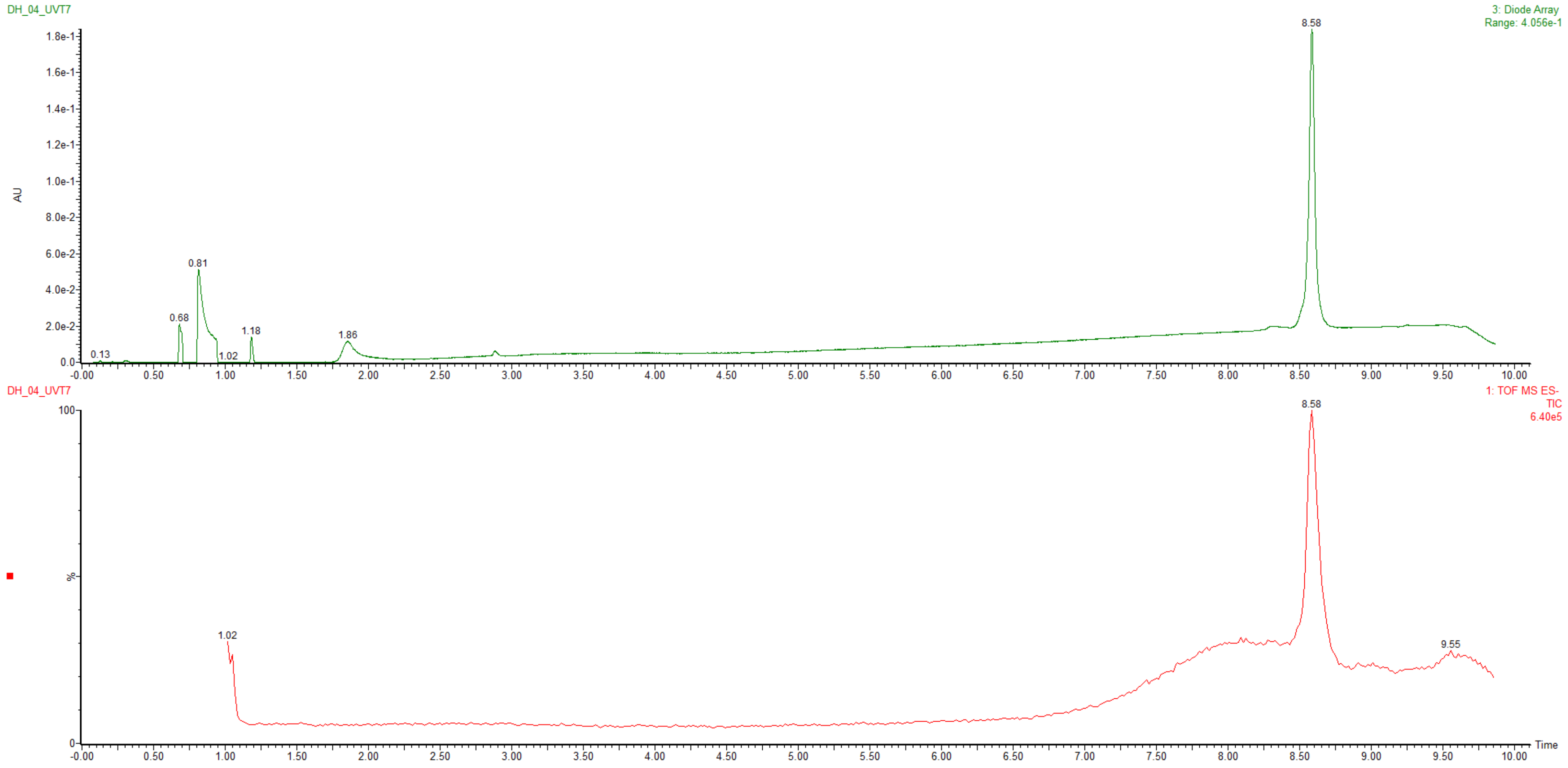

Mass After Deconvolution:

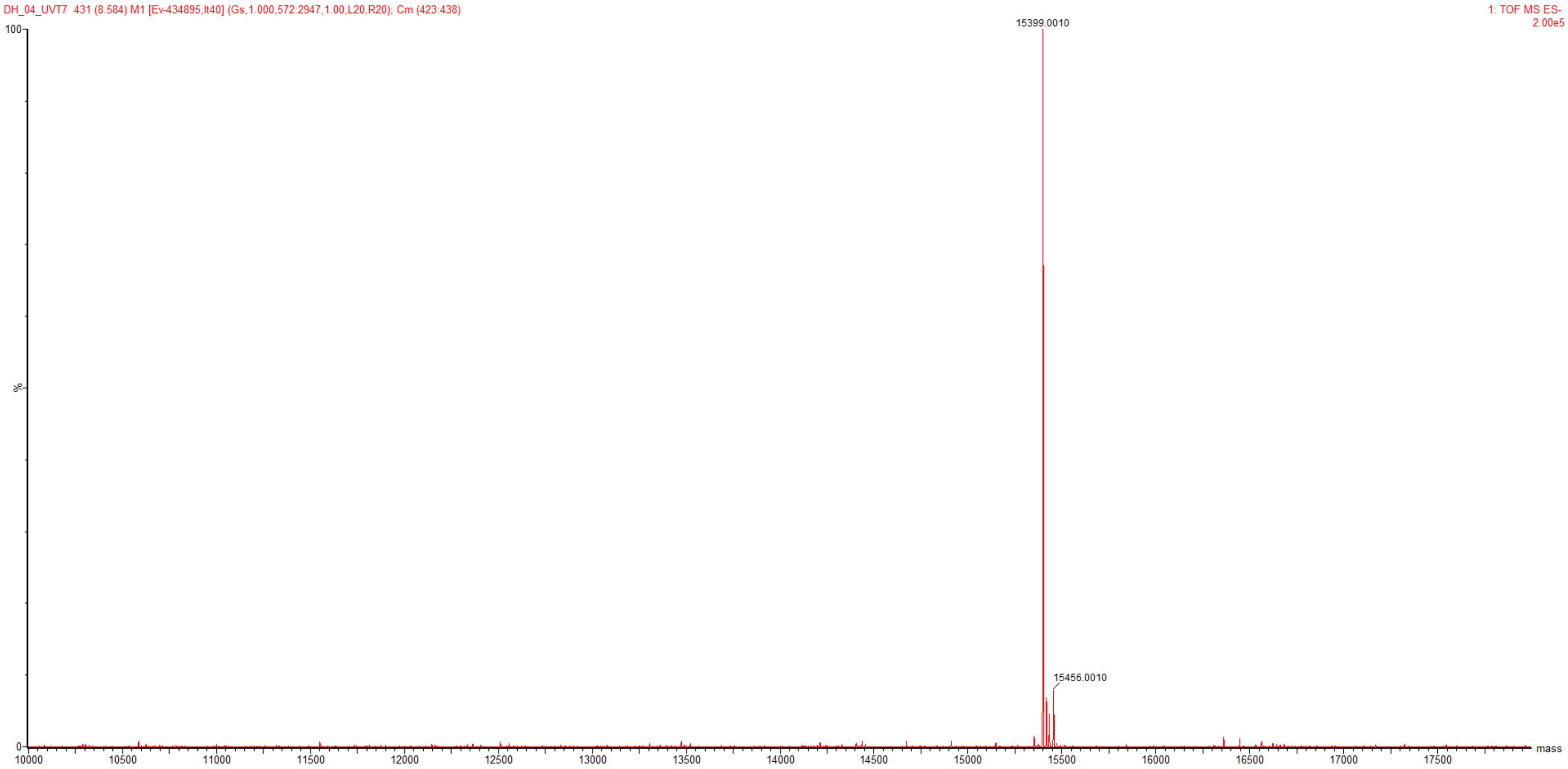

uvLA-T7 (AmberGen)

LC-UV and TIC

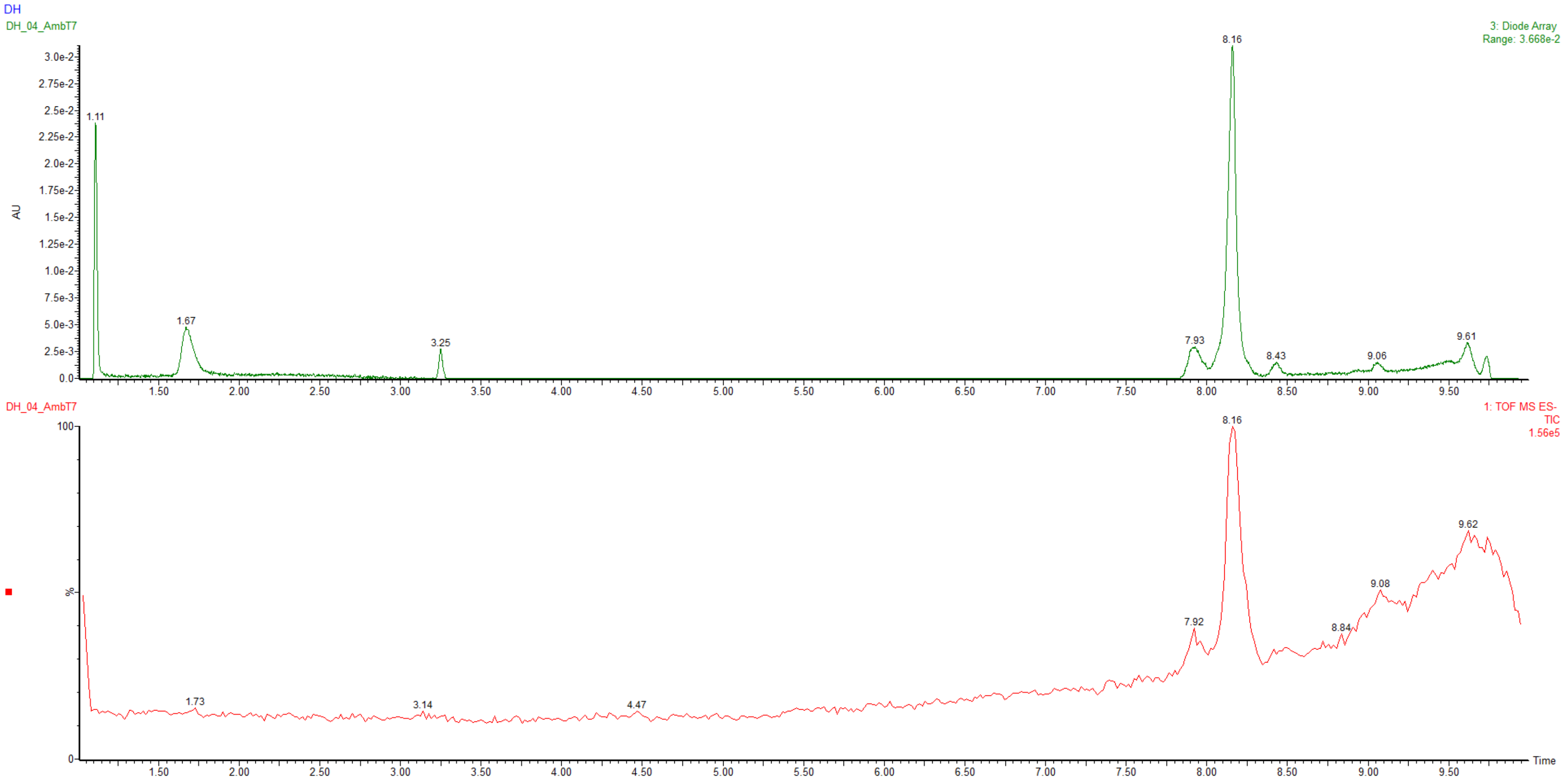

Mass After Deconvolution:

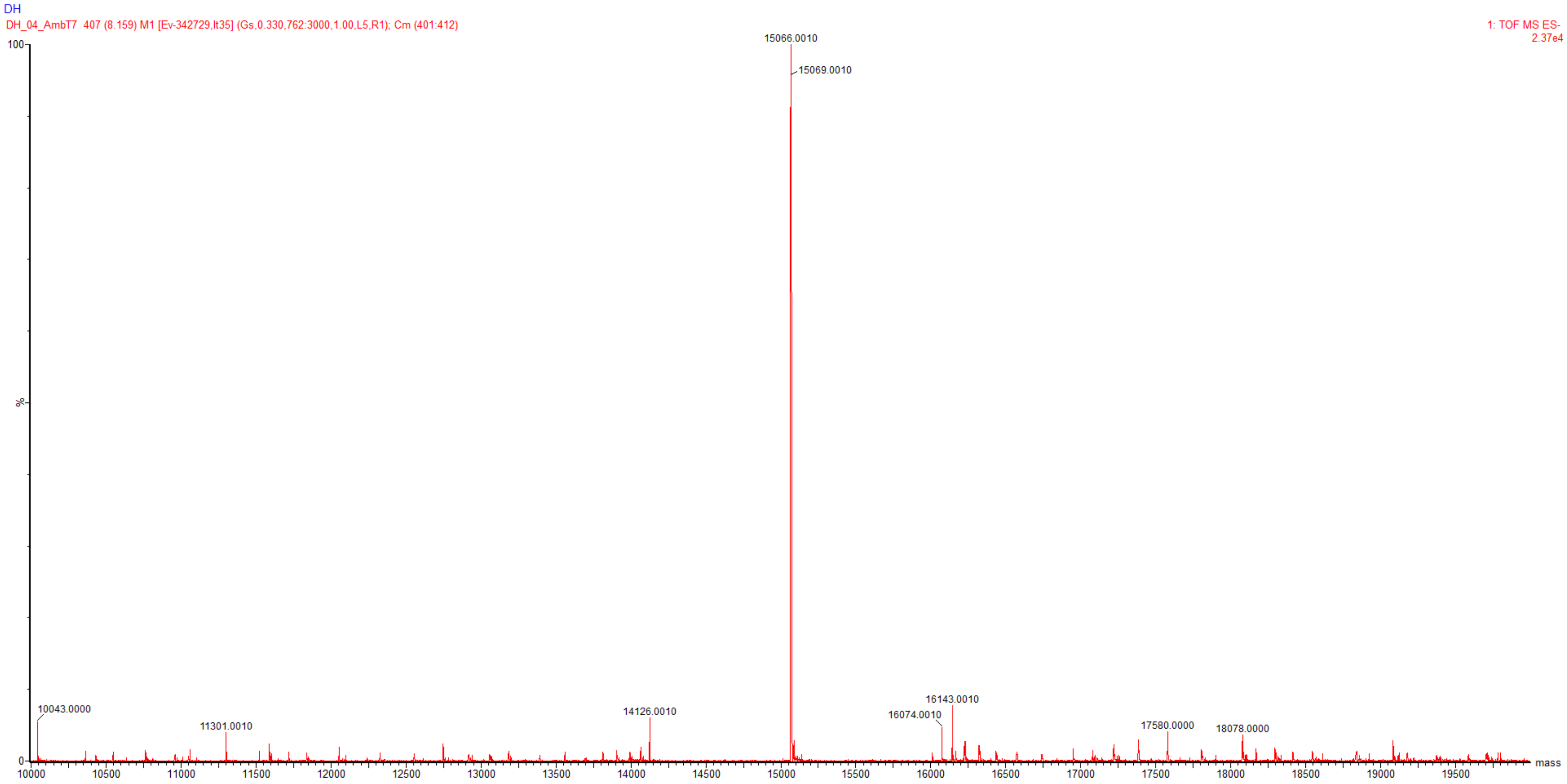

bLA-T7

LC-UV and TIC

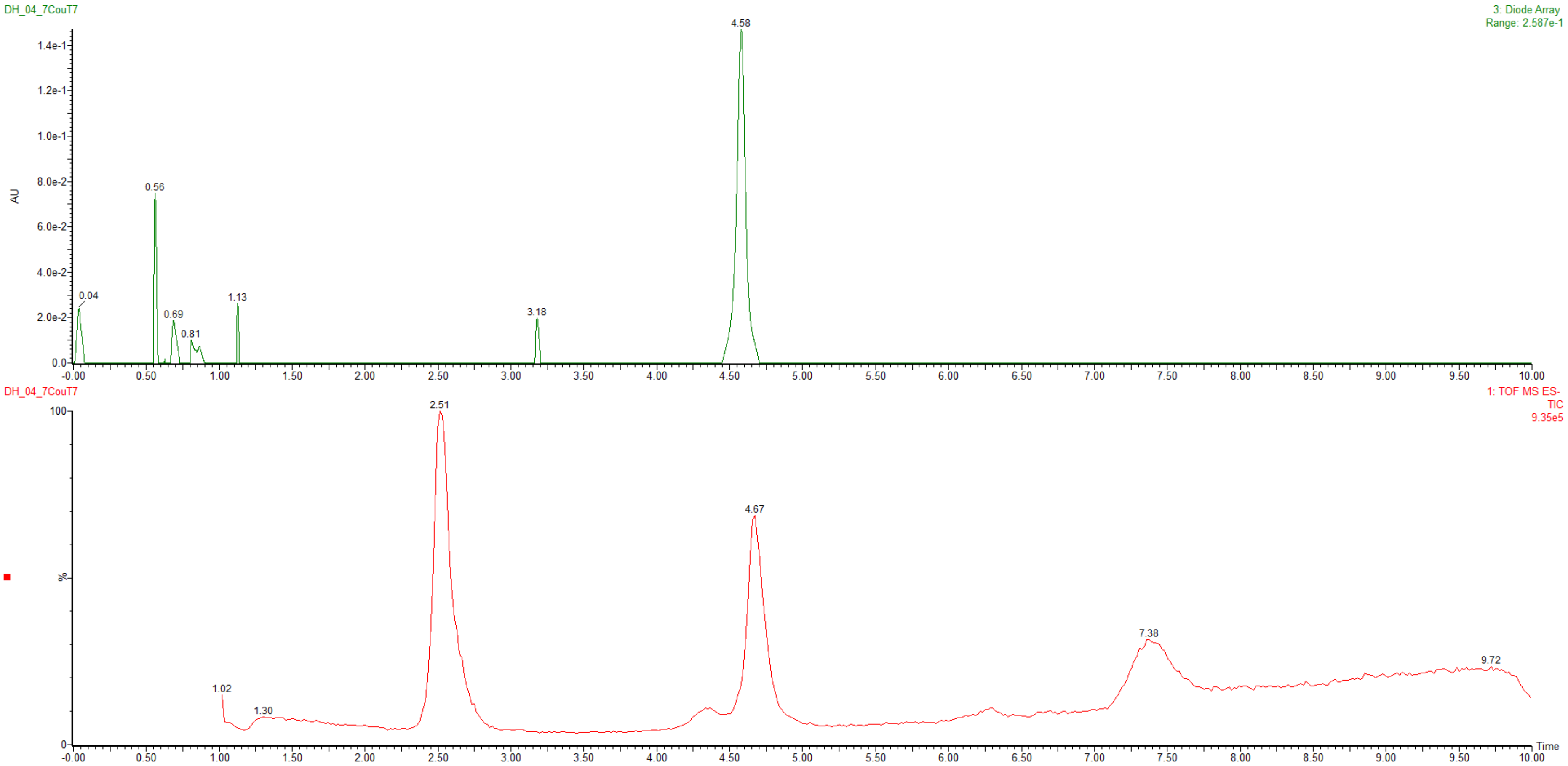

Mass After Deconvolution:

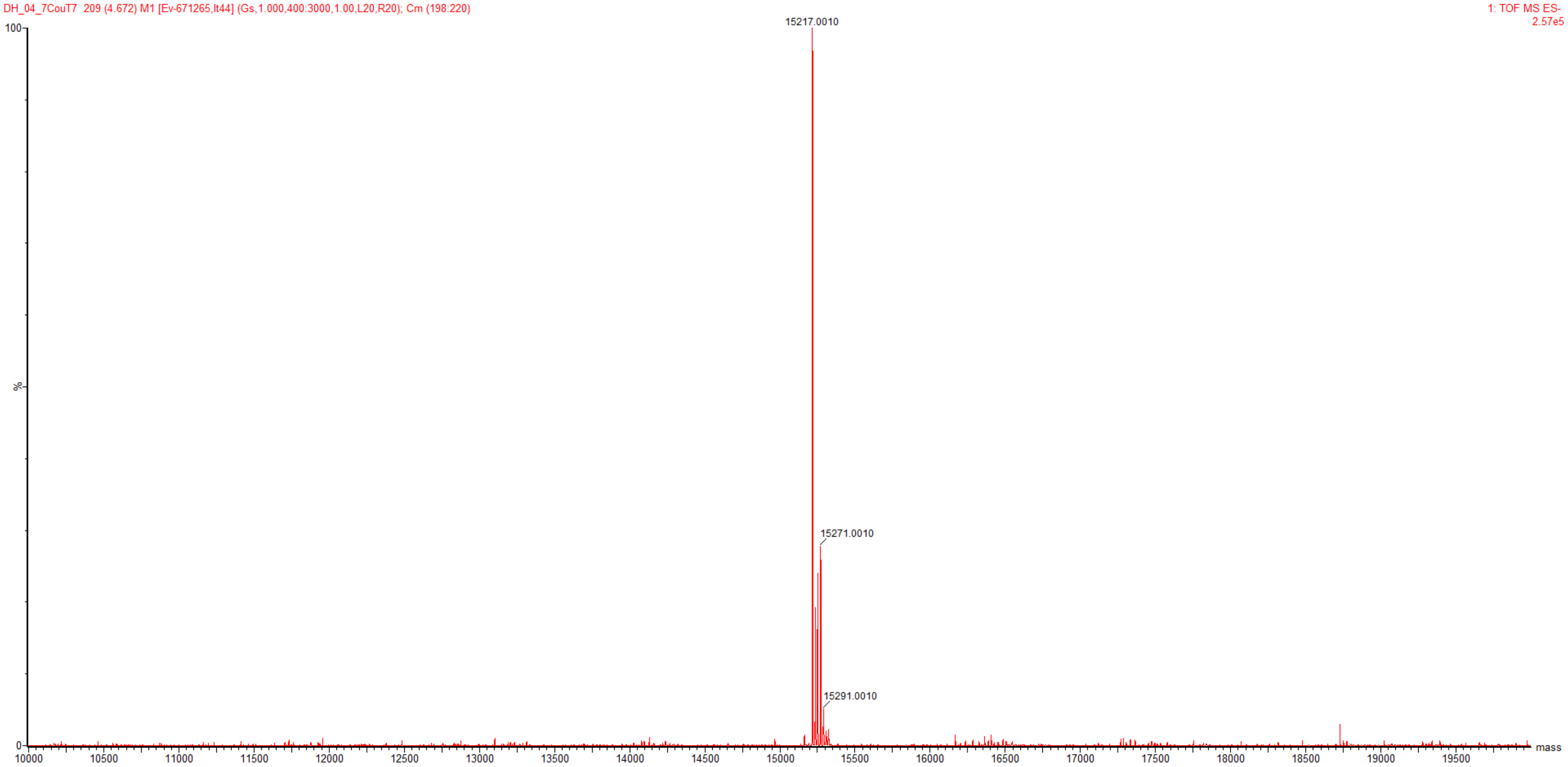

NMR Spectra

7-(diethylamino)-4-(hydroxymethyl)-2H-chromen-2-one 1

^{1}H-NMR (400 MHz, $CDCl_3$)

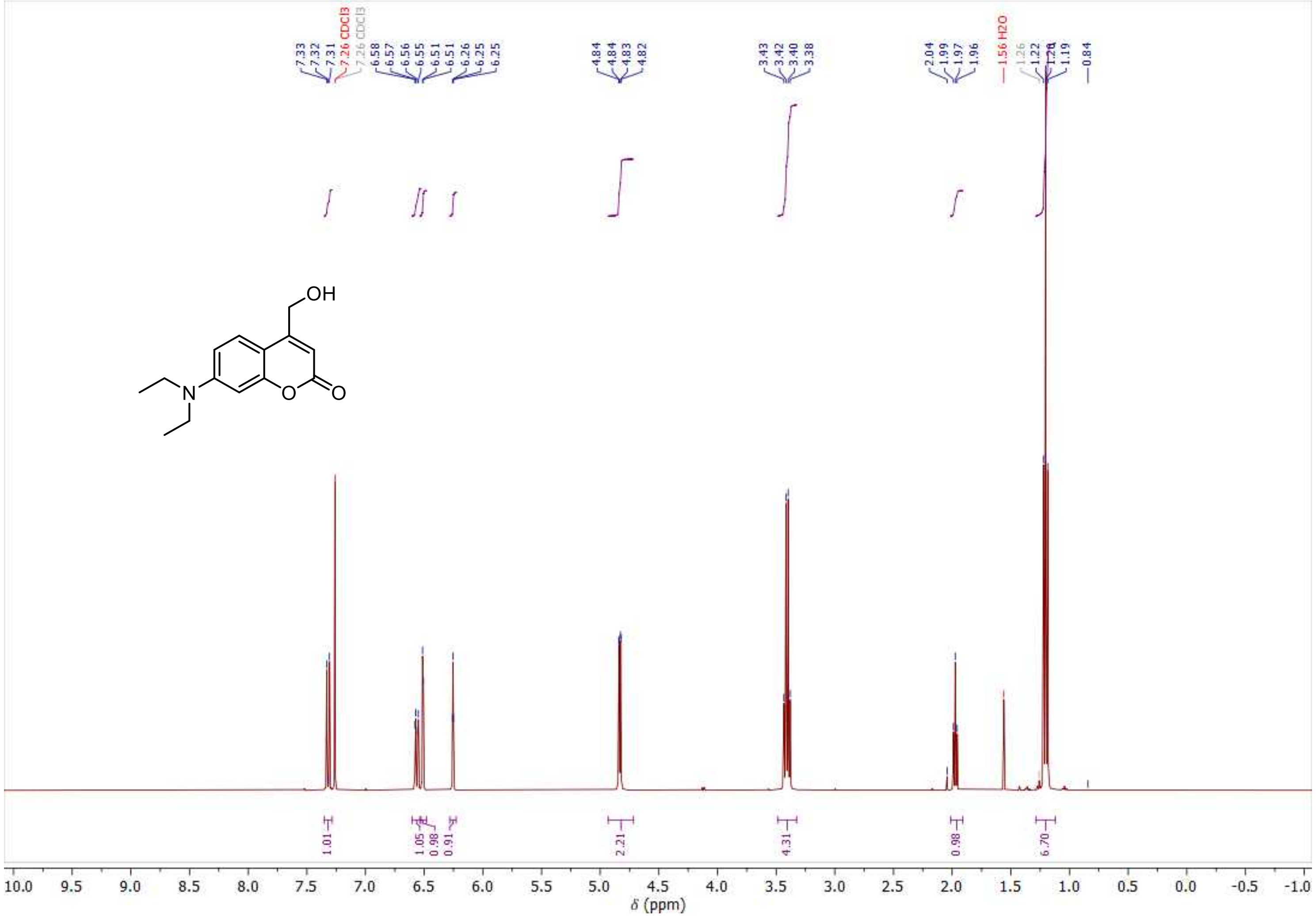

4-(((*tert*-butyldimethylsilyl)oxy)methyl)-7-(diethylamino)-2H-chromen-2-one 2

^{1}H-NMR (400 MHz, $CDCl_3$)

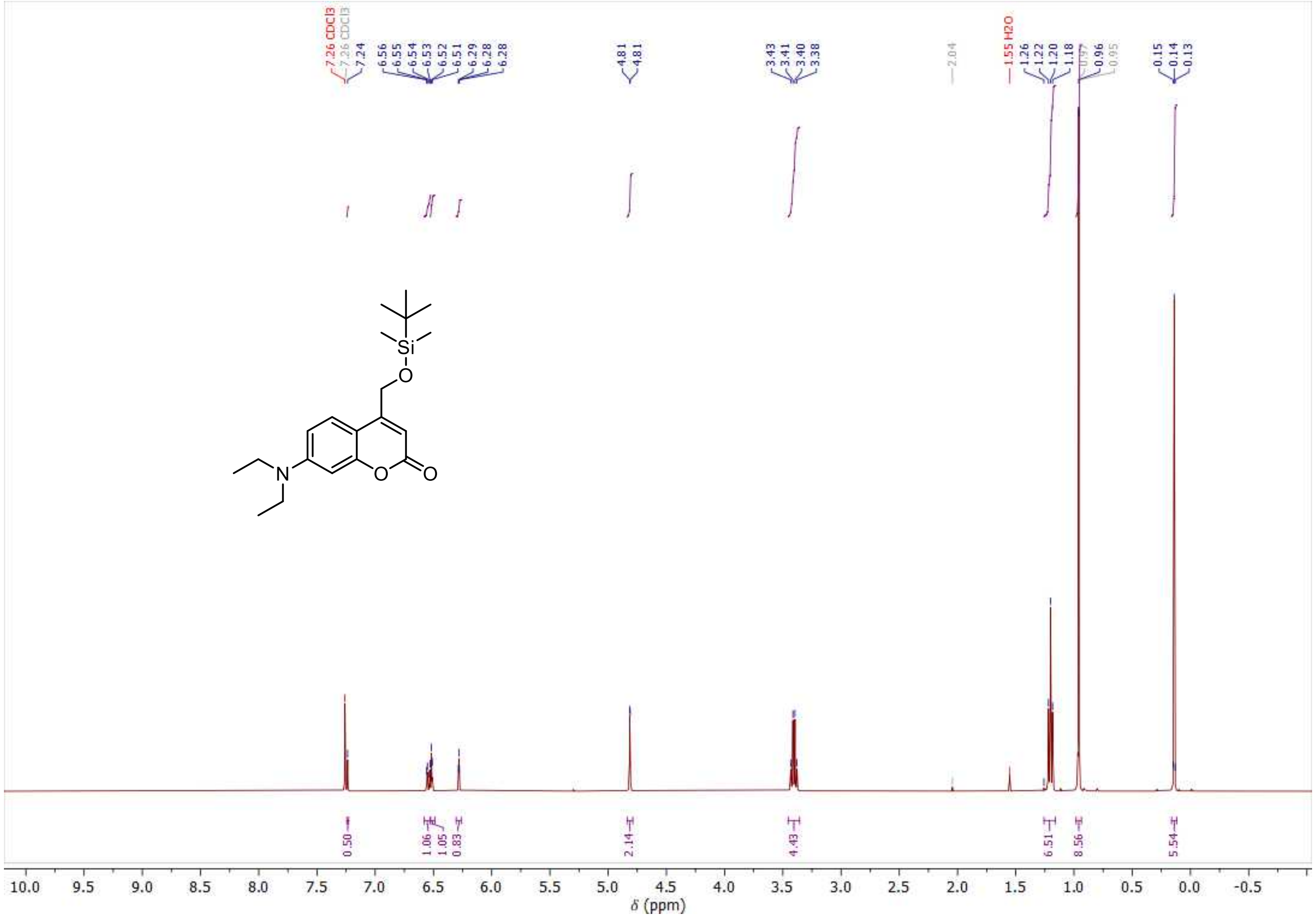

3-bromo-4-(((*tert*-butyldimethylsilyl)oxy)methyl)-7-(diethylamino)-2H-chromen-2-one 3

^{1}H-NMR (400 MHz, $CDCl_3$)

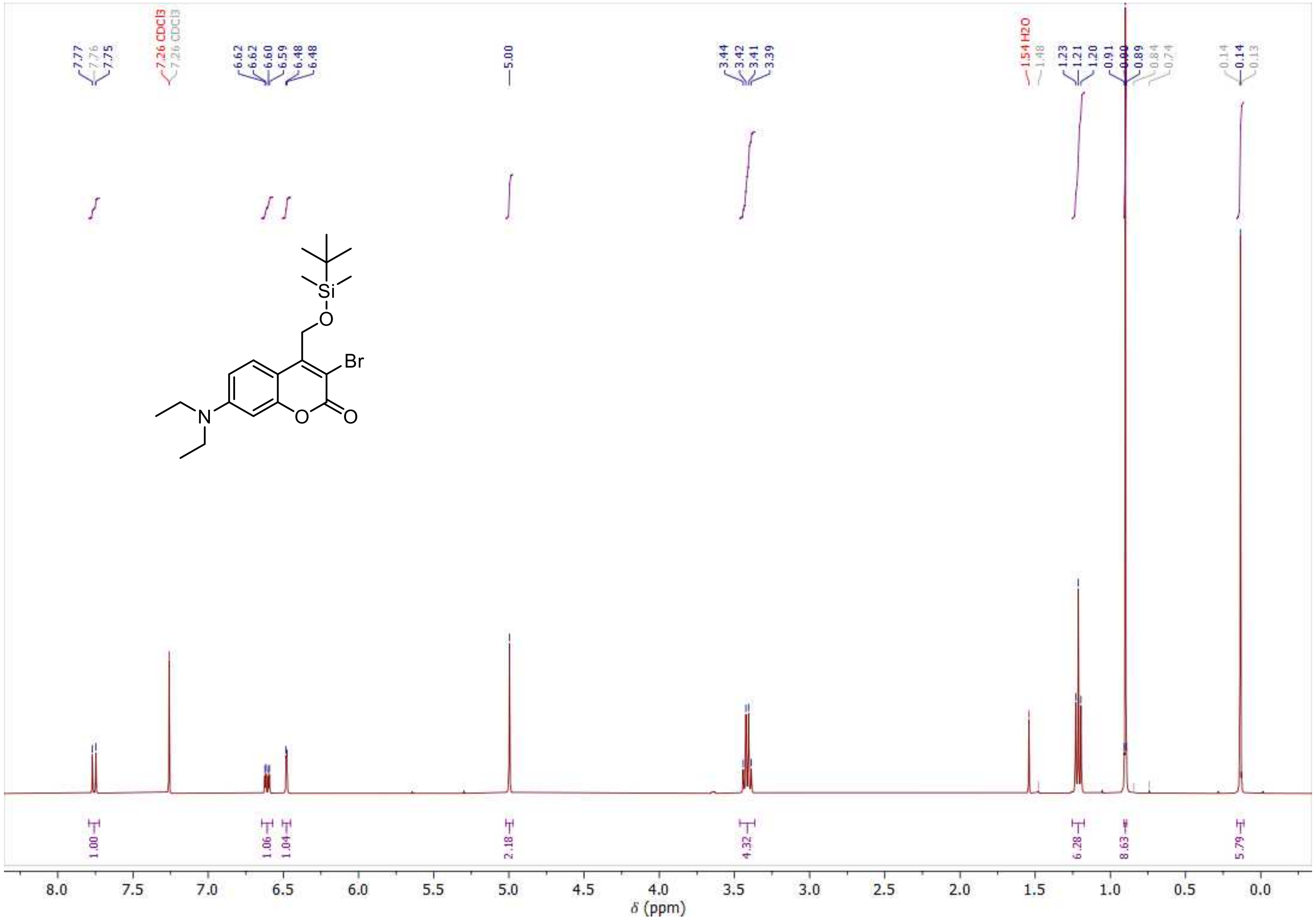

***tert*-butyl (*E*)-3-(4-(((*tert*-butyldimethylsilyl)oxy)methyl)-7-(diethylamino)-2-oxo-2H-chromen-3-yl)acrylate 4**

^{1}H-NMR (400 MHz, $CDCl_3$)

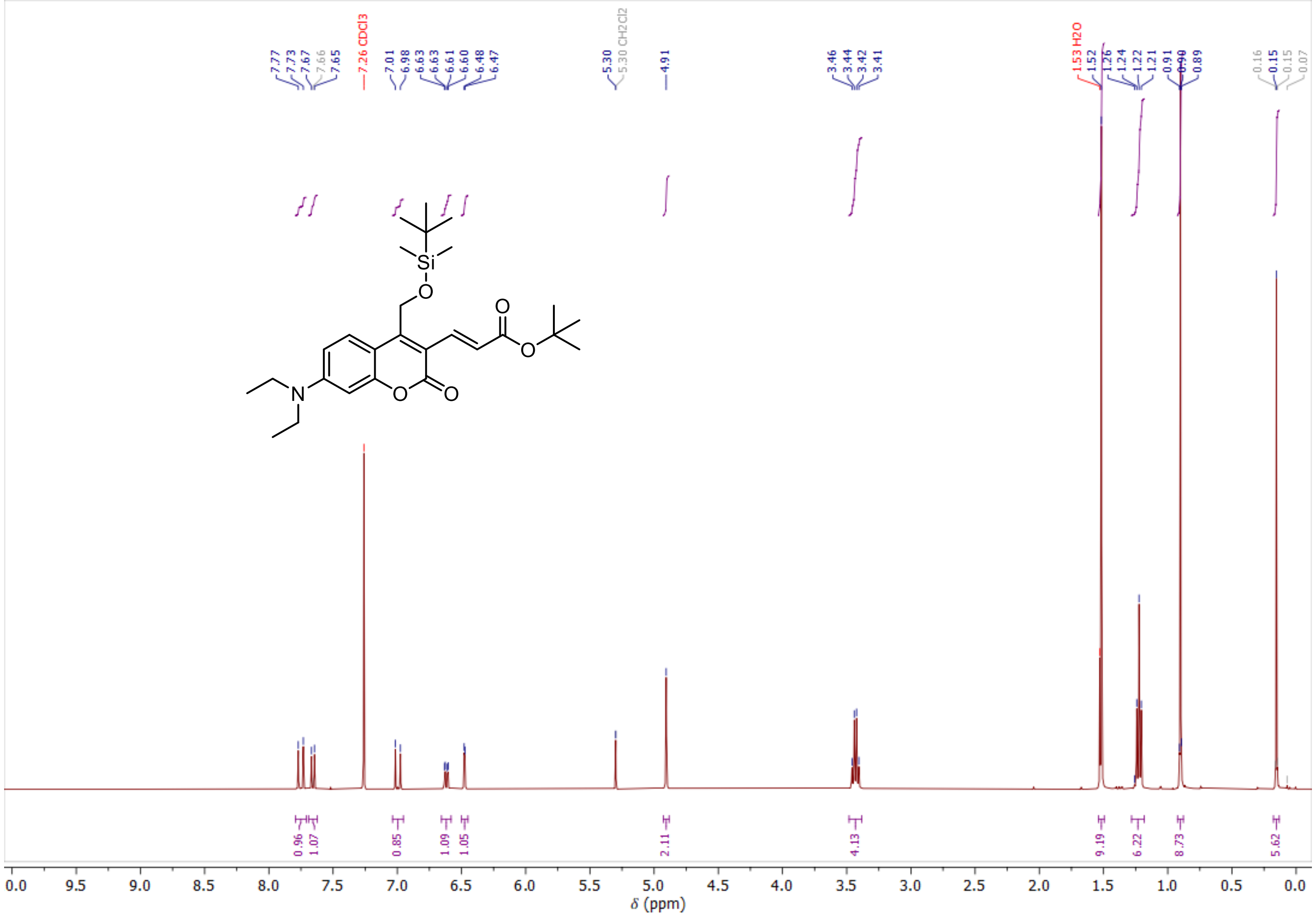

***tert*-butyl-(*E*)-(2-(2-(2-(3-(4-(((tert-butyldimethylsilyl)oxy)methyl)-7-(diethylamino)-2-oxo-2H-chromen-3-yl)acrylamido)ethoxy)ethoxy)ethyl)carbamate 5a**

^{1}H-NMR (600 MHz, $CDCl_3$)

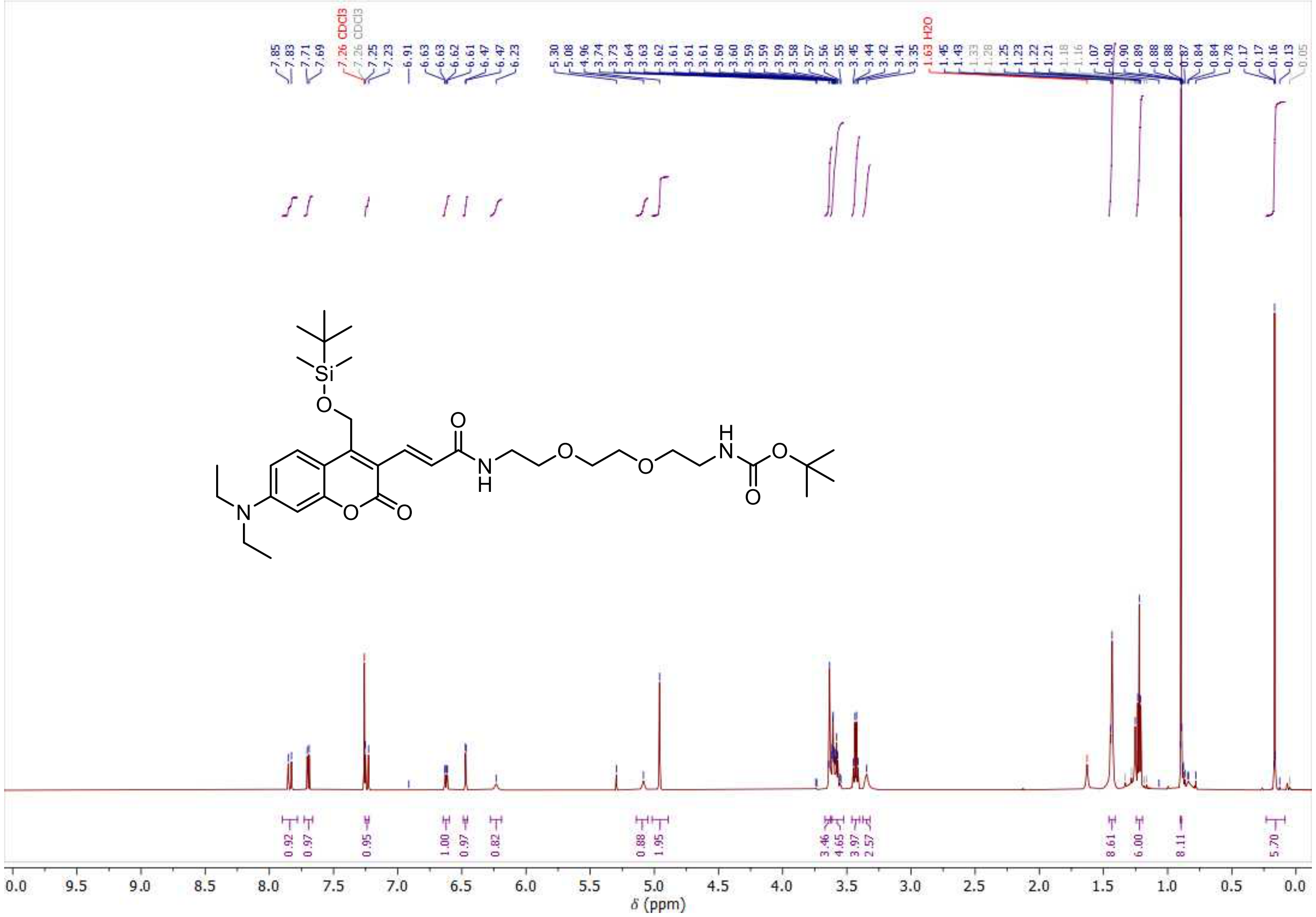

^{13}C-NMR (126 MHz, $CDCl_3$)

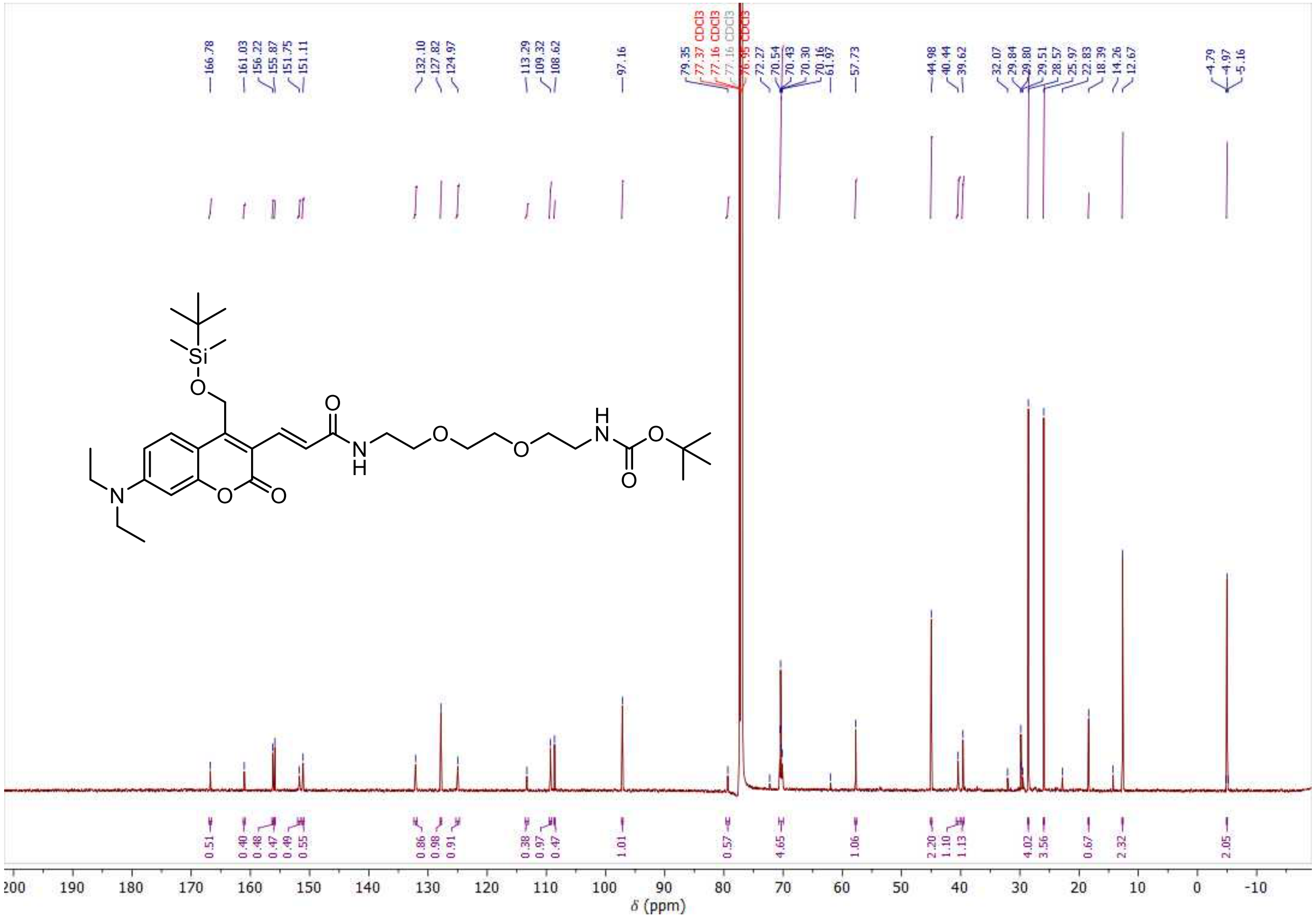

***tert*-butyl (E)-(2-(2-(2-(3-(7-(diethylamino)-4-(hydroxymethyl)-2-oxo-2H-chromen-3-yl)acrylamido)ethoxy)ethoxy)ethyl)carbamate 5b**

^{1}H-NMR (500 MHz, $CDCl_3$)

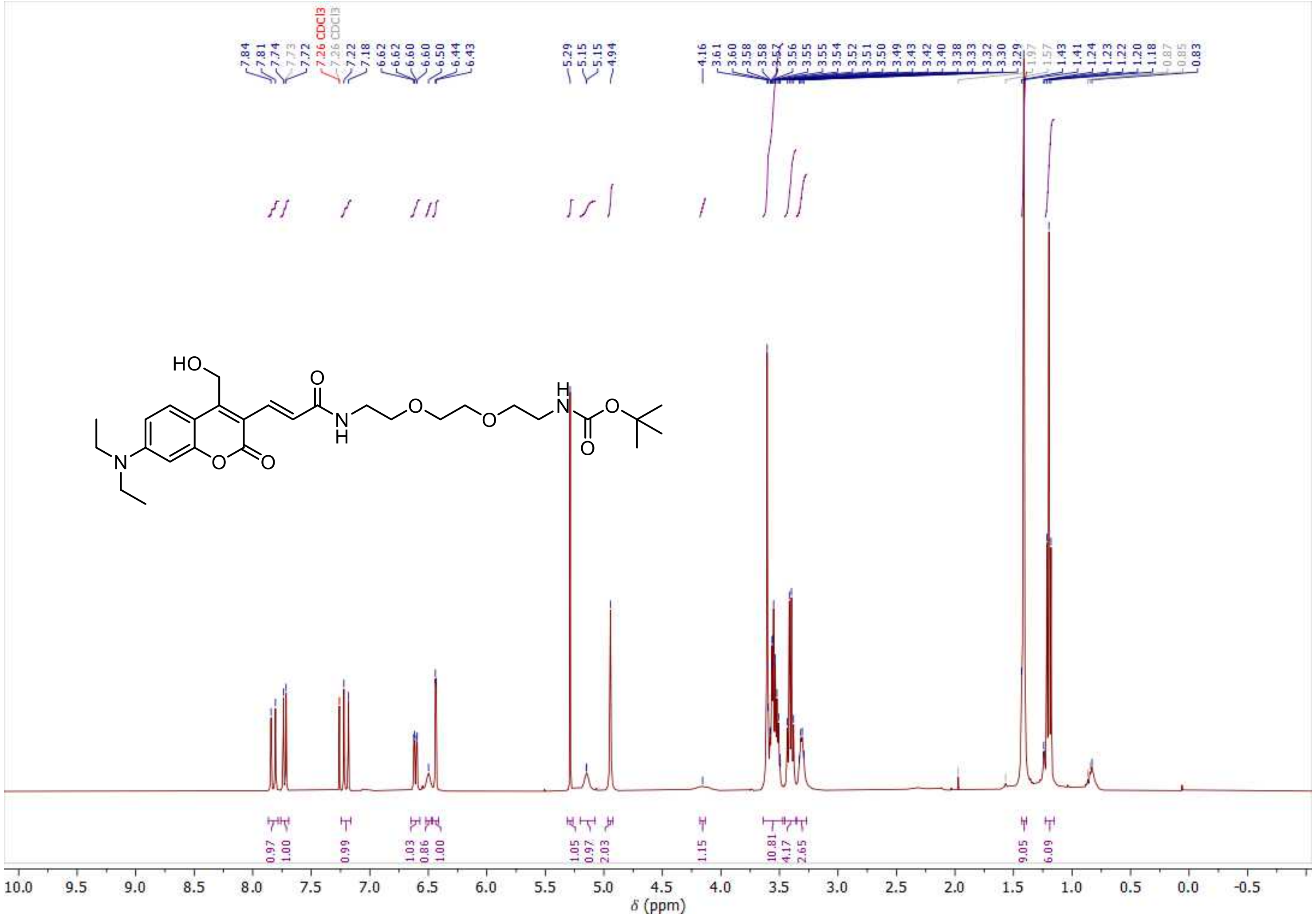

^{13}C-NMR (126 MHz, $CDCl_3$)

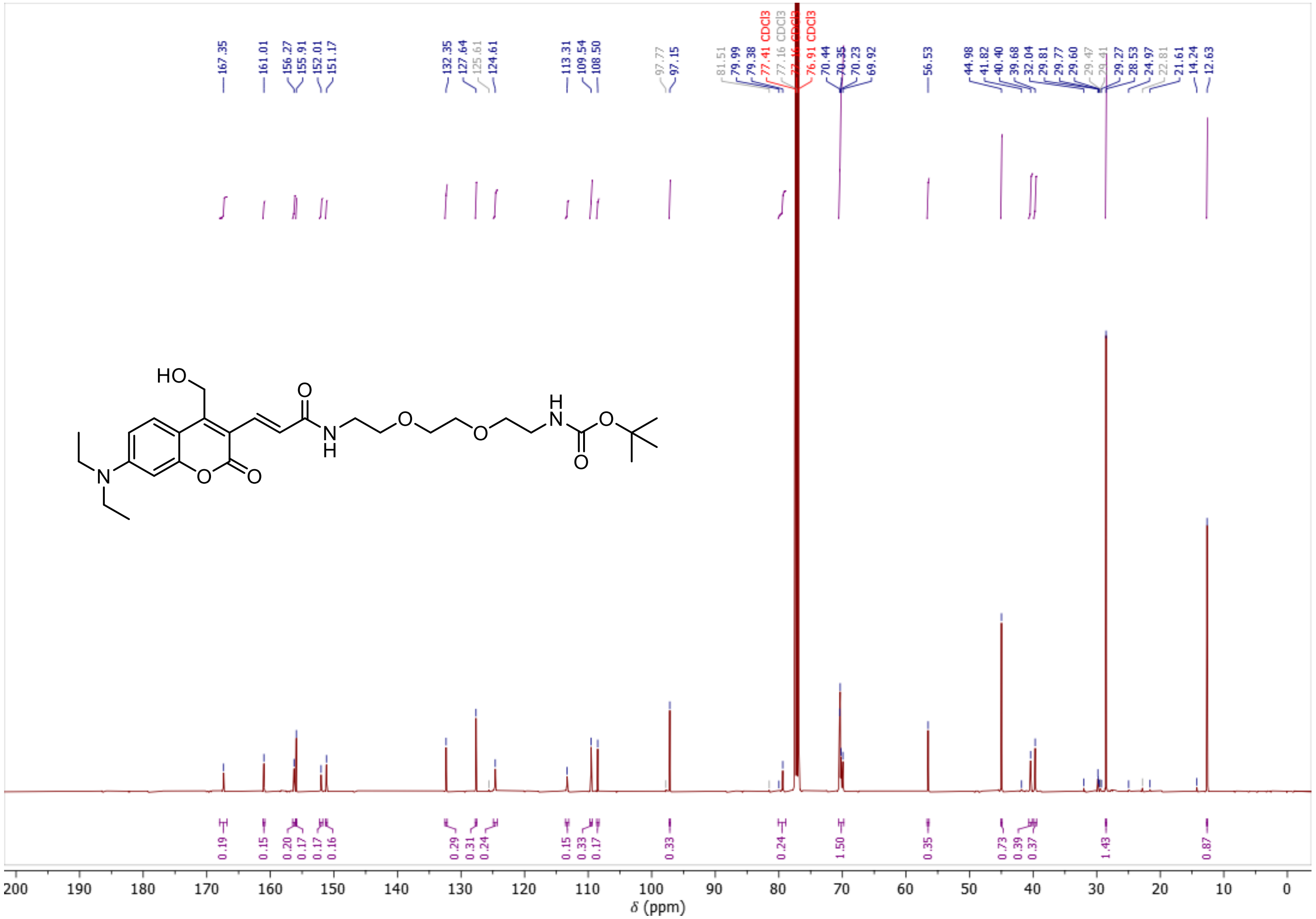

N-(2-(2-(2-((E)-3-(4-(((tert-butyldimethylsilyl)oxy)methyl)-7-(diethylamino)-2-oxo-2H-chromen-3-yl)acrylamido)ethoxy)ethoxy)ethyl)-5-((3aS,4S,6aR)-2-oxohexahydro-1H-thieno[3,4-d]imidazol-4-yl)pentanamide 6

^{1}H-NMR (500 MHz, $CDCl_3$)

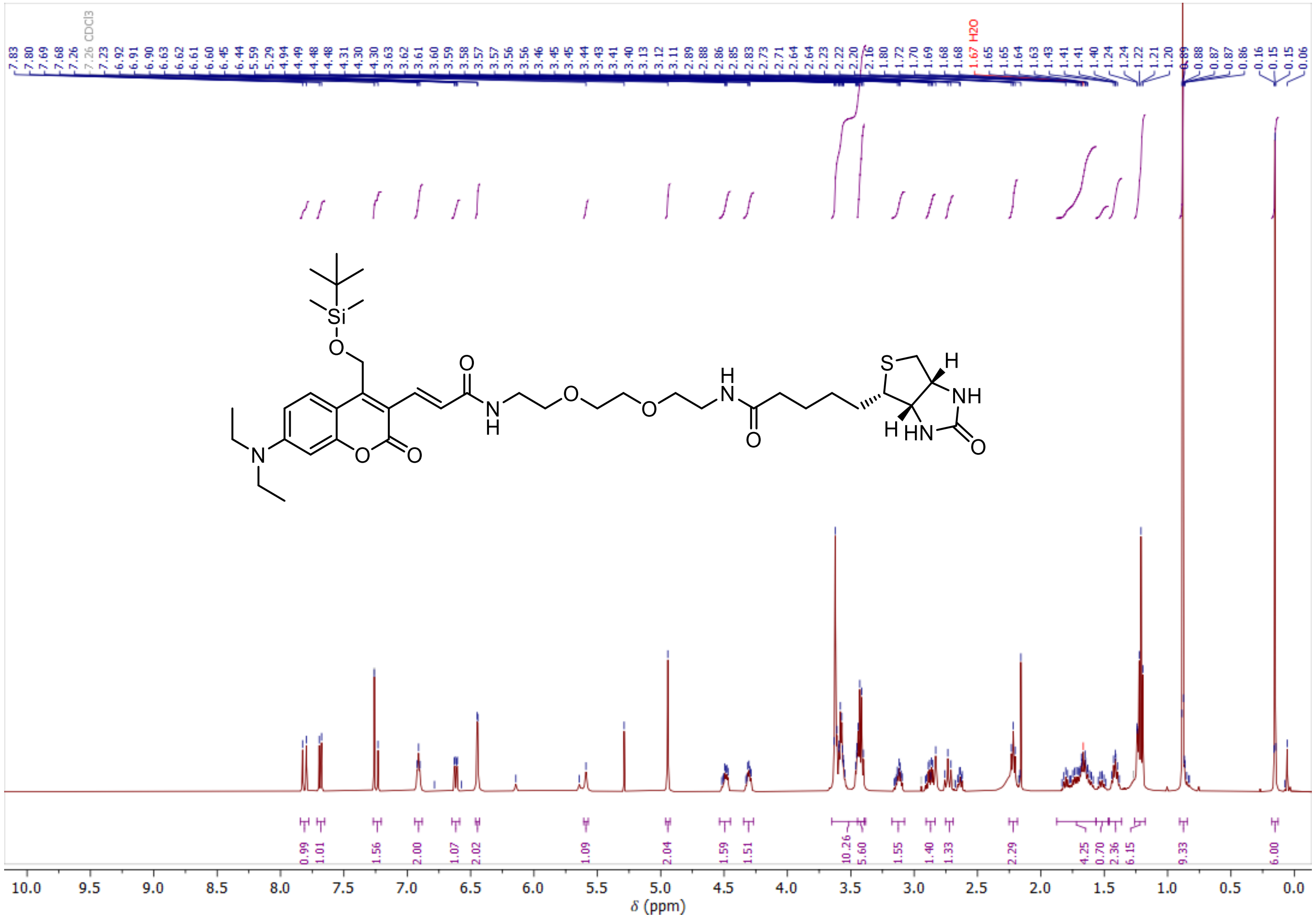

^{13}C-NMR (126 MHz, $CDCl_3$)

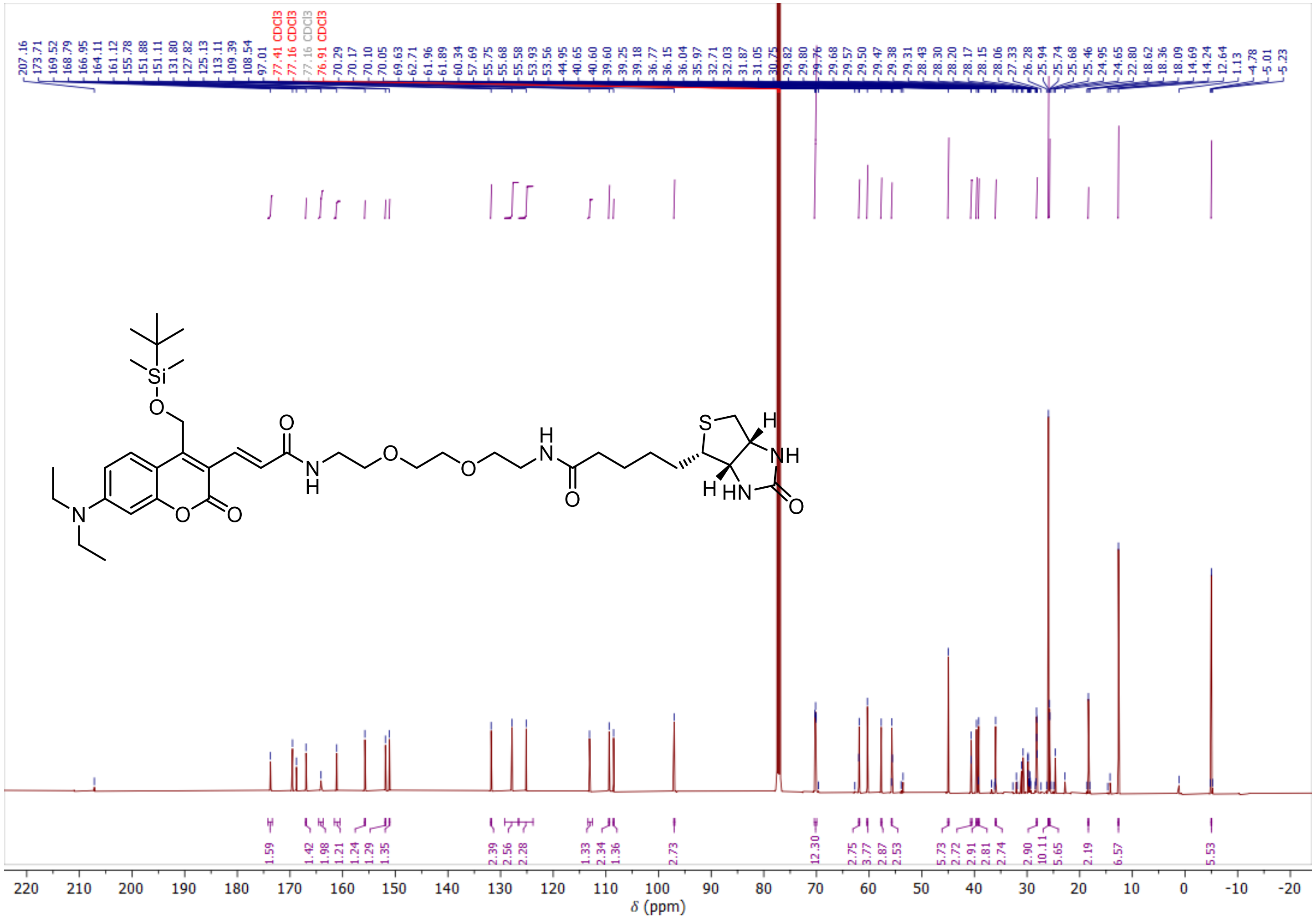

N-(2-(2-(2-((E)-3-(7-(diethylamino)-4-(hydroxymethyl)-2-oxo-2H-chromen-3-yl)acrylamido)ethoxy)ethoxy)ethyl)-5-((3aS,4S,6aR)-2-oxohexahydro-1H-thieno[3,4-d]imidazol-4-yl)pentanamide 7

^{1}H-NMR (500 MHz, DMSO)

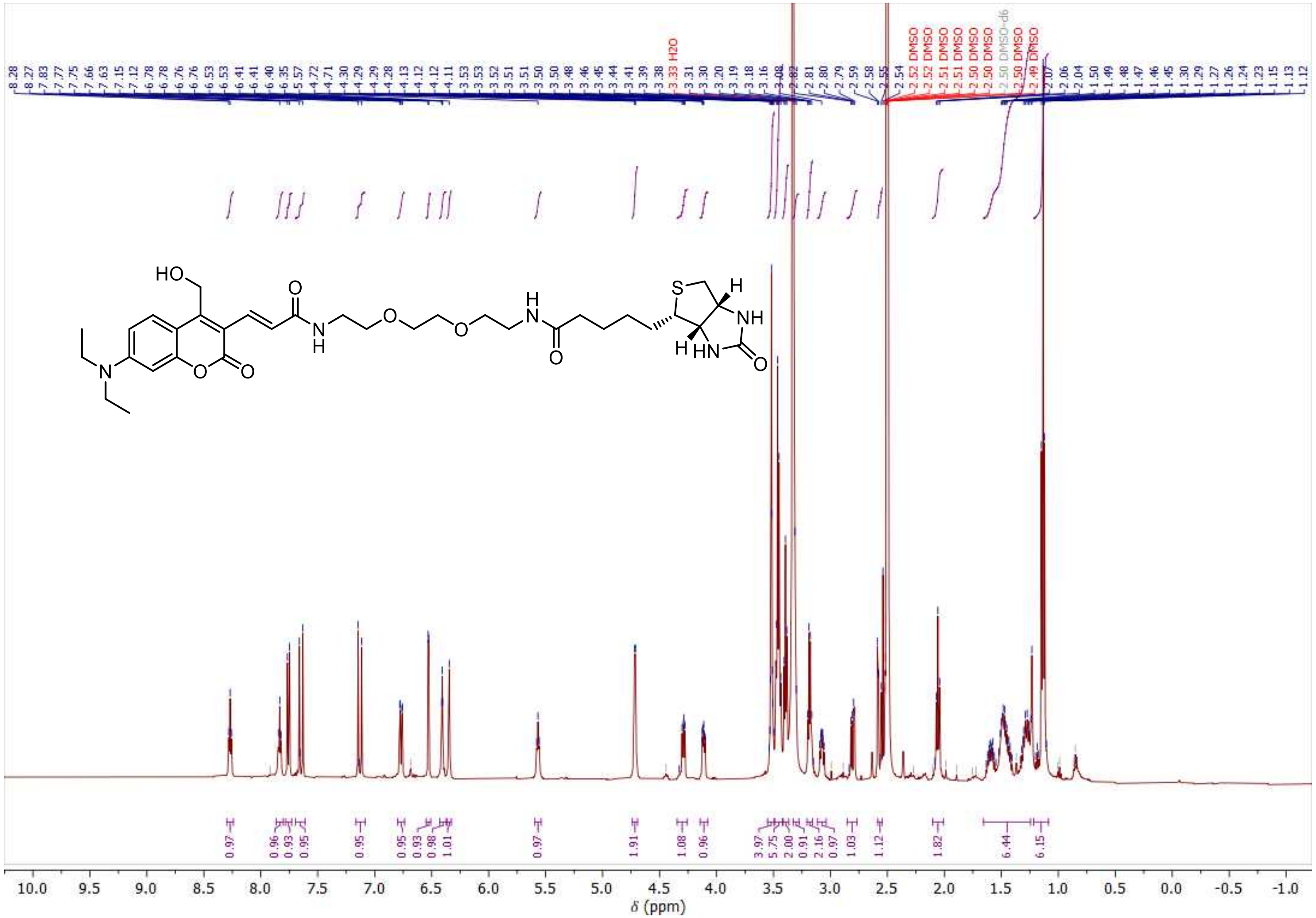

^{13}C-NMR (126 MHz, DMSO)

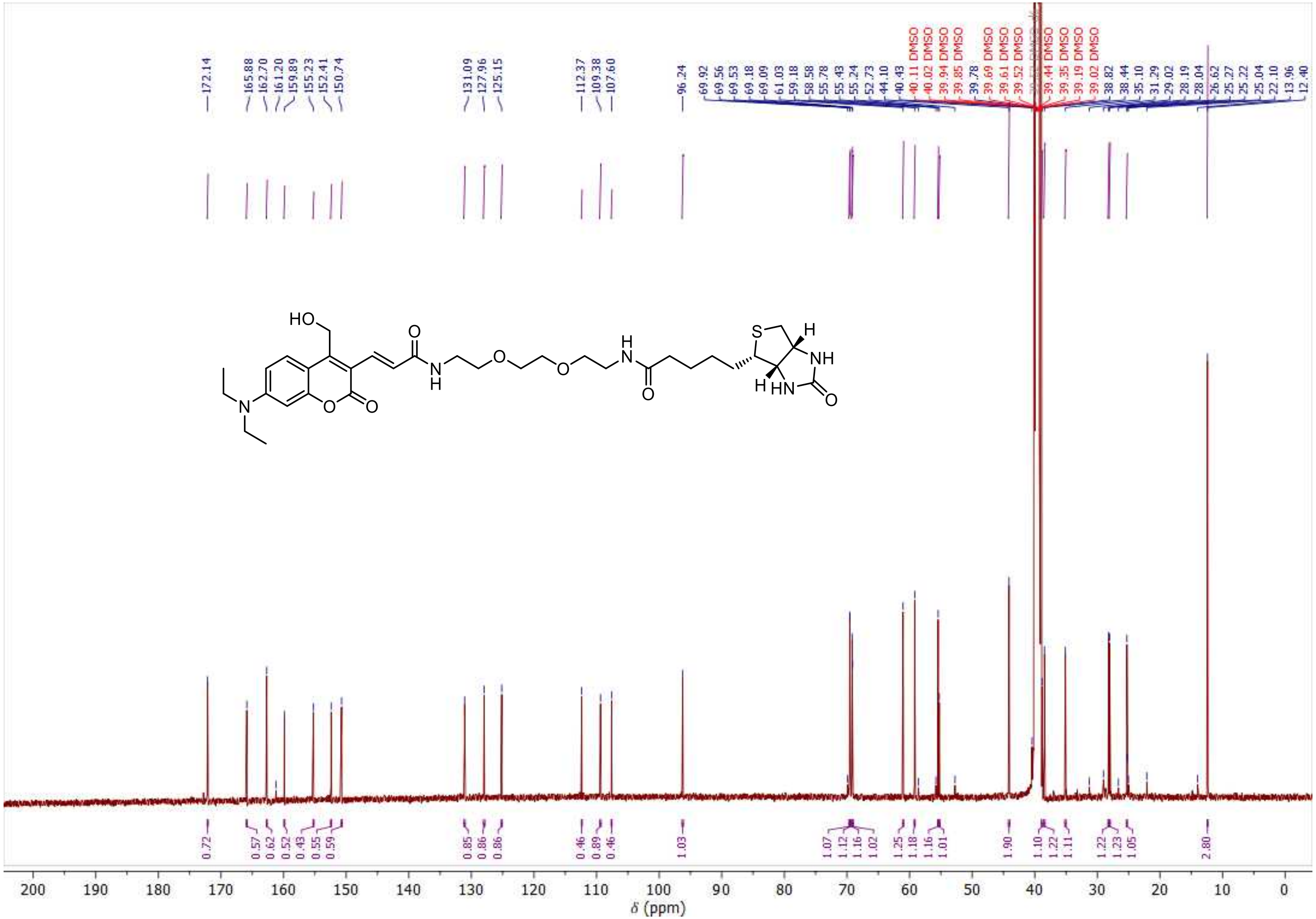

(7-(diethylamino)-3-((E)-3,14-dioxo-18-((3aS,4S,6aR)-2-oxohexahydro-1H-thieno[3,4-d]imidazol-4-yl)-7,10-dioxa-4,13-diazaoctadec-1-en-1-yl)-2-oxo-2H-chromen-4-yl)methyl (2,5-dioxopyrrolidin-1-yl) carbonate 8a

^{1}H-NMR (500 MHz, $CDCl_3$)

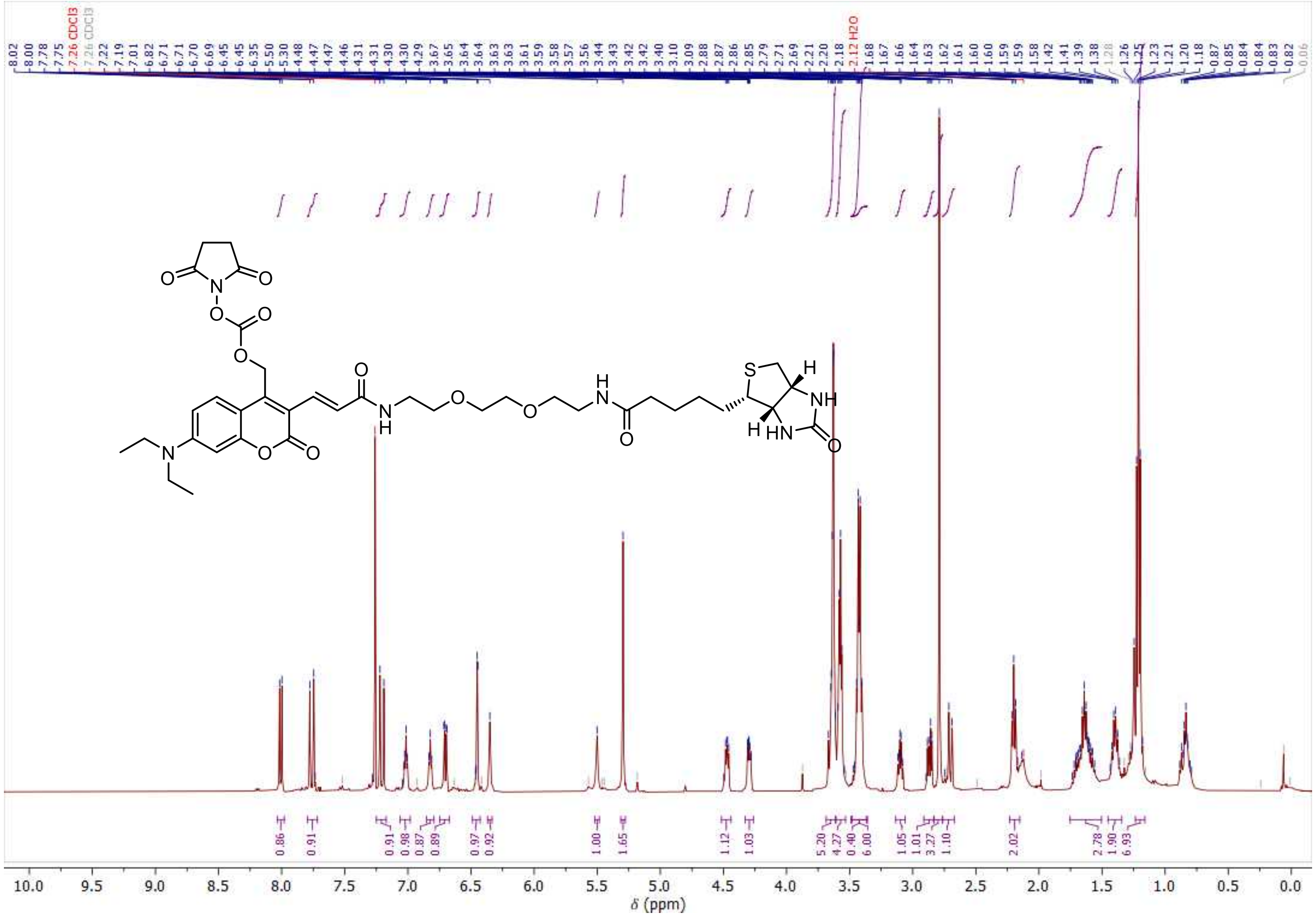

^{13}C-NMR (126 MHz, $CDCl_3$)

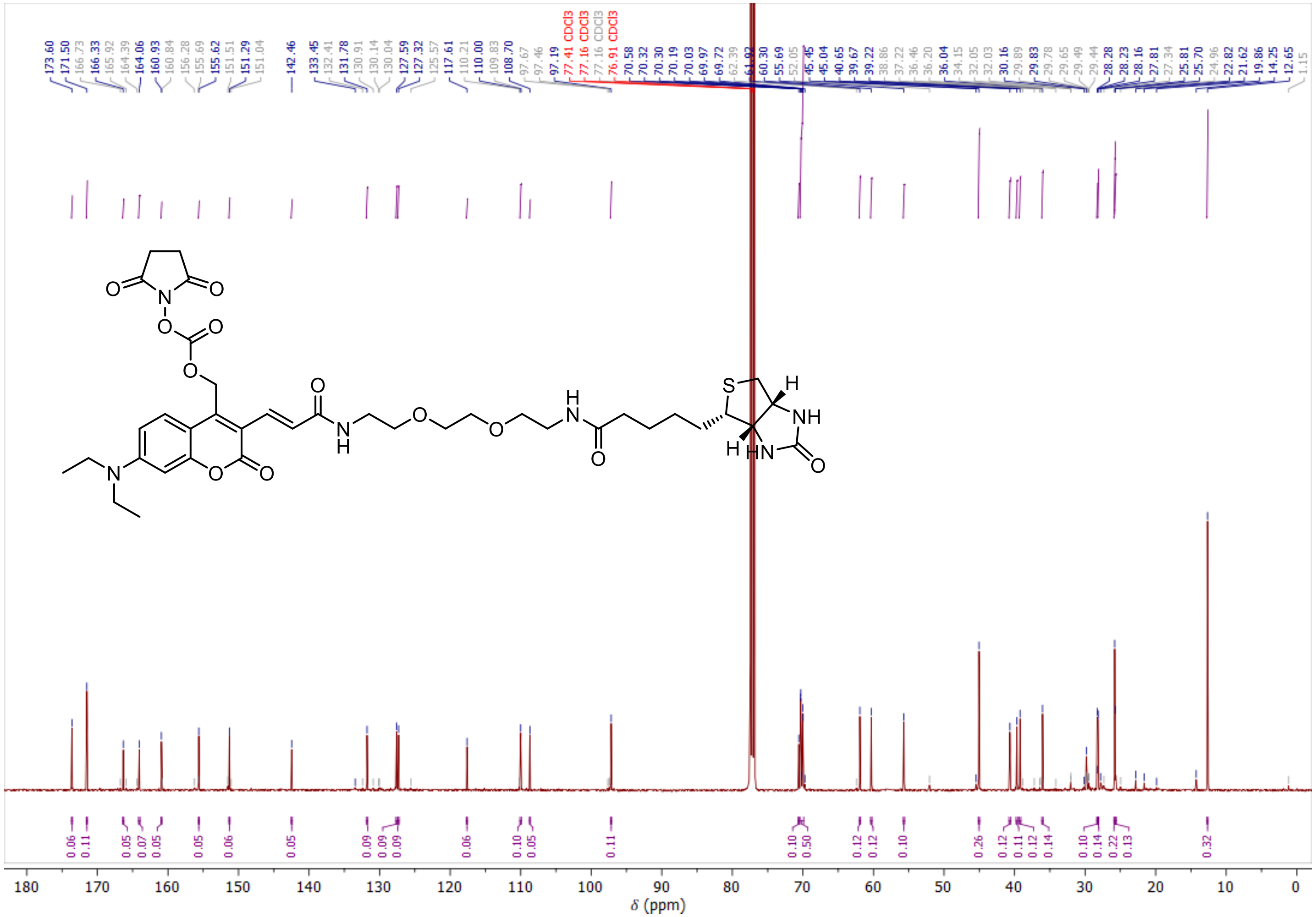

(7-(diethylamino)-3-((E)-3,14-dioxo-18-((3aS,4S,6aR)-2-oxohexahydro-1H-thieno[3,4-d]imidazol-4-yl)-7,10-dioxa-4,13-diazaoctadec-1-en-1-yl)-2-oxo-2H-chromen-4-yl)methyl (perfluorophenyl) carbonate 8b

^{1}H-NMR (500 MHz, $CDCl_3$)

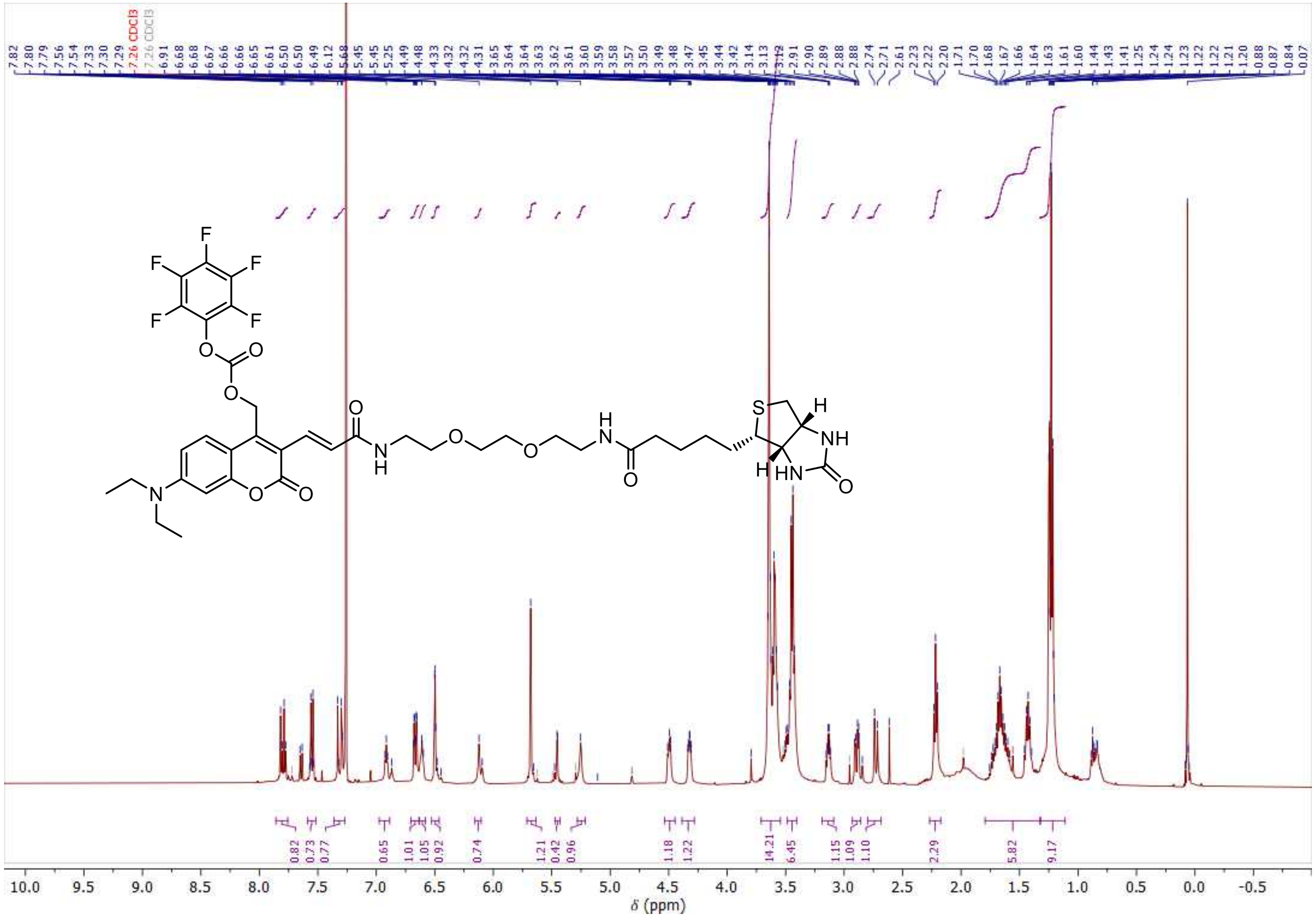

^{13}C-NMR (126 MHz, $CDCl_3$)

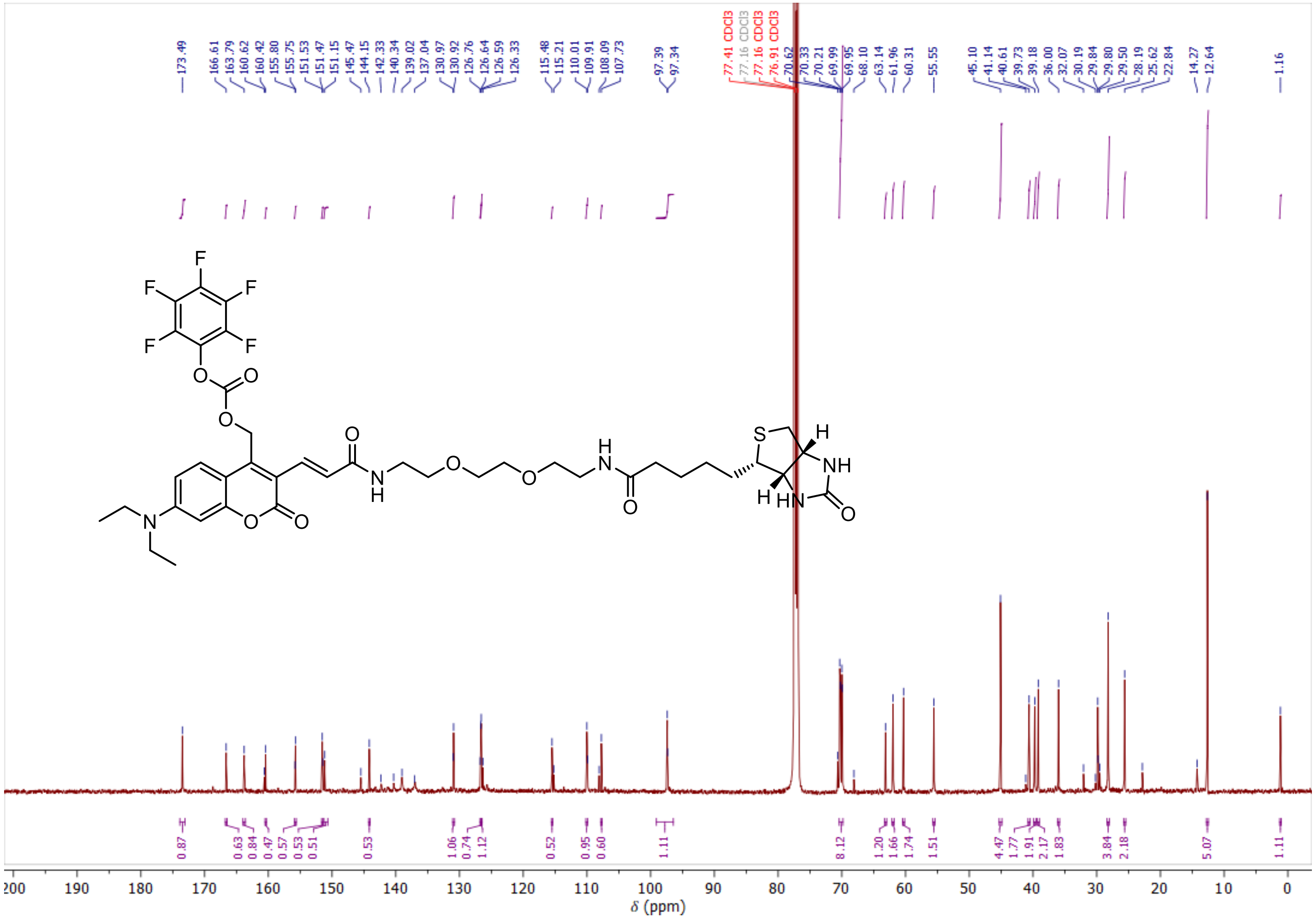

^{19}F-NMR (471 MHz, $CDCl_3$)

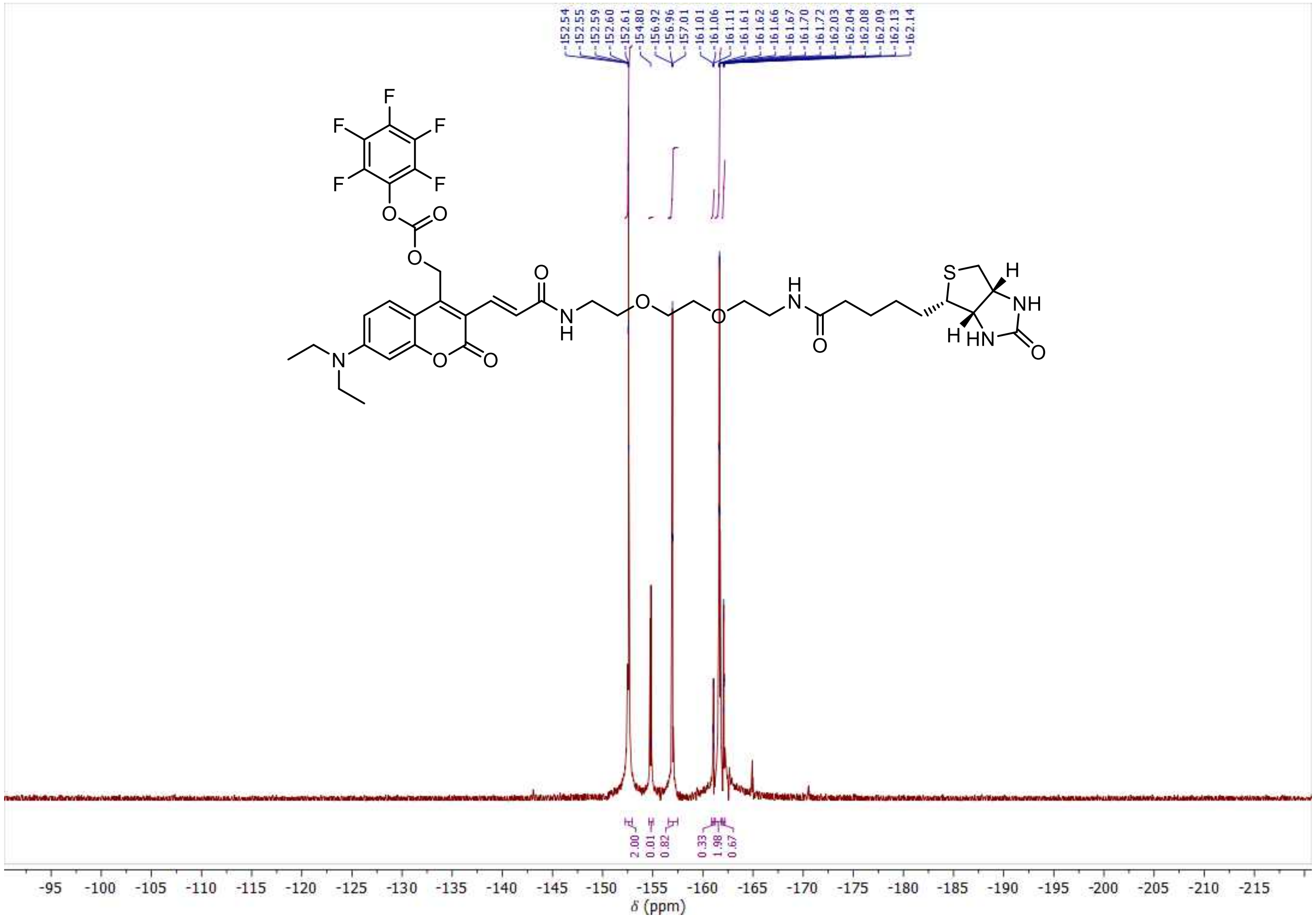

(7-(diethylamino)-3-((E)-3,14-dioxo-18-((3aS,4S,6aR)-2-oxohexahydro-1H-thieno[3,4-d]imidazol-4-yl)-7,10-dioxa-4,13-diazaoctadec-1-en-1-yl)-2-oxo-2H-chromen-4-yl)methyl (4-nitrophenyl) carbonate 8c

^{1}H-NMR (600 MHz, $CDCl_3$)

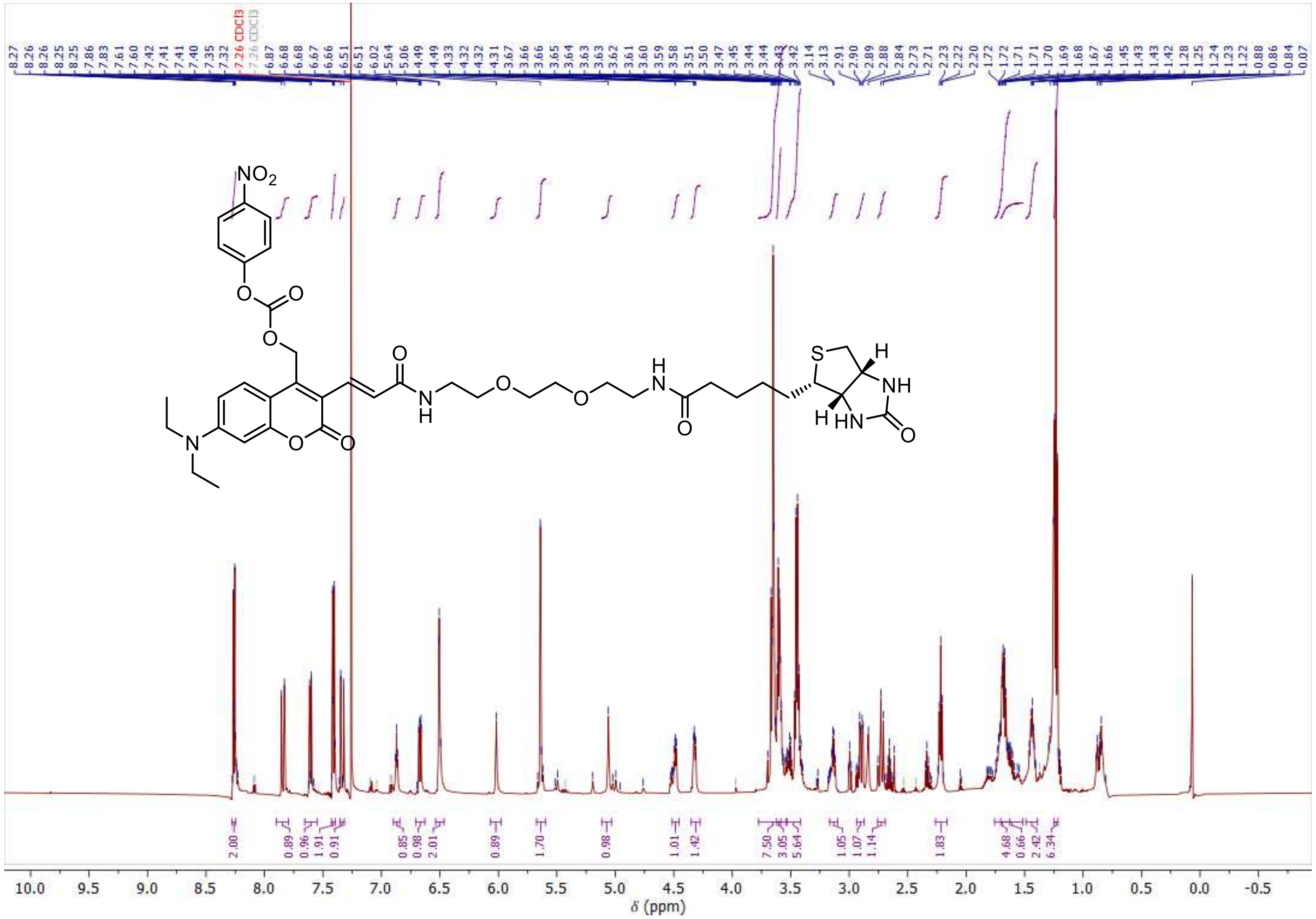

^{13}C-NMR (151 MHz, $CDCl_3$)

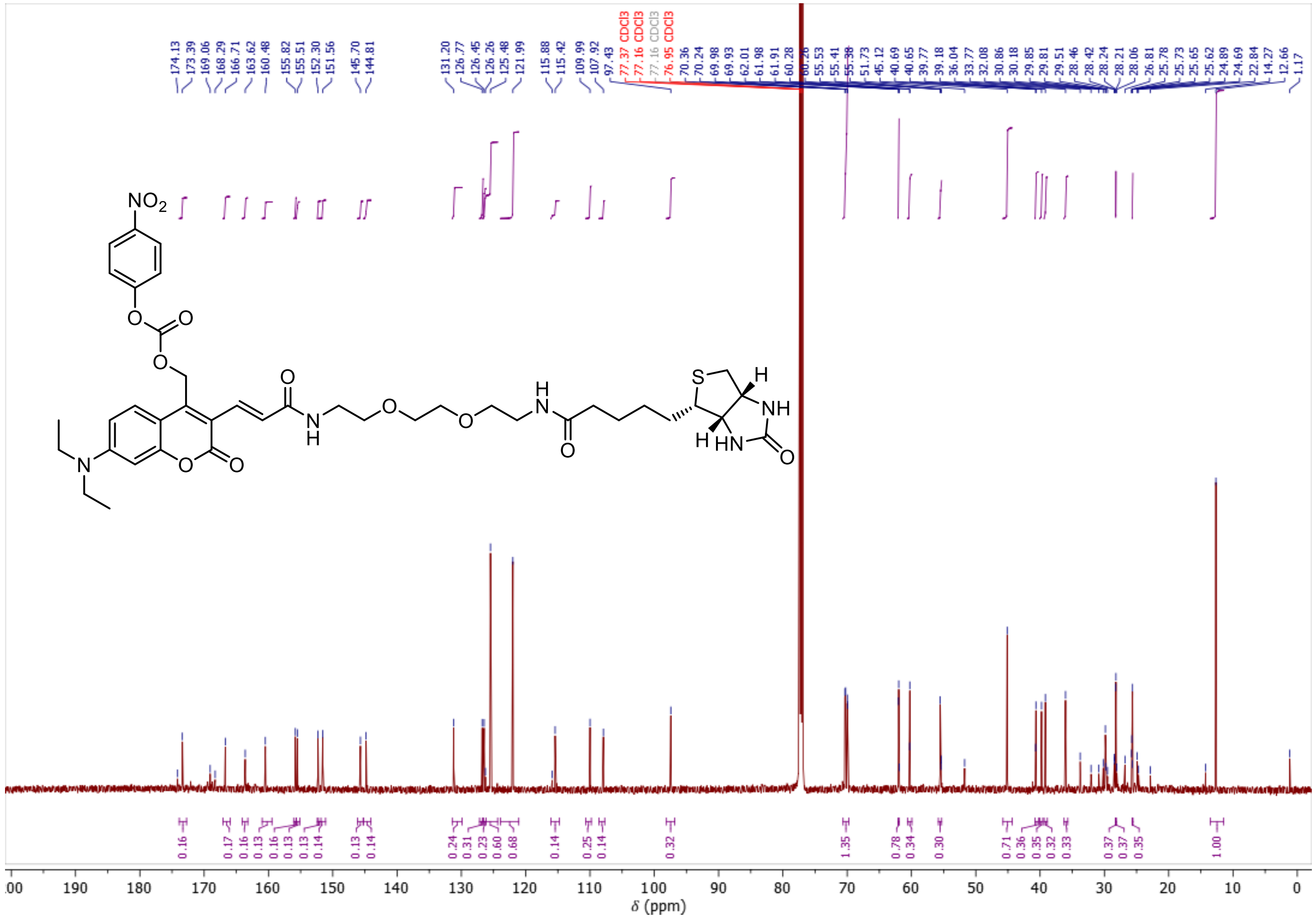

***tert*-butyl (2-(2-(2-aminoethoxy)ethoxy)ethyl)carbamate S1**

^{1}H-NMR (400 MHz, $CDCl_3$)

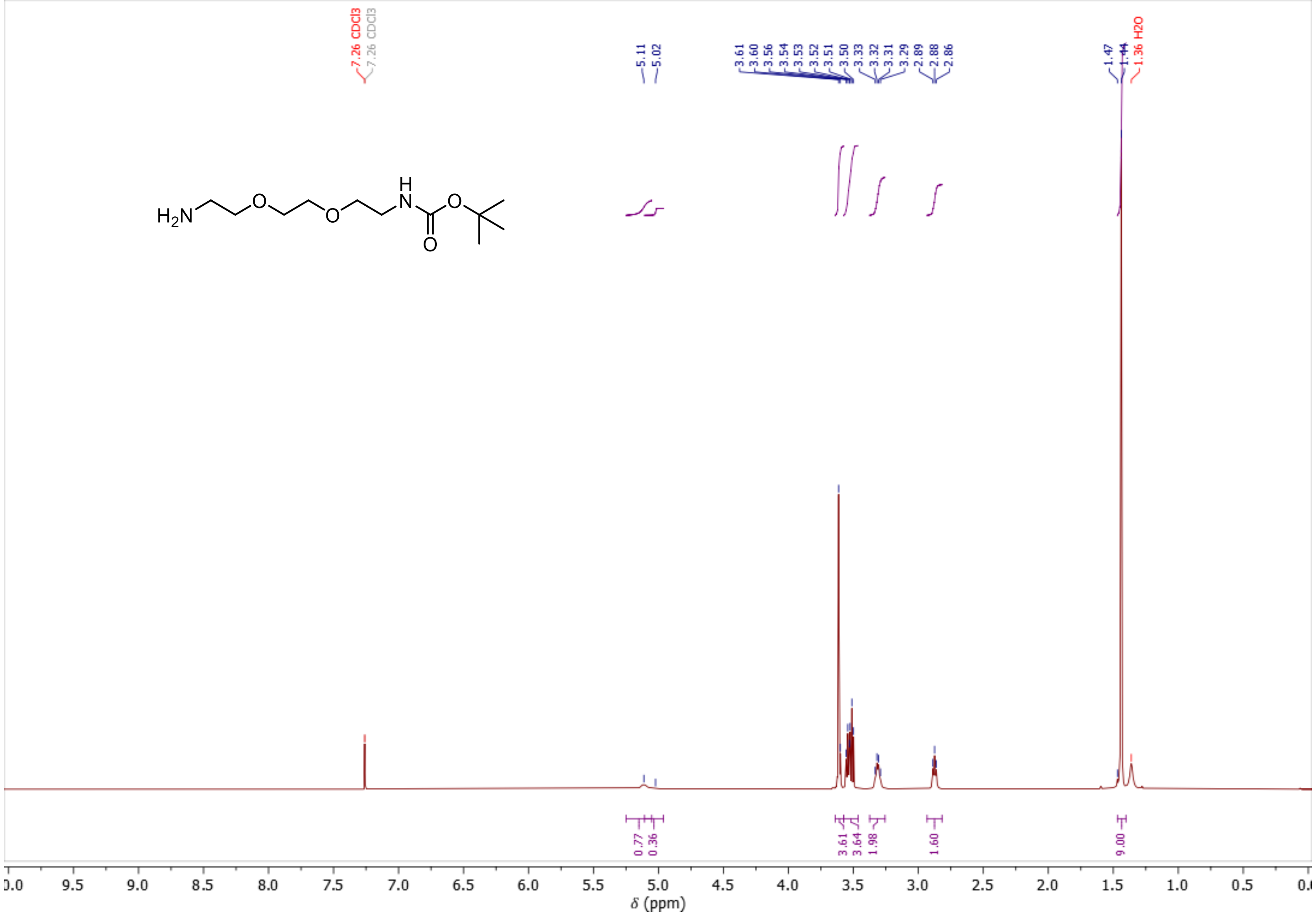

Biotin-NHS S2

^{1}H-NMR (400 MHz, DMSO)

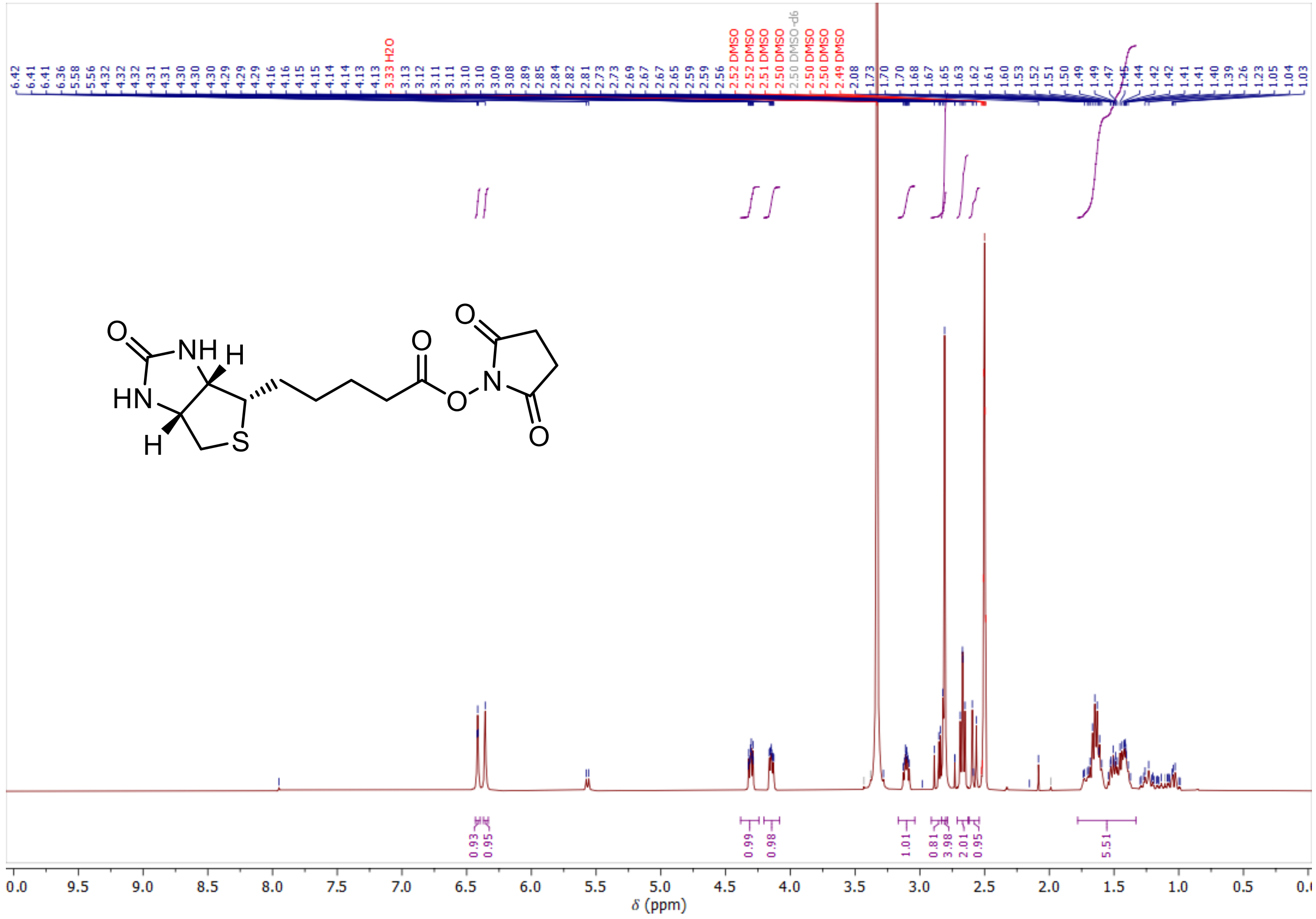

(7-(diethylamino)-3-((E)-3,14-dioxo-18-((3aS,4S,6aR)-2-oxohexahydro-1H-thieno[3,4-d]imidazol-4-yl)-7,10-dioxa-4,13-diazaoctadec-1-en-1-yl)-2-oxo-2H-chromen-4-yl)methyl prop-2-yn-1-ylcarbamate S3

^{1}H-NMR (600 MHz, $CDCl_3$)

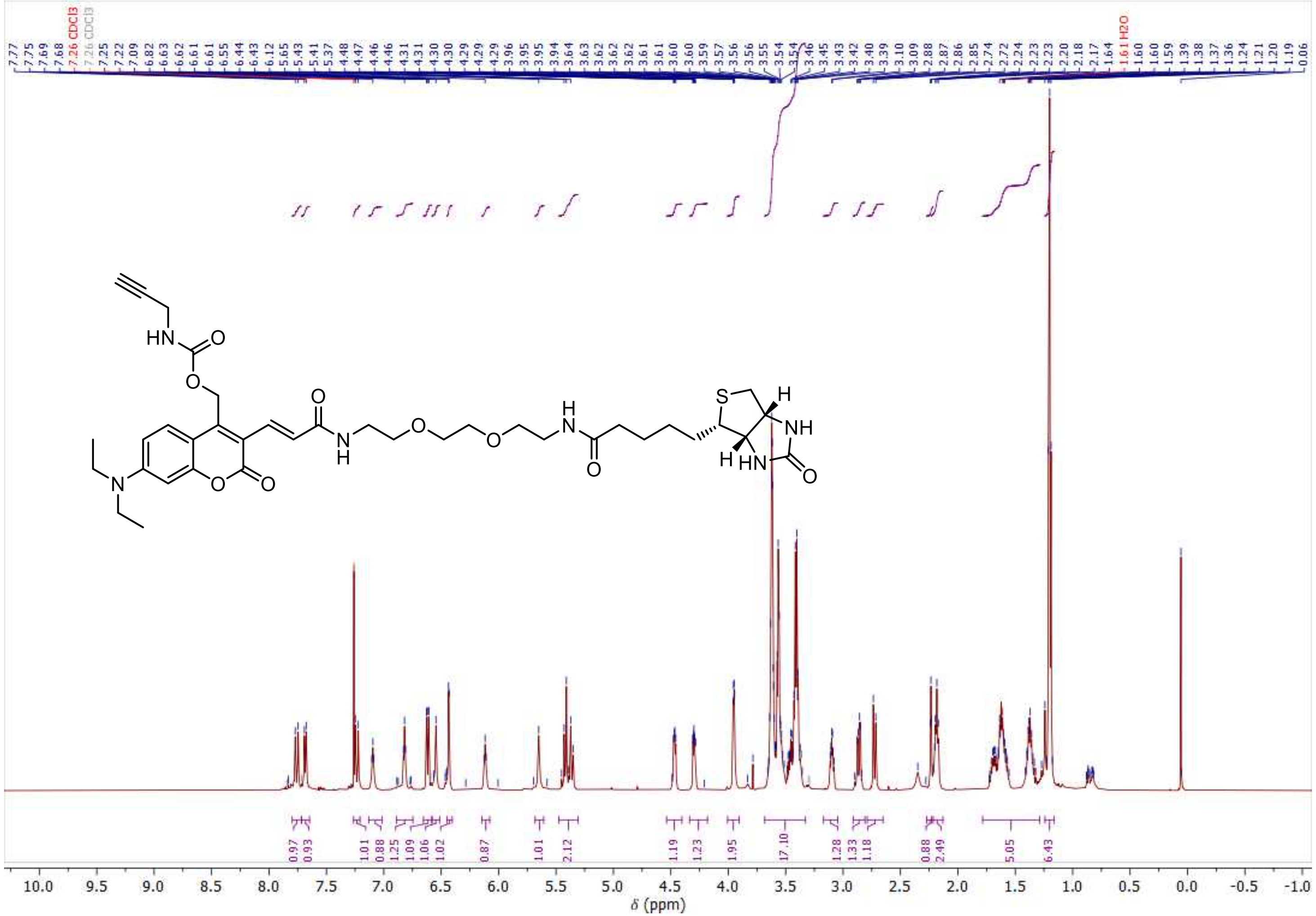

^{13}C-NMR (151 MHz, $CDCl_3$)

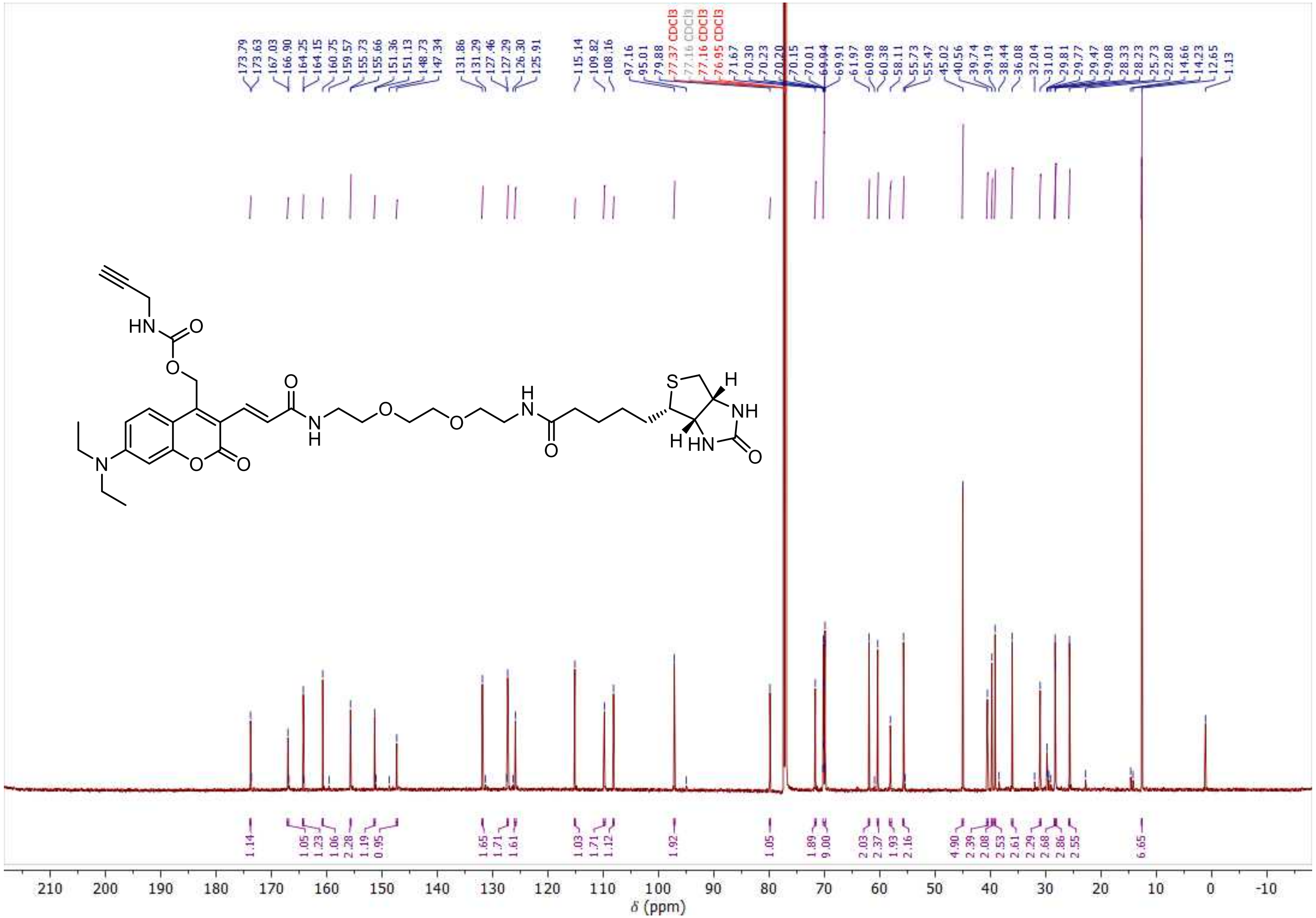

References

(1) Weinrich, T.; Gränz, M.; Grünewald, C.; Prisner, T. F.; Göbel, M. W. Synthesis of a Cytidine Phosphoramidite with Protected Nitroxide Spin Label for EPR Experiments with RNA. *European Journal of Organic Chemistry* **2017**, *2017* (3), 491–496. https://doi.org/10.1002/ejoc.201601174.

(2) Olson, J. P.; Kwon, H. B.; Takasaki, K. T.; Chiu, C. Q.; Higley, M. J.; Sabatini, B. L.; Ellis-Davies, G. C. R. Optically Selective Two-Photon Uncaging of Glutamate at 900 Nm. *Journal of the American Chemical Society* **2013**, *135* (16), 5954–5957. https://doi.org/10.1021/ja4019379.

(3) Favre, A.; Grugier, J.; Brans, A.; Joris, B.; Marchand-Brynaert, J. 6-Aminopenicillanic Acid (6-APA) Derivatives Equipped with Anchoring Arms. *Tetrahedron* **2012**, *68* (52), 10818–10826. https://doi.org/10.1016/j.tet.2011.10.100.

(4) Susumu, K.; Uyeda, H. T.; Medintz, I. L.; Pons, T.; Delehanty, J. B.; Mattoussi, H. Enhancing the Stability and Biological Functionalities of Quantum Dots via Compact Multifunctional Ligands. *Journal of the American Chemical Society* **2007**, *129* (45), 13987–13996. https://doi.org/10.1021/ja0749744.

(5) Booth, M. J.; Restrepo Schild, V.; Graham, A. D.; Olof, S. N.; Bayley, H. Light-Activated Communication in Synthetic Tissues. *Science Advances* **2016**, *2* (4), e1600056. https://doi.org/10.1126/sciadv.1600056.

(6) Smith, J. M.; Hartmann, D.; Booth, M. J. Engineering Cellular Communication between Light-Activated Synthetic Cells and Bacteria. *bioRxiv* **2022**, 2022.07.22.500923. https://doi.org/10.1101/2022.07.22.500923.

(7) Hartmann, D.; Booth, M. J. Accessible Light-Controlled Knockdown of Cell-Free Protein Synthesis Using Phosphorothioate-Caged Antisense Oligonucleotides. *Commun Chem* **2023**, *6* (1), 1–8. https://doi.org/10.1038/s42004-023-00860-2.

(8) Broome, A. M.; Bhavsar, N.; Ramamurthy, G.; Newton, G.; Basilion, J. P. Expanding the Utility of β-Galactosidase Complementation: Piece by Piece. *Molecular Pharmaceutics* **2010**, *7* (1), 60–74. https://doi.org/10.1021/mp900188e.

(9) Venkatesan, G. A.; Sarles, S. A. Droplet Immobilization within a Polymeric Organogel Improves Lipid Bilayer Durability and Portability. *Lab Chip* **2016**, *16* (11), 2116–2125. https://doi.org/10.1039/C6LC00391E.

(10) Epple, S.; Thorpe, C.; Baker, Y. R.; El-Sagheer, A. H.; Brown, T. Consecutive 5′- And 3′-Amide Linkages Stabilise Antisense Oligonucleotides and Elicit an Efficient RNase H Response. *Chemical Communications* **2020**, *56* (41), 5496–5499. https://doi.org/10.1039/d0cc00444h.
